# Diborane Reductions of CO_2_ and CS_2_ Mediated by Dicopper μ-Boryl Complexes of a Robust Bis(phosphino)-1,8-naphthyridine Ligand

**DOI:** 10.1021/acs.organomet.4c00122

**Published:** 2024-05-03

**Authors:** Matthew S. See, Pablo Ríos, T. Don Tilley

**Affiliations:** † Department of Chemistry, University of California, Berkeley, Berkeley, California 94720, United States; ‡ Chemical Sciences Division, Lawrence Berkeley National Laboratory, Berkeley, California 94720, United States; § Instituto de Investigaciones Químicas (IIQ), Departamento de Química Inorgánica, Centro de Innovación en Química Avanzada (ORFEO−CINQA), CSIC and Universidad de Sevilla, Sevilla 41092, Spain

## Abstract

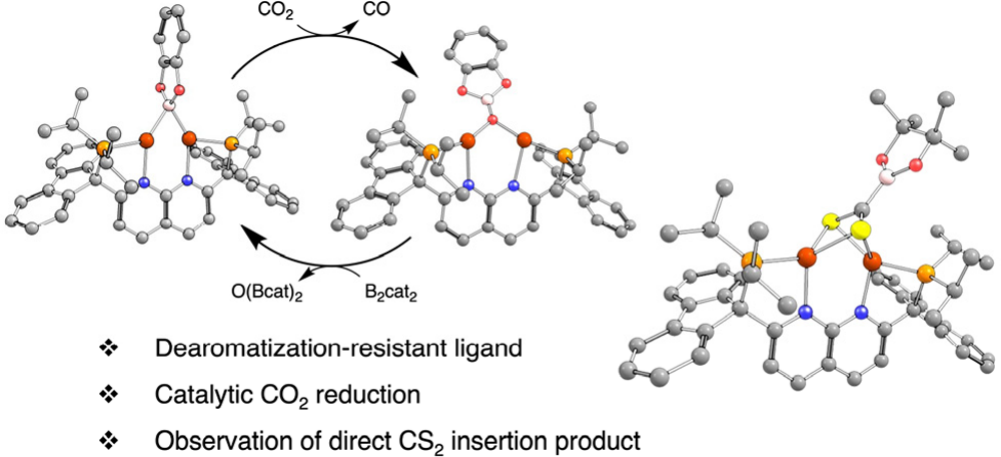

A dinucleating 1,8-naphthyridine ligand featuring fluorene-9,9-diyl-linked phosphino side arms (PNNP^Flu^) was synthesized and used to obtain the cationic dicopper complexes **2**, [(PNNP^Flu^)Cu_2_(μ-Ph)][NTf_2_]; [NTf_2_] = bis(trifluoromethane)sulfonimide, **6**, [(PNNP^Flu^)Cu_2_(μ-CCPh)][NTf_2_], and **3**, [(PNNP^Flu^)Cu_2_(μ-O^*t*^Bu)][NTf_2_]. Complex **3** reacted with diboranes to afford dicopper μ-boryl species (**4**, with μ-Bcat; cat = catecholate and **5**, with μ-Bpin; pin = pinacolate) that are more reactive in C(sp)–H bond activations and toward activations of CO_2_ and CS_2_, compared to dicopper μ-boryl complexes supported by a 1,8-naphthyridine-based ligand with di(pyridyl) side arms. The solid-state structures and DFT analysis indicate that the higher reactivities of **4** and **5** relate to changes in the coordination sphere of copper, rather than to perturbations on the Cu–B bonding interactions. Addition of xylyl isocyanide (CNXyl) to **4** gave **7**, [(PNNP^Flu^)Cu_2_(μ-Bcat)(CNXyl)][NTf_2_], demonstrating that the lower coordination number at copper is chemically significant. Reactions of **4** and **5** with CO_2_ yielded the corresponding dicopper borate complexes (**8**, [(PNNP^Flu^)Cu_2_(μ-OBcat)][NTf_2_]; **9**, [(PNNP^Flu^)Cu_2_(μ-OBpin)][NTf_2_]), with **4** demonstrating catalytic reduction in the presence of excess diborane. Related reactions of **4** and **5** with CS_2_ provided insertion products **10**, {[(PNNP^Flu^)Cu_2_]_2_[μ-S_2_C(Bcat)_2_]}[NTf_2_]_2_, and **11**, [(PNNP^Flu^)Cu_2_(μ,κ^2^-S_2_CBpin)][NTf_2_], respectively. These products feature Cu–S–C–B linkages analogous to those of proposed CO_2_ insertion intermediate.

## Introduction

Bimetallic reaction centers have attracted significant attention due to their unique chemical properties, attributed to cooperative effects resulting from metal–metal interactions.^1−3^ In catalysis, this cooperativity allows for multielectron redox processes and distinctive activations of substrates. Due to such characteristics, bimetallic active sites in both enzymes and heterogeneous materials mediate challenging and highly valuable reactions such as the oxidation of C–H bonds^4,5^ or the reduction of CO_2_.^6^

**Figure 1:**
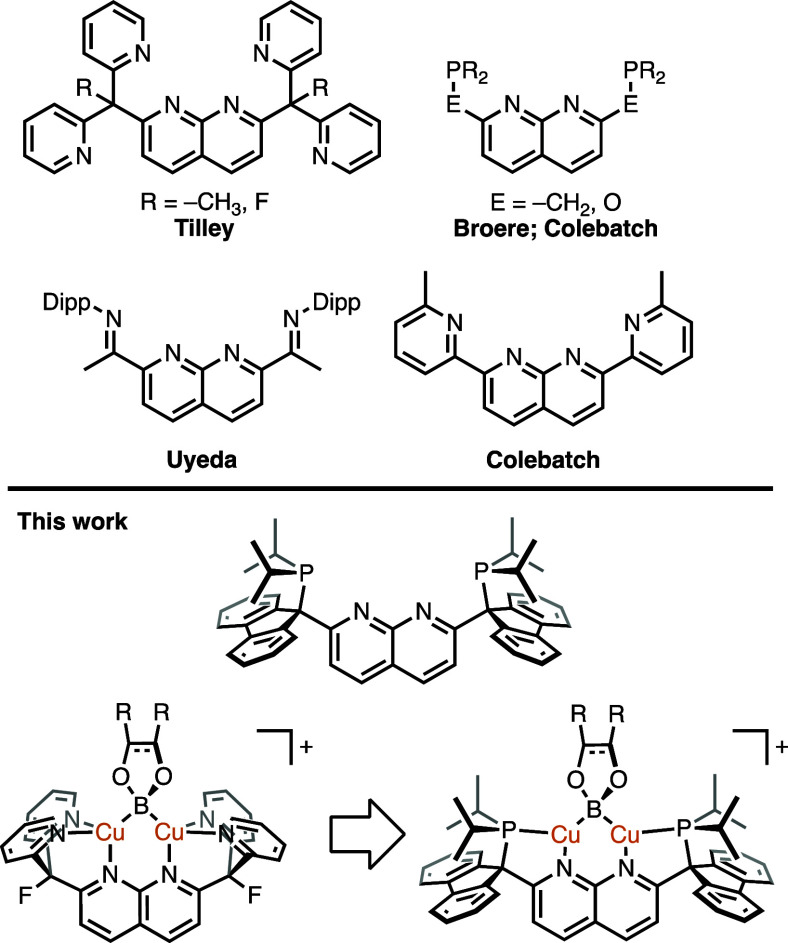
Representative examples of 1,8-napthyridine-based ligands (top; Dipp = 2,6-diisopropylphenyl) and a robust PNNP ligand to support less sterically encumbered, low-coordinate dicopper boryl complexes (bottom).^8−13^

Despite considerable effort, well-defined and tunable bimetallic moieties remain difficult to investigate, owing to the challenges in synthetic control over nuclearity, metal–metal distances, and coordination geometries. In this context, effective systems for studying bimetallic moieties utilize binucleating ligands based on the 1,8-naphthyridine platform, with various flanking side arm donors such as imides,^7^ phosphines,^8−11^ and pyridines (Figure 1, top).^12^ This laboratory has demonstrated the use of 2,7-bis(fluoro-di(2-pyridyl)methyl)-1,8-naphthyridine (DPFN) to stabilize numerous reactive moieties in dicopper(I) complexes, where stability is provided by the rigid, dinucleating nature of the ligand.^13^

A straightforward way to lower the coordination number of metal centers supported by the 1,8-naphthyridine ligand framework is *via* use of –EPR_2_ (E = O, CH_2_) side arms. This laboratory has employed both side arms in unsymmetrical 1,8-naphthyridine ligands that allow for binucleation of homo- and heterobimetallic complexes.^15,16^ Reactivity studies revealed that these side arms are readily transformed to other structures: With the −O– linker, complete loss of the group occurs by nucleophilic displacement,^15^ whereas the benzylic hydrogens of the −CH_2_– linker are susceptible to deprotonation.^15^ These ligand-based transformations have also been observed for related, symmetrical PNNP ligands reported by Broere and co-workers,^8,9^ and by the Colebatch laboratory.^11^ To thoroughly investigate the inherent properties of bimetallic complexes supported by 1,8-napthyridine-based ligands with phosphorus donors, chemically robust −EPR_2_ side arms are essential to focus reactivity at the bimetallic unit and prevent unwanted side reactions. Note that related issues pertain to pyridine-based PNP ligands, which can undergo similar side reactions. Recently, this problem was addressed by the Khusnutdinova laboratory, with synthesis of the tetramethylpyridine derivative, 2,6-(^*i*^Pr_2_PCMe_2_)_2_py, *via* multiple additions of a strong base and methylating agent as a strategy to remove the reactive benzylic hydrogens.^17^

Herein,^18^ we report the synthesis and characterization of a new 1,8-naphthyridine-based PNNP extended pincer ligand that tolerates a wide range of reaction conditions, such as the addition of bases, without chemical modification of the 1,8-naphthyridine backbone. This ligand features a chemically robust side arm linker that allows synthetic access to stable dicopper complexes with various bridging, reactive groups. Notably, this system has provided more reactive but relatively stable dicopper μ-boryl complexes that promote the catalytic reduction of CO_2_ to CO. Additionally, this dinuclear platform lends itself to the stabilization of intermediates relevant to carbon dichalcogenide reduction reactions at a dicopper center.

## Results and Discussion

### Synthesis and Metalation of PNNP^Flu^

**Scheme 1:**
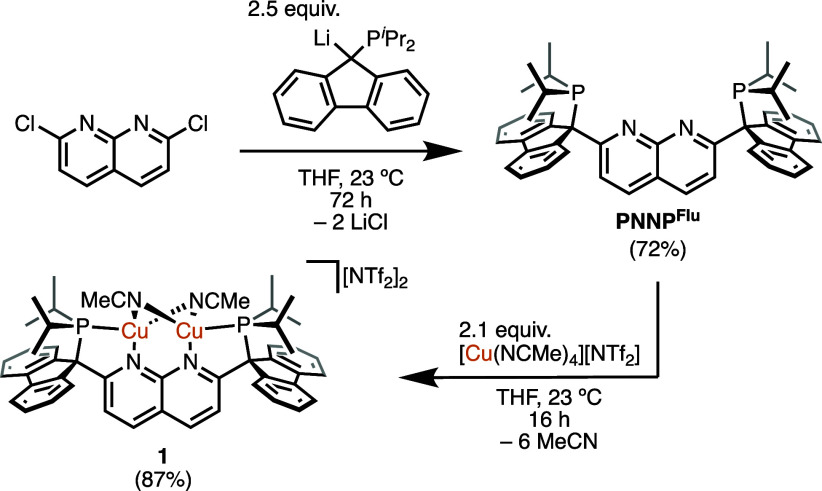
Synthesis of PNNP^Flu^ and **1** (Isolated Yield in Parentheses)

Attention was focused on a ligand design incorporating tertiary-carbon-based linkers onto the 1,8-naphthyridine scaffold. For this purpose, a fluorene-9,9-diyl linker seemed suitable given its inherent stability, rigidity, amenability to structural modification, and potential for promoting crystallinity. The desired ligand PNNP^Flu^ was obtained in 72% isolated yield by adding a THF suspension of 2,7-dichloro-1,8-naphthyridine to a THF solution of 2.5 equiv of (9-(diisopropylphosphaneyl)-fluorene-9-yl)lithium (Scheme 1). The ^31^P{H} NMR spectrum of the blue ligand exhibits a single resonance at 46.9 ppm in benzene-*d*_6_ and the corresponding ^1^H NMR spectrum is consistent with a *C*_2*v*_ symmetric species.

A dicopper complex [(PNNP^Flu^)Cu_2_(NCMe)_2_][NTf_2_]_2_ (**1**; Scheme 1) was obtained from the reaction of a solution of PNNP^Flu^ and 2.1 equiv of [Cu(NCMe)_4_][NTf_2_] in THF, which afforded an orange solution after 16 h at 23 °C. The ^31^P{^1^H} NMR spectrum in acetonitrile-*d*_3_ of the reaction mixture exhibits a new major resonance at 44.0 ppm (Figure S7).

After filtration and isolation of the resulting solid, numerous attempts to grow crystals for structural characterization or purification resulted in orange oils. The ^1^H NMR spectrum of the isolated product in acetonitrile-*d*_3_ exhibits a new singlet at 1.96 ppm that integrates to six protons, corresponding to two equivalent acetonitrile ligands (Figure S4). The narrow line width of the resonance associated with acetonitrile-*d*_3_ ligands of complex **1** in acetonitrile-*d*_3_ (90 and 80 Hz, respectively) indicates that the copper-bound ligand does not appreciably exchange with free acetonitrile on the NMR time scale.^19^ The FTIR spectrum of **1** (KBr) exhibits a weak nitrile stretch at 2275 cm^–1^ (Figure S52), comparable to that of the monoacetonitrile complex [(DPFN)Cu_2_(μ-NCMe)][NTf_2_]_2_ (2280 cm^–1^).^20^ Further characterization of this complex was hindered by the persistent presence of unidentified minor products, as determined by multinuclear NMR spectroscopic techniques, which could not be separated from **1**. Nonetheless, as demonstrated by subsequent reactivity (*vide infra*), samples of **1** obtained in this manner serve as a useful source of the [(PNNP^Flu^)Cu_2_]^2+^ core for the preparation of new complexes. Although the current data cannot distinguish between terminal versus bridging acetonitrile ligands, we favor the bridged structure (Scheme 1), given that other 1,8-naphthyridine-supported dicopper complexes exhibit this binding mode, albeit with only one acetonitrile.^20,21^

### Synthesis and Stability of [(PNNP^Flu^)Cu_2_(μ-X)]^+^ Complexes

**Scheme 2:**
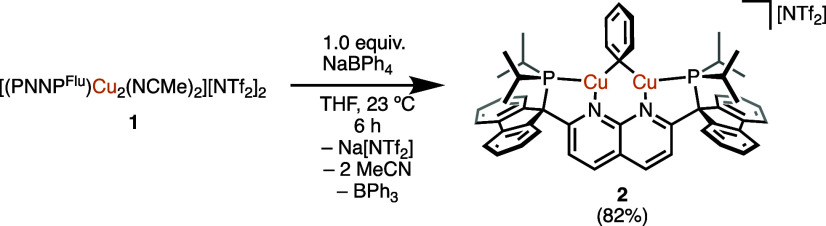
Synthesis of **2***via* Aryl Group Transfer (Isolated Yield in Parentheses)

**Figure 2:**
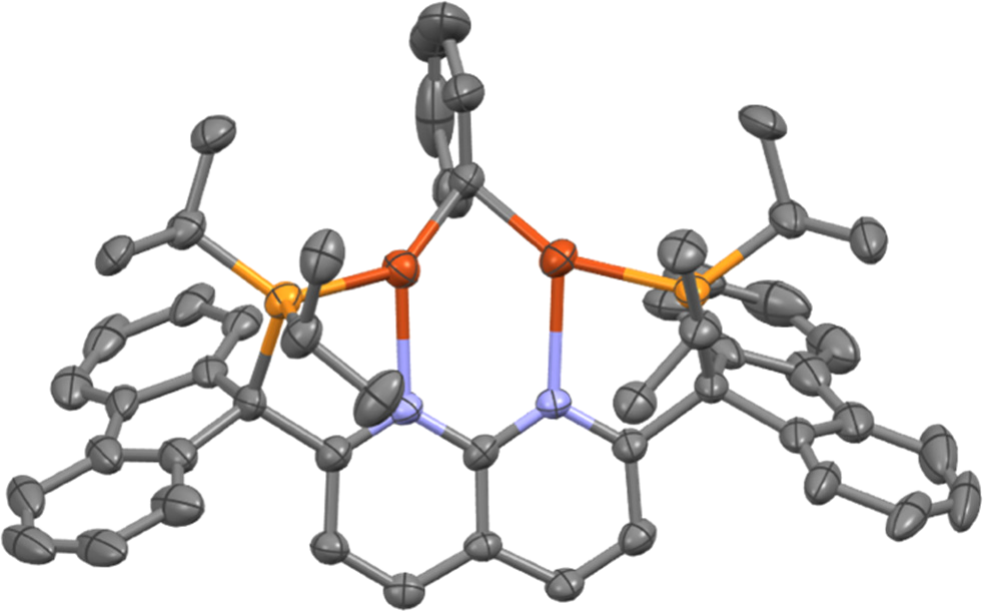
Solid-state molecular structure (50% probability ellipsoids) of the cationic fragment of 2; H atoms are omitted for clarity.

The bis(acetonitrile) complex **1** was readily converted to the phenyl derivative [(PNNP^Flu^)Cu_2_(μ-Ph)][NTf_2_] (**2**; Scheme 2), with NaBPh_4_ as a phenyl-transfer reagent, as previously described for synthesis of the DPFN-analogue [(DPFN)Cu_2(_μ-Ph)][NTf_2_].^19^ By ^1^H, ^11^B{^1^H}, and ^31^P{^1^H} NMR spectroscopy, this arylation reaction also displaces acetonitrile and liberates triphenylborane (Scheme 2). Complex **2** was structurally characterized by single-crystal X-ray diffraction (SC-XRD) analysis of crystals obtained from a THF/pentane bilayer solution (Figure 2). The average Cu–C_*ipso*_ distance, the Cu···Cu distance, and the Cu–C_*ipso*_–Cu angle of **2** in the solid-state (1.998(7) Å, 2.446(11) Å, and 76°, respectively) are comparable to corresponding metrics for other dicopper aryl complexes supported by 1,8-naphthyridine-based ligands.^15,20,22^ The bridging phenyl ligand of **2** is canted out of the Cu_2_N_(naph)2_ plane (bending angle of 136°), as observed in other neutral or cationic dicopper aryl complexes.^21^ Complex **2** in THF-*h*_8_ was heated at 80 °C for 3 days to evaluate its thermal stability. By ^1^H NMR spectroscopy, no decomposition was observed over this period, as confirmed by a hexamethyldisiloxane internal standard. Thus, dicopper complex **2** is remarkably stable in comparison to other 1,8-naphthyridine-based complexes with phosphino side arms. In examples featuring methylene-linked phosphino side arms (−CH_2_PR_2_), arylcopper derivatives readily decompose by elimination of the arene with deprotonation of the linker.^15,22^

**Scheme 3:**
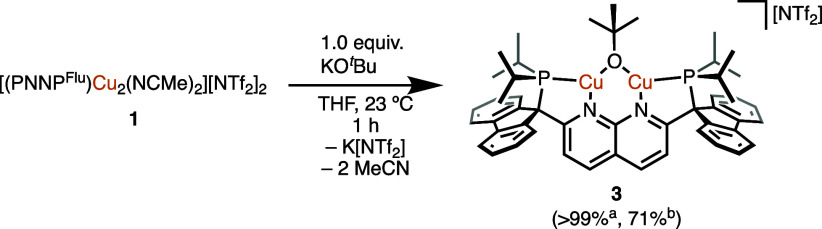
Synthesis of **3***via* Salt Metathesis Spectroscopic conversion. Isolated yield.

Previous studies on related dicopper systems have demonstrated the utility and versatility of a dicopper *tert*-butoxide fragment as a useful precursor to other dicopper complexes,^8,14,23^ and therefore [(PNNP^Flu^)Cu_2_(μ-O^*t*^Bu)][NTf_2_] (**3**) was targeted as a starting material to provide access to new [(PNNP^Flu^)Cu_2_]^2+^ complexes. Monitoring the reaction by ^1^H NMR spectroscopy indicated that **3** was generated quantitatively following addition of one equivalent of KO^*t*^Bu to a THF solution of **1** (Scheme 3; 71% isolated yield), as evidenced by the appearance of a singlet at 1.69 ppm in THF-*d*_8_ (Figure S12).

**Figure 3:**
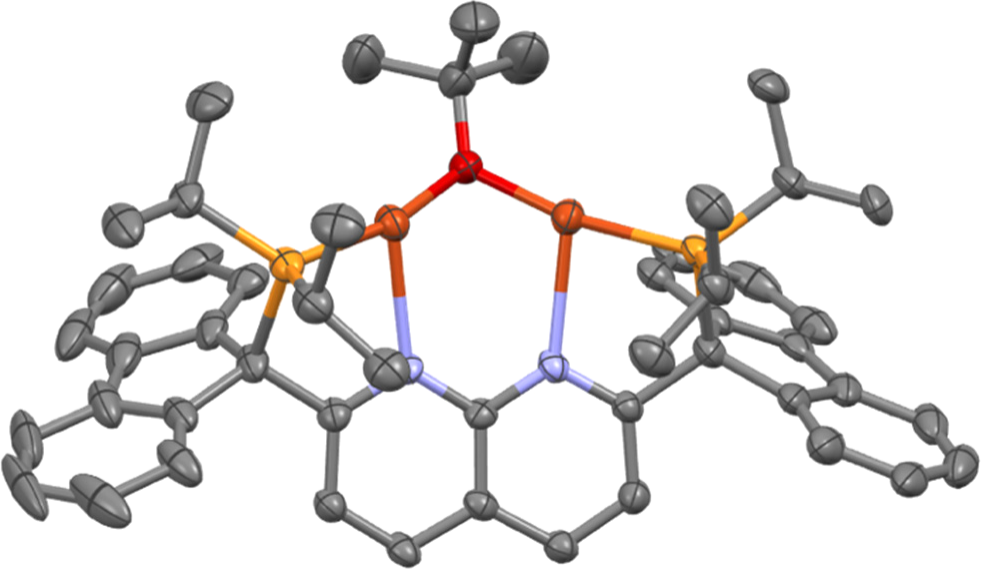
Solid-state molecular structure (50% probability ellipsoids) of the cationic fragment of **3**; H atoms are omitted for clarity.

The spectroscopic assignments for **3** are supported by the solid-state molecular structure, determined by SC-XRD experiments on crystals grown from a THF/pentane bilayer solution (Figure 3). The Cu–O distance, the Cu···Cu distance, and the Cu–O–Cu angle for **3** (1.877(2) Å, 2.843(1) Å, and 99°, respectively) are comparable to other alkoxide or aryloxide dicopper complexes supported by diphosphine-1,8-naphthyridine ligands (1.876–1.907 Å, 2.9259–3.0218 Å, and 100–106°) or DPFN (1.937–2.002 Å, 2.675–2.687 Å, and 84–88°).^8,14,24^ As with **2**, the bridging atom of **3** is bent out of the Cu_2_N_(naph)2_ plane (bending angle of 128°), in contrast to other dicopper alkoxide or aryloxide complexes where the bridging fragment lies in the Cu_2_N_(naph)2_ mean plane.^8,14,24^

Note that related dicopper *tert*-butoxide complexes supported by a 1,8-naphthyridine-based PNNP ligand have been reported by Broere et al.; nevertheless, since the ligand precursor possesses methylene linkers, only deprotonated complexes with partial or full dearomatization of the 1,8-naphthyridine backbone were obtained.^8^ Similar ligand-based reactivity is associated with analogous pyridine-based PNP complexes^25^ and has been utilized in metal–ligand cooperative reactivity to for example, activate H_2_. The comparative innocence of the PNNP^Flu^ ligand is demonstrated by the lack of ligand deprotonation and 1,8-naphthyridine dearomatization in the presence of a base such as potassium *tert*-butoxide or in the stabilities of dicopper complexes **2** and **3**.

**Scheme 4:**
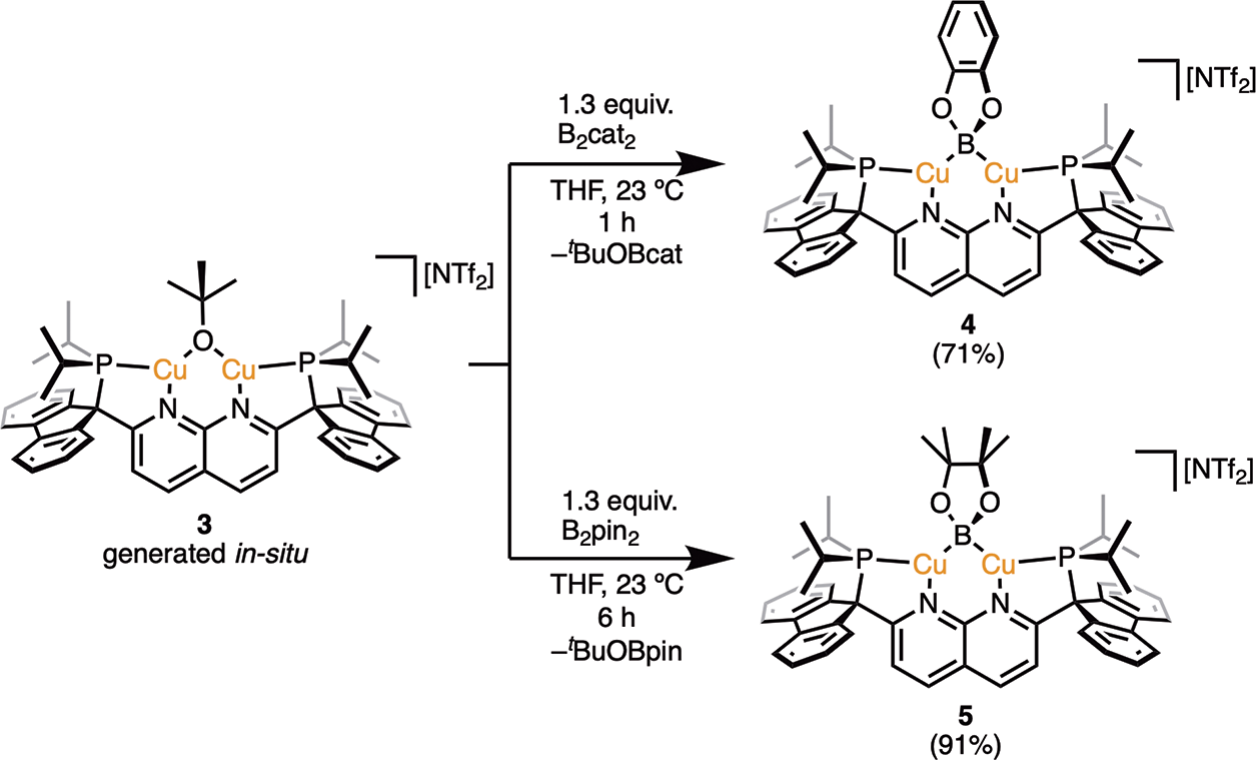
Synthesis of **4** and **5***via* Diborane Treatment of **3** (Isolated Yield in Parentheses)

**Figure 4:**
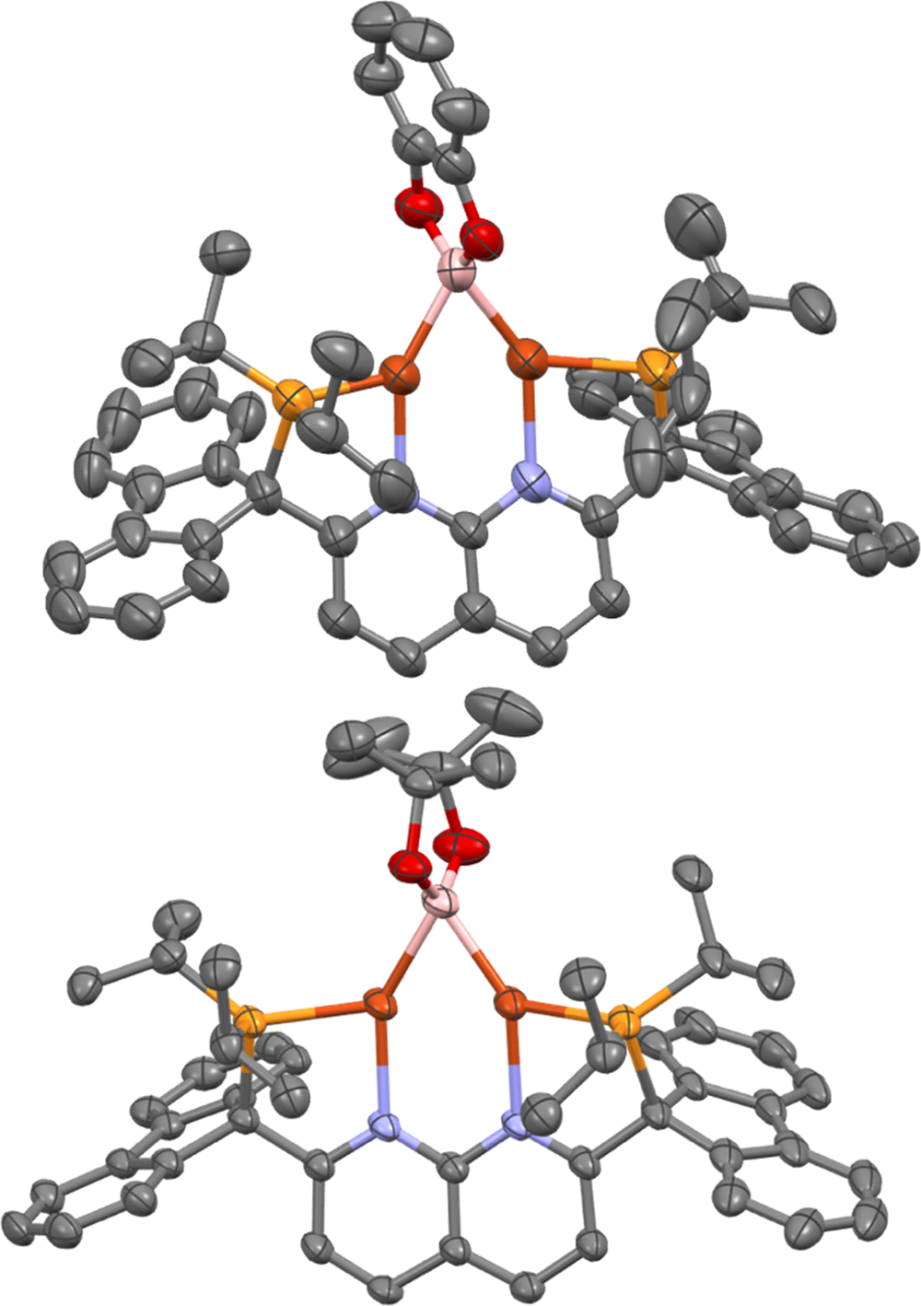
Solid-state molecular structure (50% probability ellipsoids) of the cationic fragment of **4** (top) and **5** (bottom); H atoms are omitted for clarity.

Treatment of alkoxide complex **3** with 1.3 equiv of the diborane B_2_cat_2_ (cat = catecholate) yielded a dark orange solution of the boryl complex [(PNNP^Flu^)Cu_2_(μ-Bcat)][NTf_2_] (**4**; Scheme 4), characterized by multinuclear NMR spectroscopy and SC-XRD analysis of crystals grown from a THF/pentane bilayer (Figure 4, top). Similarly, addition of 1.3 equiv of the diborane B_2_pin_2_ (pin = pinacolate) to **3** in THF resulted in a dark orange solution from which the boryl complex [(PNNP^Flu^)Cu_2_(μ-Bpin)][NTf_2_] (**5**, Scheme 4) was isolated in 91% yield and characterized by multinuclear NMR spectroscopy and SC-XRD analysis (Figure 4, bottom).

The solid-state bonding metrics for **4** and **5** are within the range of analogous parameters reported for dicopper μ-boryl complexes, including those of DPFN-supported congeneric complexes. For example, the average Cu–B distances in **4** and **5** (2.076(3) and 2.10(2) Å, respectively) fall between the extremes of comparable values for a cationic dicopper μ-boryl complex reported by Sadighi et al. ({[(SIPr)Cu]_2_(μ-Bcat)}{BF_4_}; SIPr = 1,3-bis(2,6-diisopropylphenyl)imidazolin-2-ylidene; 2.041–2.052 Å),^26^ and neutral dicopper μ-boryl species reported by Kleeberg et al. (>2.17 Å),^27,28^ and are similar to those of DPFN complexes (2.068–2.089 Å).^14^ The Cu–B–Cu bond angles of **4** and **5** (68° and 67°, respectively) lie between values observed for the cationic or neutral dicopper μ-boryl species previously mentioned (72° and *ca*. 60°, respectively),^26−28^ and are in good agreement with angles observed in DPFN analogues (67–68°).^14^ Finally, all of the reported dicopper μ-boryl complexes feature Cu···Cu distances in the range of 2.22–2.40 Å, shorter than the sum of the covalent radii for copper^29^ and complexes **4** and **5** exhibit a similar metric (2.3126(6) and 2.3075(4) Å, respectively). This suggests that short Cu···Cu distances are a characteristic of μ-boryl complexes.^30^

**Scheme 5:**
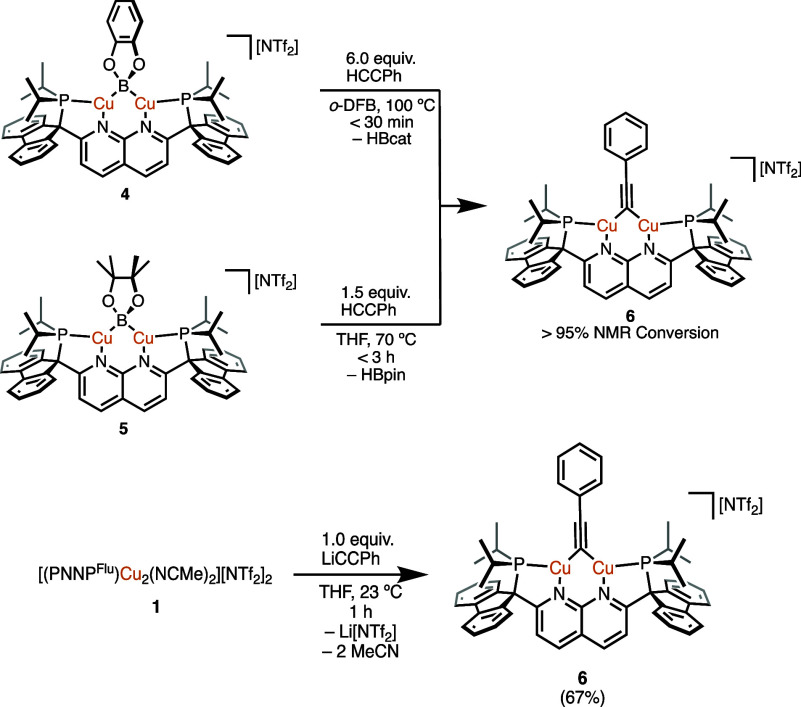
Synthesis of **6** from **4** and **5***via* C(sp)–H Bond Activation (Top) and from **1***via* Salt Metathesis (Bottom; Isolated Yield in Parentheses)

**Figure 5:**
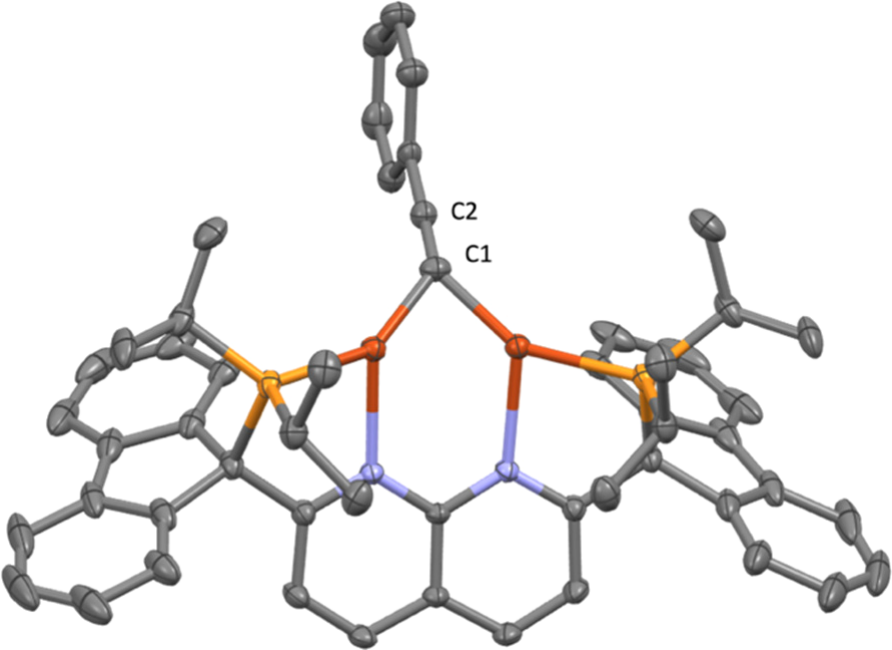
Solid-state molecular structure (50% probability ellipsoids) of the cationic fragment of **6**; H atoms are omitted for clarity.

A reaction type associated with dicopper μ-boryl complexes is activation of the C(sp)–H bonds of terminal alkynes.^14,26^ Under comparable conditions (Scheme 5, top), **4** and **5** reacted with phenylacetylene more rapidly than their DPFN analogues (100% conversion by **4** and **5** within 30 min and 3 h, respectively, compared to *ca*. 50% conversion after 22 h by the DPFN complexes)^14^ to yield the dicopper μ-alkynyl complex [(PNNP^Flu^)Cu_2_(μ-CCPh)][NTf_2_] (**6**; Scheme 5, top) as observed by ^1^H and ^31^P{^1^H} NMR spectroscopy. Complex **6** was also independently synthesized by treatment of **1** with 1.0 equiv of lithium phenylacetylide (Scheme 5, bottom), and SC-XRD analysis confirms the structural assignment (Figure 5). While the C1–C2 distance (1.219(3) Å) is akin to those of other dicopper alkynyl moieties of symmetrical and unsymmetrical 1,8-naphthyridine ligands (1.212–1.280 Å),^15,31^ the Cu···Cu distance is slightly longer at 2.4819(5) Å (compared to 2.39–2.42 Å for end-on μ-alkynyl complexes).^15,31^ As observed in **3**, there is significant bending of the bridging moiety out of the Cu_2_N_(naph)2_ plane (bending angle of 136°), in contrast to other dicopper alkynyl complexes supported by 1,8-naphthyridine ligands, in which the alkynyl group lies in the plane of the naphthyridine ring system.^15,31^ However, this deviation differs from the tilting observed in [(DPEOPN)Cu_2_(μ-CC(C_6_H_4_)CH_3_)][NTf_2_], where DPEOPN is an unsymmetrical dipyridyl-phosphine 1,8-naphthyridine ligand with −CMe(py)_2_ and −OP^*t*^Bu_2_ side arms.^15^ In the latter complex, there is substantial involvement of the acetylide π-system with one copper, resulting in a Cu···Cu distance substantially longer (2.75 Å). Notably, complex **6** does not exhibit this unsymmetrical binding mode.

To gain insight into the increased reactivity of **4** and **5**, computational methods were applied to interrogate the electronic structure and frontier molecular orbitals of the dicopper boryl fragment at the PBE0-D3BJ/6-31g(d,p)/SDD level of theory. There are slight differences in the natural charges on copper and boron in **4** and **5** when compared to other cationic dicopper boryl species, suggesting a more covalent interaction (Table 1). Despite this increase in covalency, Wiberg Bond Order analysis reveals decreased values for the Cu–B bonds in **4** and **5** compared to other cationic dicopper boryl complexes, pointing to a weakened bond. Further investigation by NLMO analysis shows minimal differences in the bond polarization between the copper and boron in **4** and **5** (Cu population: 32.97–33.89%; B population: 64.06%–65.23%) and the analogous DPFN species (Cu population: 28.60–29.67%; B population: 68.12–69.46%).^14^ This data indicates that the enhanced reactivity of **4** and **5** does not seem to be strongly rooted in changes to the bonding between copper and boron.

**Table 1:** Natural Charge and Bond Order Values for Selected Dicopper Boryl Species

parameter	**4**	**5**	[(DPFN)Cu_2_(μ-Bcat)][NTf_2_]^14^	[(DPFN)Cu_2_(μ-Bpin)][NTf_2_]^14^	{[(SIPr)Cu]_2_(μ-Bcat)}{BF_4_}^26^
natural charge on Cu	0.33	0.34	0.31	0.32	0.37/0.40
natural charge on B	0.43	0.47	0.59	0.58	0.50
Cu–B bond order	0.44	0.43	0.67	0.65	0.58

Furthermore, it does not appear that the enhanced reactivity differences stem from the steric profiles about the Cu_2_B fragment.^32^ For comparison, the steric congestion around the boron atoms of **4** and **5** (%*V*_bur_ = 71.8–72.2%) is slightly less than that of their DPFN analogues (%*V*_bur_ = 78.7–83.0%),^14^ while {[(SIPr)Cu]_2_(μ-Bcat)}{BF_4_} possesses a more sterically encumbered boron atom (%*V*_bur_ = 83.4%) despite reacting under milder conditions (−35 °C over 8 h with 2 equiv of phenylacetylene)_._^26^ Additionally, steric congestion about copper atoms is approximately the same for the copper atoms of **4** (%*V*_bur_ = 80.2%), [(DPFN)Cu_2_(μ-Bcat)][NTf_2_] (%*V*_bur_ = 80.4%), and ({[(SIPr)Cu]_2_(μ-Bcat)}{BF_4_}) (%*V*_bur_ = 81.9%). A potential explanation for the difference in reactivities for these copper boryls is that the rigid nature of the 1,8-naphthyridine backbones of PNNP^Flu^ and DPFN precludes dissociation into more reactive monomers, which may occur for the SIPr-supported copper boryl species.^26−28,33^ Additionally, the difference in coordination number around the copper centers of rigid dicopper boryl complexes may be a significant contributor to the increase in reactivity for **4** and **5**.

**Scheme 6:**
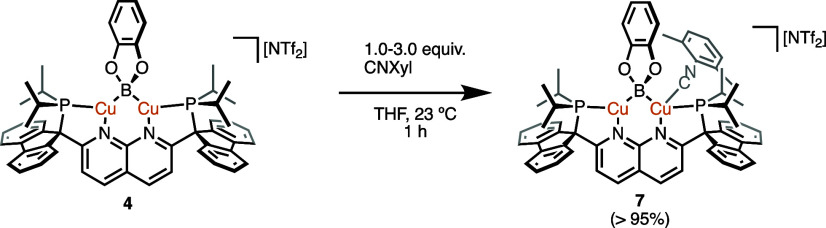
Synthesis of **7** from **4***via* CNXyl Coordination (Isolated Yield in Parentheses)

To probe the accessibility of the copper centers to additional substrates, the reaction of **4** with xylyl isocyanide (CNXyl) was examined. The stoichiometric reaction produced [(PNNP^Flu^)Cu_2_(μ-Bcat)(CNXyl)][NTf_2_] (**7**; Scheme 6) as a single species, initially identified by the appearance of a new ^31^P{^1^H} resonance at 52.6 ppm in THF-*d*_8_ (Figure S33), with full conversion after 1 h. The ^1^H NMR spectrum of analytically pure **7** displays sharp and broad resonances in the aromatic region consistent with *C*_2*v*_ symmetry and equivalent copper sites in THF-*d*_8_ at 292 K. This is consistent with a fluxional bridging ligand or a dynamic process that averages sites for coordinated CNXyl on the NMR time scale at 292 K.^19^ In contrast, [(DPFN)Cu_2_(μ-Bcat)][NTf_2_] does not react with an equivalent of CNXyl over 24 h at 80 °C in THF (monitored by ^1^H NMR spectroscopy). Titration of **4** with additional CNXyl in 0.5 equiv increments, up to 2.0 equiv, did not lead to further coordination of CNXyl, as evidenced by the lack of a shift in ^1^H NMR resonances beyond addition of one equiv.

As with **4**, complex **5** reacted completely with 1.0 equiv of CNXyl after 5 min at 23 °C to give a new phosphorus-containing species as observed by ^31^P{^1^H} NMR spectroscopy. The ^1^H NMR spectrum of the reaction mixture reveals broad resonances in the aromatic region at 292 K corresponding to a species of apparent *C*_2*v*_ symmetry (Figure S49). Unfortunately, attempts to crystallize and structurally characterize this product were unsuccessful as it readily decomposes in solution at 23 °C. Similarly, samples of **7** stored at 23 °C in the solid state decompose slowly over a few months.

**Figure 6:**
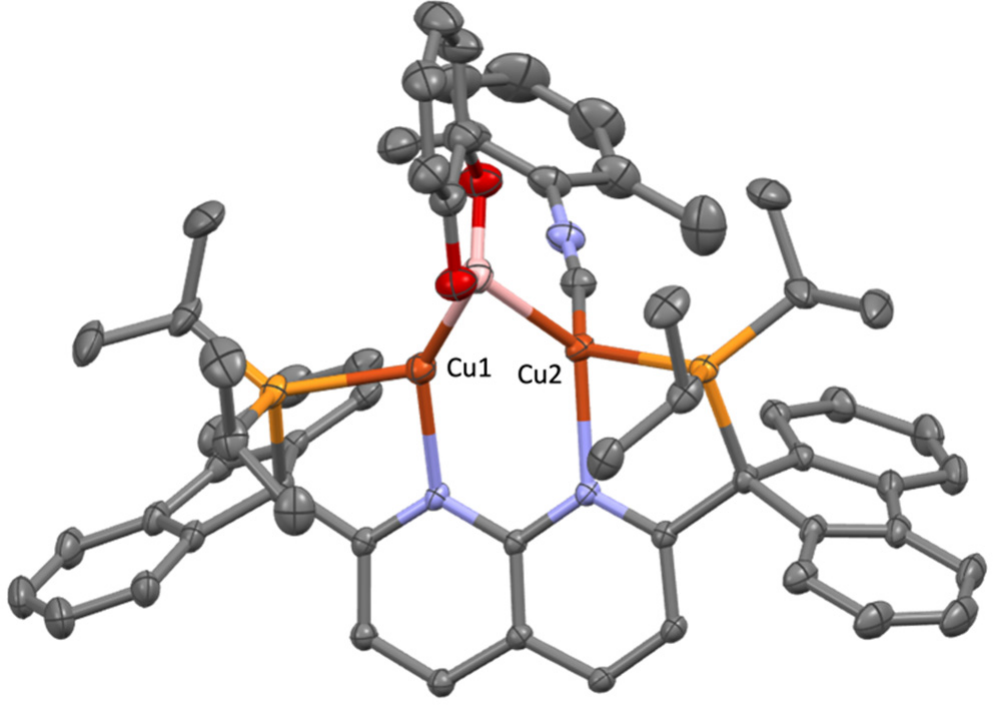
Solid-state molecular structure (50% probability ellipsoids) of the cationic fragment of **7**; H atoms are omitted for clarity.

Single-crystal X-ray diffraction analysis of **7** identified the molecular structure as possessing a terminally bound CNXyl ligand on one of the copper centers (Figure 6). The Cu···Cu distance and Cu–B–Cu bond angle of **7** (2.3870(5) Å and 69°, respectively) are similar to corresponding values for related dicopper boryl species (*vide supra*). However, there is significant desymmetrization in the Cu–B bond distances with the Cu2–B bond being significantly elongated to 2.210(3) Å, with respect to the 2.018(3) Å value for the Cu1–B bond distance. The FTIR spectrum of **7** exhibits a strong ν_C≡N_ stretching band at 2128 cm^–1^, a value above that for the free ligand (2121 cm^–1^).^34^ This indicates that CNXyl binds to copper primarily as a strong σ donor, with little back-donation, and a strong electrostatic effect increases the ν_C≡N_ frequency.^35^ Consistently, the metrical parameters of the isocyanide ligand (1.162(3) Å) reflect the presence of a C≡N triple bond.^36^

### Reactions of **4** and **5** with CO_2_

**Scheme 7:**
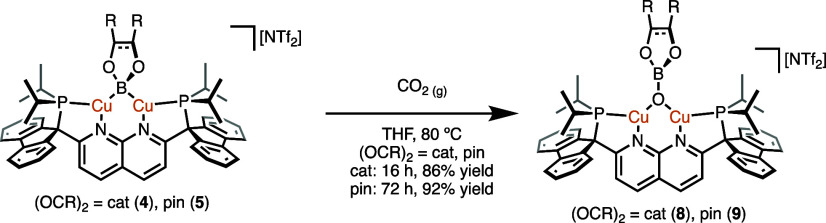
Deoxygenative Reduction of CO_2_ by **4** and **5** to Yield **8** and **9**, Respectively

**Figure 7:**
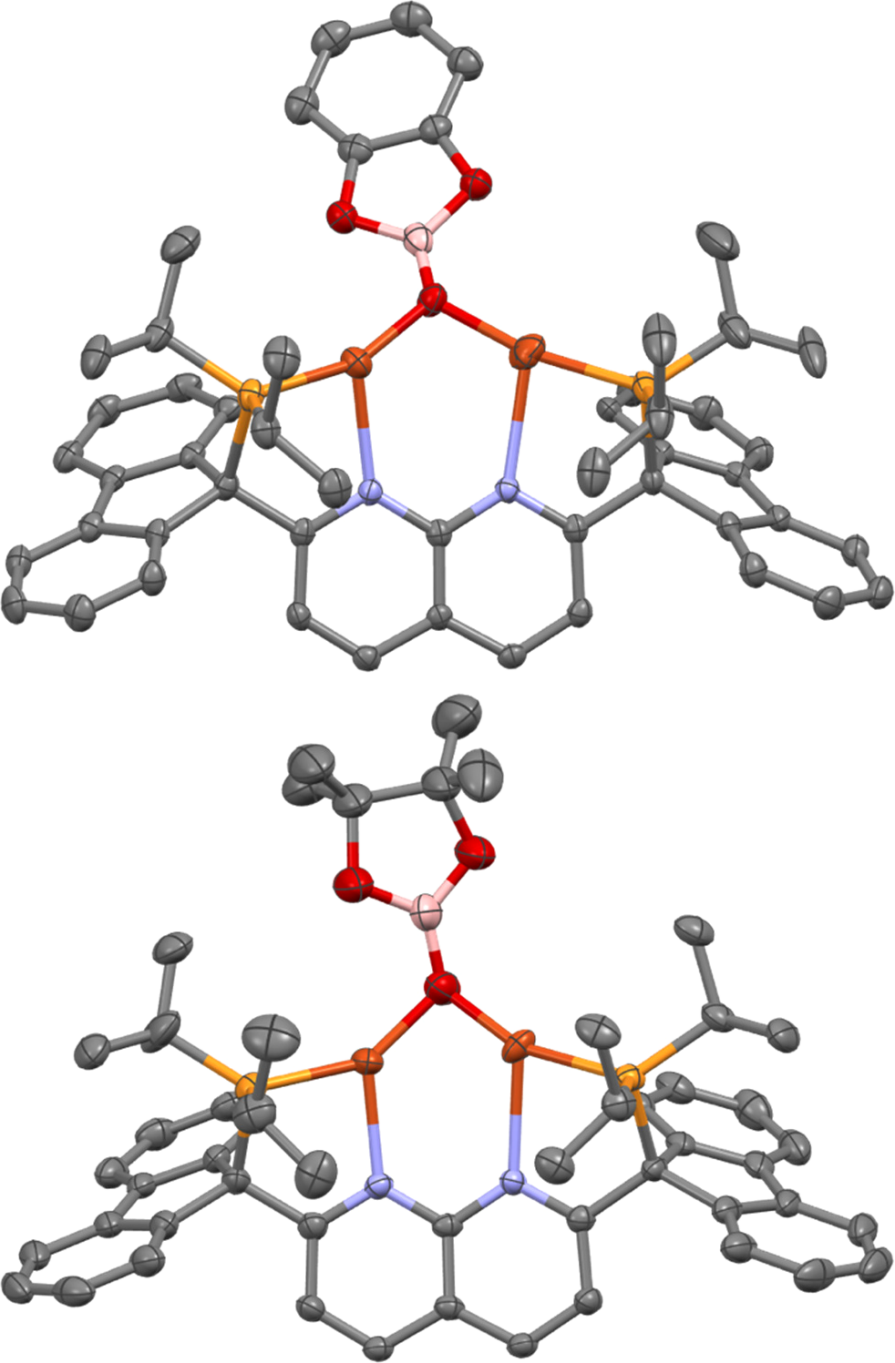
Solid-state molecular structure (50% probability ellipsoids) of the cationic fragment of **8** (top) and **9** (bottom); H atoms are omitted for clarity. Only the major disorder component of the pinacolate fragment of **9** is shown.

The reduction of CO_2_ by copper boryl complexes has received considerable attention since Sadighi et al. first demonstrated stoichiometric and catalytic examples that produce a copper borate complex with concomitant generation of CO.^37^ To investigate related CO_2_ reductions with a rigid dicopper core, boryl complex **4** was subjected to 1 atm of carbon dioxide over the course of 16 h at 80 °C. This gave the dicopper borate complex [(PNNP^Flu^)Cu_2_(μ-OBcat)][NTf_2_] (**8**; Scheme 7), identified by multinuclear NMR spectroscopy and SC-XRD analysis (Figure 7, top). Similarly, complex **5** reacted under 1 atm of carbon dioxide over the course of 72 h at 80 °C to give the analogous dicopper borate complex [(PNNP^Flu^)Cu_2_(μ-OBpin)][NTf_2_] (**9**; Scheme 7; Figure 7, bottom).

As compared to **3**, complex **9** exhibits very similar bonding metrics for the Cu···Cu and Cu–O bond distances (avg 2.71 Å and avg 1.86 Å, respectively), and an average Cu–O–Cu bond angle of 93°. Complex **8** exhibits comparable but slightly different metrics, with average Cu···Cu and Cu–O bond distances of 2.91 and 1.88 Å, respectively, and an average Cu–O–Cu bond angle of 100°.

**Scheme 8:**
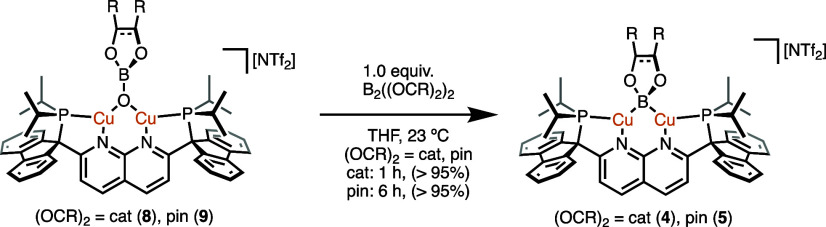
Stoichiometric Regeneration of **4** and **5***via* Reaction with Diboranes (Spectroscopic Conversion in Parentheses)

Both **8** and **9** stoichiometrically react with the respective diborane progenitor (B_2_cat_2_ for **8** and B_2_pin_2_ for **9**) to quantitatively regenerate **4** and **5** (Scheme 8). These reactions, along with those that produce the borates by activation of CO_2_, suggested the potential for catalytic reductions of CO_2_ to CO, as previously observed with monocopper boryl complexes supported by *N*-heterocyclic carbene ligands.^37,38^ Indeed, heating a 20 mol % THF solution of **4** and B_2_cat_2_ under an atmosphere of CO_2_ to 80 °C for 144 h resulted in 85% conversion of the B_2_cat_2_ substrate, equivalent to 4.25 turnovers (Figure S51). While O(Bcat)_2_ was identified as the boron-containing product of catalysis, the bis-boryl ether decomposes under the catalytic conditions (Figure S51). Monitoring the catalysis by ^1^H NMR spectroscopy revealed that **4** is the resting state during catalysis, and no accumulation of **9** was observed. In contrast, **5** did not exhibit catalytic reactivity under identical conditions. Complexes **4** and **5** represent the first well-defined dicopper boryl complexes shown to reduce CO_2_. For comparison, the cationic dicopper boryl species {[(SIPr)Cu]_2_(μ-Bcat)}{BF_4_} does not react with CO_2_ at ambient temperatures, while DPFN-supported dicopper boryl species give rise to a mixture of species at elevated temperatures.^14,27^ In the context of dicopper boryls, complexes **4** and **5** strike a balance between reactivity/catalysis and catalyst stability.

### Reactions of **4** and **5** with CS_2_

**Figure 8:**
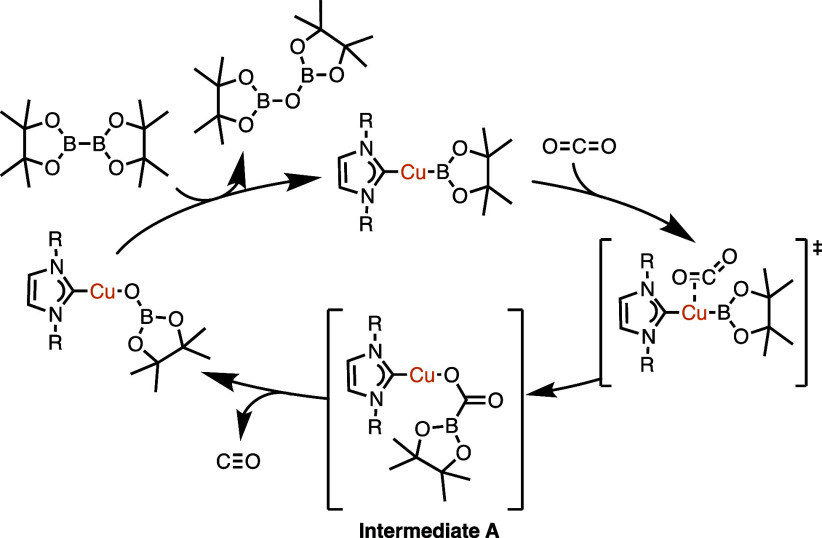
DFT-calculated catalytic cycle of the reduction of CO_2_ to CO by a copper(I) boryl complex.^39^

The reduction of CO_2_ by bimetallic complexes **4** and **5** are slower than related transformations of monocopper species (16 h at 80 °C by **4** and **5** compared to <10 min at ambient temperature by (IPr)CuBpin, IPr = 1,3-bis(2,6-diisopropylphenyl)imidazol-2-ylidene).^37^ Based on DFT studies reported by Lin and Marder et al., the monocopper mechanism involves CO_2_ insertion into a Cu–B bond to produce unobserved intermediate **A**, as depicted in Figure 8.^39^

Attempts to observe a CO_2_-insertion product analogous to intermediate **A**, starting from **4** or **5**, by ^1^H, ^31^P{^1^H}, or ^11^B{^1^H} NMR spectroscopy, were unsuccessful. For this reason, analogous reactions with the CO_2_ surrogate CS_2_ were examined to develop a better sense for the insertion chemistry associated with the dicopper systems. Computational studies indicate that although CS_2_ is more electrophilic than CO_2_, reactions of CS_2_ with transition metal nucleophiles are slower than analogous reactions with CO_2_.^40^ Additionally, carbon disulfide has been successfully implemented as a model for CO_2_ binding to dicopper, shedding light on potential activation strategies for CO_2_.^41^ Thus, CS_2_ appeared to be well-suited as a model substrate for “trapping” a direct insertion product.

**Scheme 9:**
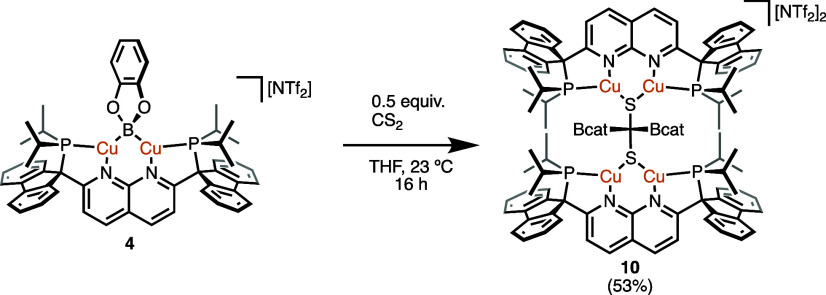
Double Borylation of CS_2_ by **4** (Isolated Yield in Parentheses)

**Figure 9:**
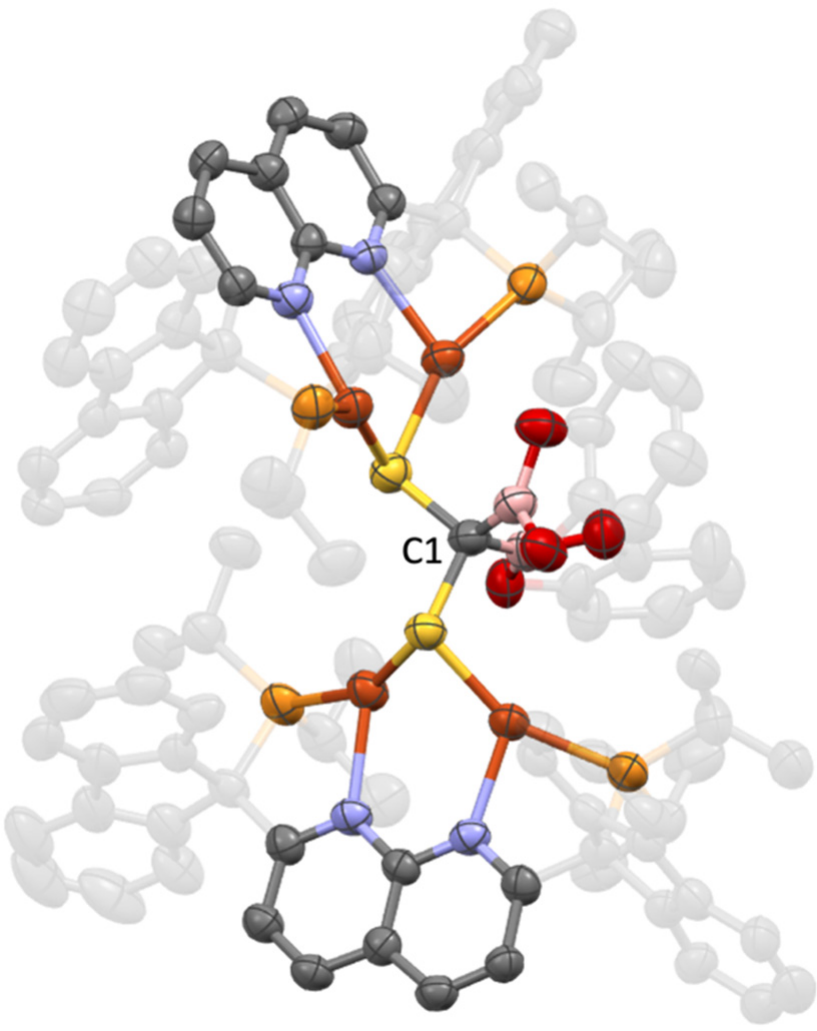
Solid-state molecular structure (50% probability ellipsoids) of the dicationic fragment of **10**; H atoms are omitted and extraneous sections of the PNNP^Flu^ ligands and catecholate fragments made transparent for clarity.

A THF solution of **4** reacted with 0.5 equiv of CS_2_, resulting in spontaneous deposition of red crystals of {[(PNNP^Flu^)Cu_2_]_2_[μ-S_2_C(Bcat)_2_]}[NTf_2_]_2_ (**10**; Scheme 9) from the reaction mixture, as determined by SC-XRD analysis (Figure 9). Complex **10** possesses two [(PNNP^Flu^)Cu_2_]^2+^ units supporting bis(catecholboryl)methanedithiolate, a doubly borylated CS_2_ fragment. The average C–S bond distances are significantly elongated (1.854(5) Å) relative to those in CS_2_ (1.555(3) Å),^42^ suggesting single bond character. Additionally, the τ_4_ parameter of C1 corresponds for a tetrahedral carbon center (τ_4_ = 0.97),^43^ indicating C(sp^3^) character. This 1,8-naphthyridine-supported dicopper sulfide complex exhibits average Cu···Cu and Cu–S bond distances (2.74 and 2.17 Å, respectively), and an average Cu–S–Cu bond angle (78°) that are similar to those of other dicopper 1,8-naphthyridine-supported complexes.^13^ Unfortunately, solution-state characterization and reactivity studies of **10** were precluded by insolubility of the analytically pure material in typical organic solvents.

**Scheme 10:**
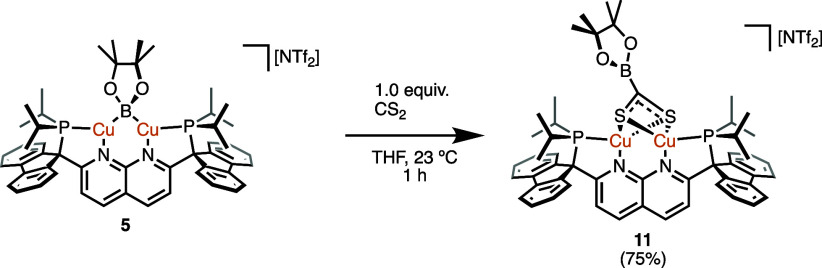
Borylation of CS_2_ by **5** (Isolated Yield in Parentheses)

**Figure 10:**
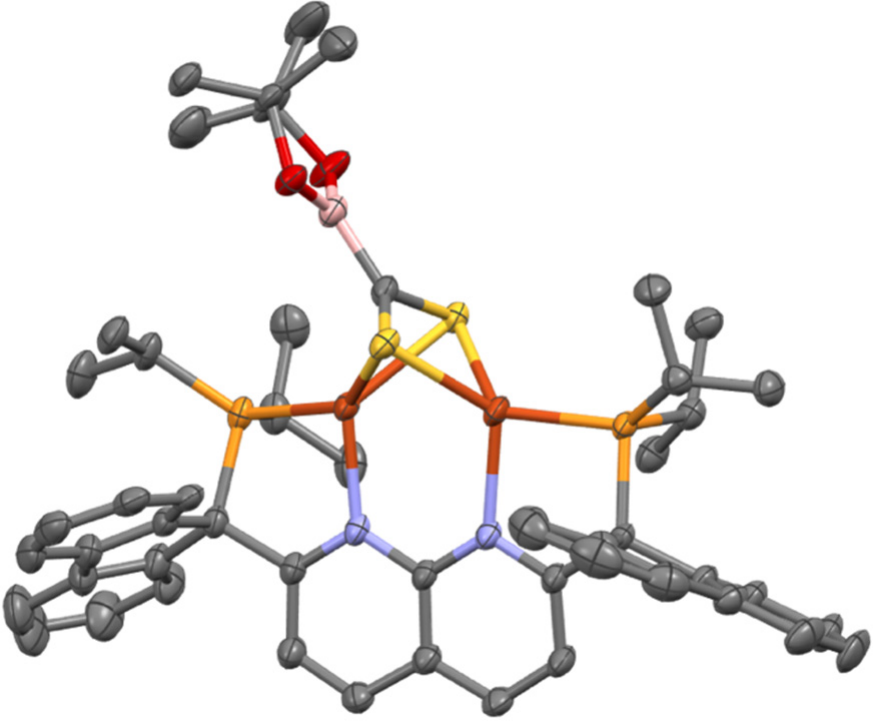
Solid-state molecular structure (50% probability ellipsoids) of the cationic fragment of **11**; H atoms are omitted for clarity. Only the major disorder component of the 2,3-dimethylbutane-2,3-diolate fragment of **11** is shown.

Reaction of **5** with 1 equiv of CS_2_ gave a red solution of a soluble PNNP^Flu^-containing species, by both ^1^H and ^31^P{^1^H} NMR spectroscopy (Figures S44 and S48). Single-crystal X-ray diffraction analysis reveals the product to be a dicopper dithioacetate complex, [(PNNP^Flu^)Cu_2_(μ,κ^2^-S_2_CBpin)][NTf_2_] (**11**; Scheme 10, Figure 10). While complex **10** contains a doubly borylated CS_2_ fragment supported by two dicopper cores, the formation of **11** is evidently arrested after one insertion event, emulating intermediate **A** (Figure 8). The asymmetric unit of crystalline **11** possesses two [(PNNP^Flu^)Cu_2_(μ,κ^2^-S_2_CBpin)][NTf_2_] molecules. For comparison, the average C–S bond distance (1.72 Å) is between those of **10** and free CS_2_,^43^ suggesting an sp^2^-hybridized carbon center at the core of the dithioacetate ligand, which bridges the copper atoms through the sulfur atoms. The dithioacetate sulfur atoms symmetrically bridge the two metal centers instead of the monodentate bridging mode previously observed with formate and pyridonate between two copper atoms where each heteroatom is ligated to one metal.^44^ Notably, there is significant tilting of the −CBpin group toward one of the copper centers, as reflected in divergent, averaged Cu–C–B angles (167, 126°). The average Cu···Cu and Cu–S bond distances (2.71 and 2.38 Å, respectively) and the average Cu–S–Cu bond angles (69°) of **11** are similar to analogous parameters in other dicopper naphthyridine-supported complexes.

With the characterization of **10** and **11**, intermediate **A** in the CO_2_ reduction mechanism is experimentally and structurally supported. It also appears that an initial insertion product such as intermediate **A** can react further with an additional copper boryl equivalent to give a “double insertion” product (**11**). Both **10** and **11** feature a Cu–S–C–B linkage analogous to the boranocarbonate fragment of intermediate **A** in the DFT-supported mechanism for CO_2_ reduction by a copper boryl species. To the best of our knowledge, complexes **10** and **11** are the only structurally characterized copper boranodithiocarbonate and bis(boryl)methanedithiolate. These complexes support a mechanism requiring a boryl-migration step that was previously supported by computation, but experimentally unsubtantiated.^39^

## Conclusions

The new ligand PNNP^Flu^ was designed and synthesized to tolerate a wider range of reaction conditions, allowing for isolation of the role of metal–metal cooperativity in the context of bimetallic chemistry. This chemically robust ligand was employed in the synthesis of thermally stable organodicopper, dicopper μ-*tert*-butoxide, and dicopper μ-boryl complexes (**2**–**6**). The latter exhibited an enhanced ability to activate C(sp)–H bonds compared to other rigid dicopper boryl species. Computational investigation of the Cu_2_B fragments of **4** and **5** suggests that the enhanced reactivity can be attributed to the low coordination numbers enforced by the PNNP framework, as demonstrated by the coordination of CNXyl to **4**.

Complexes **4** and **5** deoxygenate and reduce CO_2_ to afford dicopper borate complexes (**8** and **9**, respectively), with **4** capable of affecting this reaction catalytically with bis(catecholato)diboron. Complexes **4** and **5** also react with CS_2_ to yield a bis(boryl)methanedithiolate fragment and a boryldithioacetate, experimentally supporting a computationally determined mechanism by which the previously mentioned CO_2_ reduction proceeds through a boranocarbonate intermediate. This arrested reactivity in the reduction of CS_2_ experimentally supports a mechanism by which a copper boranocarbonate intermediate is formed prior to boryl migration and CO extrusion in the analogous reduction of CO_2_ with copper boryl species. Notably, this work demonstrates the ability to tune the reactivity of bimetallic complexes by modification of the dinucleating ligand, striking a balance between efficient reactivity and stability. Current efforts are being focused on the expansion of these design principles in tuning reactivity patterns for other dinuclear complexes.

## Supporting Information Available

Experimental methods, details of synthesis, details of crystallography, details of calculations, NMR spectra, IR spectra (PDF)
Cartesian coordinates of the optimized structures of complexes **4**, **5**, **10**, and **11** (XYZ)

## Supplementary Material

om4c00122_si_001.pdf

om4c00122_si_002.xyz

## References

[ref1] Gramigna K. M.; Dickie D. A.; Foxman B. M.; Thomas C. M. Cooperative H_2_ Activation across a Metal-Metal Multiple Bond and Hydrogenation Reactions Catalyzed by a Zr/Co Heterobimetallic Complex. J. Am. Chem. Soc. 2019, 9, 3153–3164. 10.1021/acscatal.8b04390.

[ref2] Prat J. R.; Gaggioli C. A.; Cammarota R. C.; Bill E.; Gagliardi L.; Lu C. C. Inorg. Chem. 2020, 59, 14251–14262. 10.1021/acs.inorgchem.0c02041. 32954721

[ref3] Jayarathne U.; Parmelee S. R.; Mankad N. P. Small Molecule Activation Chemistry of Cu-Fe Heterobimetallic Complexes Toward CS_2_ and N_2_O. Inorg. Chem. 2014, 53, 7730–7737. 10.1021/ic501054z. 24979669

[ref4] Basch H.; Mogi K.; Musaev D. G.; Morokuma K. Mechanism of the Methane → Methanol Conversion Reaction Catalyzed by Methane Monooxygenase: A Density Functional Study. J. Am. Chem. Soc. 1999, 121, 7249–7256. 10.1021/ja9906296.

[ref5] Woertink J. S.; Smeets P. J.; Groothaert M. H.; Vance M. A.; Sels B. F.; Schoonheydt R. A.; Solomon E. I. A [Cu_2_O]^2+^ Core in Cu-ZSM-5, the Active Site in the Oxidation of Methane to Methanol. Proc. Natl. Acad. Sci. U. S. A. 2009, 106, 18908–18913. 10.1073/pnas.0910461106. 19864626 PMC2776445

[ref6] Jeoung J.; Dobbek H. Carbon Dioxide Activation at the Ni,Fe-Cluster of Anaerobic Carbon Monoxide Dehydrogenase. Science 2007, 318, 1461–1464. 10.1126/science.1148481. 18048691

[ref7] Zhou Y.; Hartline D. R.; Steiman T. J.; Fanwick P. E.; Uyeda C. Dinuclear Nickel Complexes in Five States of Oxidation Using a Redox-Active Ligand. *Inorg*. Chem. 2014, 53, 11770–11777. 10.1021/ic5020785. 25337719

[ref8] Kounalis E.; Lutz M.; Broere D. L. Cooperative H_2_ Activation on Dicopper(I) Facilitated by Reversible Dearomatization of an “Expanded PNNP Pincer” Ligand. Chem.—Eur. J. 2019, 25, 13280–13284. 10.1002/chem.201903724. 31424132 PMC6856846

[ref9] Scheerder A. R.; Lutz M.; Broere D. L. Unexpected Reactivity of a PONNOP ‘Expanded Pincer’ Ligand. Chem. Commun. 2020, 56, 8198–8201. 10.1039/D0CC02166K. 32395727

[ref10] Delaney A. R.; Yu L.-J.; Coote M. L.; Colebatch A. L. Synthesis of an Expanded Pincer Ligand and Its Bimetallic Coinage Metal Complexes. Dalton Trans. 2021, 50, 11909–11917. 10.1039/D1DT01741A. 34374394

[ref11] Delaney A. R.; Yu L.-J.; Doan V.; Coote M. L.; Colebatch A. L. Bimetallic Nickel Complexes Supported by a Planar Macrocyclic Diphosphoranide Ligand. Chem.—Eur. J. 2023, 29, e202203940 10.1002/chem.202203940. 36545819

[ref12] Hall P. D.; Stevens M. A.; Wang J. Y.; Pham L. N.; Coote M. L.; Colebatch A. L. Copper and Zinc Complexes of 2,7-Bis(6-Methyl-2-Pyridyl)-1,8-Naphthyridine - A Redox-Active, Dinucleating Bis(Bipyridine) Ligand. *Inorg*. Chem. 2022, 61, 19333–19343. 10.1021/acs.inorgchem.2c03126. 36404623

[ref13] Desnoyer A. N.; Nicolay A.; Ríos P.; Ziegler M. S.; Tilley T. D. Bimetallics in a Nutshell: Complexes Supported by Chelating Naphthyridine-Based Ligands. Acc. Chem. Res. 2020, 53, 1944–1956. 10.1021/acs.accounts.0c00382. 32878429

[ref14] Ríos P.; See M. S.; Handford R. C.; Teat S. J.; Tilley T. D. Robust Dicopper(I) μ-Boryl Complexes Supported by a Dinucleating Naphthyridine-Based Ligand. Chem. Sci. 2022, 13, 6619–6625. 10.1039/D2SC00848C. 35756530 PMC9172574

[ref15] Nicolay A.; Héron J.; Shin C.; Kuramarohit S.; Ziegler M. S.; Balcells D.; Tilley T. D. Unsymmetrical Naphthyridine-Based Dicopper(I) Complexes: Synthesis, Stability, and Carbon-Hydrogen Bond Activations. Organometallics 2021, 40, 1866–1873. 10.1021/acs.organomet.1c00188.

[ref16] Nicolay A.; Tilley T. D. Selective Synthesis of a Series of Isostructural M^II^Cu^I^ Heterobimetallic Complexes Spontaneously Assembled by an Unsymmetrical Naphthyridine-Based Ligand. Chem.—Eur. J. 2018, 24, 10329–10333. 10.1002/chem.201802623. 29852541

[ref17] Lapointe S.; Khaskin E.; Fayzullin R. R.; Khusnutdinova J. R. Stable Nickel(I) Complexes with Electron-Rich, Sterically-Hindered, Innocent PNP Pincer Ligands. Organometallics 2019, 38, 1581–1594. 10.1021/acs.organomet.9b00026.

[ref18] See M. S.; Ríos P; Tilley T. D. Diborane Reductions of CO_2_ and CS_2_ Mediated by Dicopper μ-Boryl Complexes of a Robust Bis(Phosphino)-1,8-Naphthyridine Ligand. ChemRxiv (Inorganic Chemistry), Jan. 12, 2024 10.26434/chemrxiv-2024-w2f4s. PMC11134609 38817536

[ref19] Bryant R. G. The NMR time scale. J. Chem. Educ. 1983, 60, 933 10.1021/ed060p933.

[ref20] Ziegler M. S.; Levine D. S.; Lakshmi K. V.; Tilley T. D. Aryl Group Transfer from Tetraarylborato Anions to an Electrophilic Dicopper(I) Center and Mixed-Valence μ-Aryl Dicopper(I,II) Complexes. J. Am. Chem. Soc. 2016, 138, 6484–6491. 10.1021/jacs.6b00802. 27176131

[ref21] Davenport T. C.; Tilley T. D. Dinucleating Naphthyridine-Based Ligand for Assembly of Bridged Dicopper(I) Centers: Three-Center Two-Electron Bonding Involving an Acetonitrile Donor. Angew. Chem., Int. Ed. 2011, 50, 12205–12208. 10.1002/anie.201106081. 22038762

[ref22] Kounalis E.; Lutz M.; Broere D. L. Tuning the Bonding of a μ-Mesityl Ligand on Dicopper(I) through a Proton-Responsive Expanded PNNP Pincer Ligand. Organometallics 2020, 39, 585–592. 10.1021/acs.organomet.9b00829.

[ref23] Ríos P.; See M. S.; Handford R. C.; Cooper J. K.; Tilley T. D. Tetracopper σ bound μ-acetylide and diyne Units Stabilized by a Naphthyridine based Dinucleating Ligand. Angew. Chem., Int. Ed. 2023, 62, e202310307 10.1002/anie.202310307. 37705304

[ref24] Ziegler M. S.; Torquato N. A.; Levine D. S.; Nicolay A.; Celik H.; Tilley T. D. Dicopper Alkyl Complexes: Synthesis, Structure, and Unexpected Persistence. Organometallics 2018, 37, 2807–2823. 10.1021/acs.organomet.8b00443.

[ref25] Gunanathan C.; Milstein D. Metal-Ligand Cooperation by Aromatization-Dearomatization: A New Paradigm in Bond Activation and “Green” Catalysis. Acc. Chem. Res. 2011, 44, 588–602. 10.1021/ar2000265. 21739968

[ref26] Wyss C. M.; Bitting J.; Bacsa J.; Gray T. G.; Sadighi J. P. Bonding and Reactivity of a Dicopper(I) μ-Boryl Cation. Organometallics 2016, 35, 71–74. 10.1021/acs.organomet.5b00961.

[ref27] Kleeberg C.; Borner C. Syntheses, Structures, and Reactivity of NHC Copper(I) Boryl Complexes: A Systematic Study. Organometallics 2018, 37, 4136–4146. 10.1021/acs.organomet.8b00672.

[ref28] Borner C.; Anders L.; Brandhorst K.; Kleeberg C. Elusive Phosphine Copper(I) Boryl Complexes: Synthesis, Structures, and Reactivity. Organometallics 2017, 36, 4687–4690. 10.1021/acs.organomet.7b00775.

[ref29] Cordero B.; Gómez V.; Platero-Prats A. E.; Revés M.; Echeverría J.; Cremades E.; Barragán F.; Álvarez S. Covalent Radii Revisited. Dalton Trans. 2008, 2832–2838. 10.1039/b801115j. 18478144

[ref30] Harisomayajula N. V. S.; Makovetskyi S.; Tsai Y.-C. Cuprophilic Interactions in and between Molecular Entities. Chem. - Eur. J. 2019, 25, 8936–8954. 10.1002/chem.201900332. 31124211

[ref31] Ziegler M. S.; Lakshmi K. V.; Tilley T. D. Dicopper Cu(I)Cu(I) and Cu(I)Cu(II) Complexes in Copper-Catalyzed Azide-Alkyne Cycloaddition. J. Am. Chem. Soc. 2017, 139, 5378–5386. 10.1021/jacs.6b13261. 28394586

[ref32] Falivene L.; Cao Z.; Petta A.; Serra L.; Poater A.; Oliva R.; Scarano V.; Cavallo L. Towards the Online Computer-Aided Design of Catalytic Pockets. Nat. Chem. 2019, 11, 872–879. 10.1038/s41557-019-0319-5. 31477851

[ref33] Drescher W.; Kleeberg C. Terminal versus Bridging Boryl Coordination in N-Heterocyclic Carbene Copper(I) Boryl Complexes: Syntheses, Structures, and Dynamic Behavior. Inorg. Chem. 2019, 58, 8215–8229. 10.1021/acs.inorgchem.9b01041. 31148446

[ref34] Rodriguez T. M.; Deegbey M.; Chen C.-H.; Jakubikova E.; Dempsey J. L. Isocyanide Ligands Promote Ligand-to-Metal Charge Transfer Excited States in a Rhenium(II) Complex. Inorg. Chem. 2023, 62, 6576–6585. 10.1021/acs.inorgchem.2c03193. 36652699

[ref35] Cotton F. A.; Zingales F. The Donor-Acceptor Properties of Isonitriles as Estimated by Infrared Study. J. Am. Chem. Soc. 1961, 83, 351–355. 10.1021/ja01463a022.

[ref36] Müller P.; Herbst-Irmer R.; Spek A. L.; Schneider T. R.; Sawaya M. R. Crystal Structure Refinement: A Crystallographer’s Guide to SHELXL; Oxford Science Publications, 2006.

[ref37] Laitar D. S.; Müller P.; Sadighi J. P. Efficient Homogeneous Catalysis in the Reduction of CO_2_ to CO. J. Am. Chem. Soc. 2005, 127, 17196–17197. 10.1021/ja0566679. 16332062

[ref38] Horsley Downie T. M.; Charman R. S. C.; Hall J. W.; Mahon M. F.; Lowe J. P.; Liptrot D. J. A Stable Ring-Expanded NHC-Supported Copper Boryl and Its Reactivity Towards Heterocumulenes. Dalton Trans. 2021, 50, 16336–16342. 10.1039/D1DT03540A. 34734620

[ref39] Zhao H.; Lin Z.; Marder T. B. Density Functional Theory Studies on the Mechanism of the Reduction of CO_2_ to CO Catalyzed by Copper(I) Boryl Complexes. J. Am. Chem. Soc. 2006, 128, 15637–15643. 10.1021/ja063671r. 17147372

[ref40] Li Z.; Mayer R. J.; Ofial A. R.; Mayr H. From Carbodiimides to Carbon Dioxide: Quantification of the Electrophilic Reactivities of Heteroallenes. J. Am. Chem. Soc. 2020, 142, 8383–8402. 10.1021/jacs.0c01960. 32338511

[ref41] Haack P.; Limberg C.; Tietz T.; Metzinger R. Unprecedented Binding and Activation of CS_2_ in a Dinuclear Copper(I) Complex. Chem. Commun. 2011, 47, 6374–6376. 10.1039/c1cc11518a. 21547328

[ref42] Baenziger N. C.; Duax W. L. Crystal Structure and Molecular Motion of Solid Carbon Disulfide. J. Chem. Phys. 1968, 48, 2974–2981. 10.1063/1.1669561.

[ref43] Yang L.; Powell D. R.; Houser R. P. Structural Variation in Copper(I) Complexes with Pyridylmethylamide Ligands: Structural Analysis with a New Four-coordinate Geometry Index, τ_4_. Dalton Trans. 2007, 955–964. 10.1039/B617136B. 17308676

[ref44] Desnoyer A. N.; Nicolay A.; Ziegler M. S.; Lakshmi K. V.; Cundari T. R.; Tilley T. D. A Dicopper Nitrenoid by Oxidation of a CuICuI Core: Synthesis, Electronic Structure, and Reactivity. J. Am. Chem. Soc. 2021, 143, 7135–7143. 10.1021/jacs.1c02235. 33877827

