# Supplementary material for: Diborane Reductions of CO2 and CS2 Mediated by Dicopper μ-Boryl Complexes of a Robust Bis(phosphino)-1,8-naphthyridine Ligand
Source: Organometallics. 2024 May 3;43(10):1180–9. doi: 10.1021/acs.organomet.4c00122 (PMC11134609; doi:10.1021/acs.organomet.4c00122)
Supplement: Supplementary file 1 — om4c00122_si_001.pdf [file om4c00122_si_001.pdf]

Supporting Information

Diborane Reductions of CO_2 and CS_2 Mediated by Dicopper μ-Boryl Complexes of a Robust Bis(Phosphino)-1,8-Naphthyridine Ligand

Matthew S. See,[a,b] Pablo Ríos,[b,c] and T. Don Tilley*[a,b]

[a]Department of Chemistry, University of California, Berkeley, Berkeley, California 94720, United States
[b]Chemical Sciences Division, Lawrence Berkeley National Laboratory, Berkeley, California 94720, United States
[c]Instituto de Investigaciones Químicas (IIQ), Departamento de Química Inorgánica, Centro de Innovación en Química Avanzada (ORFEO-CINQA), CSIC and Universidad de Sevilla, Sevilla, 41092, Spain

*Corresponding author: tdtilley@berkeley.edu

(1) General Considerations. 2
(2) Experimental Section 3
(3) NMR and IR Spectra. 18
(4) X-ray Crystallography. 45
(5) % Buried Volume Calculations. 58
(6) Computational Details. 61
(7) Selected Natural Localized Molecular Orbitals 61
(8) References. 63

General Considerations

General methods: All manipulations were carried out using standard Schlenk techniques or in inert atmosphere gloveboxes filled with dry nitrogen. Solvents were stored over activated molecular sieves (3 Å) after having been collected from a JC Meyers Phoenix solvent purification system or after distillation after drying over potassium. Benzene-d_6, THF-d_8, and acetonitrile-d_3 were degassed with three freeze-pump-thaw cycles and stored over activated molecular sieves (3 Å) for at least 48 h prior to use.

Reagents: The ligand precursor 2,7-dichloro-1,8-naphthyridine, $[Cu(NCMe)_4][NTf_2]$, and lithium phenylacetylide were prepared according to the literature procedures.[1-3] Fluorene was purchased from commercial suppliers and heated to 120 °C at 60 mTorr for at least 24 h prior to use. Phenylacetylene and carbon disulfide were purchased from commercial suppliers, degassed with three freeze-pump-thaw cycles, and stored over activated molecular sieves (3 Å). Carbon dioxide (99.998%) was purchased from Linde Gas & Equipment Inc. and used as received. All other reagents were purchased from commercial suppliers and used as received.

NMR Spectroscopy: NMR spectra were collected at the UC Berkeley College of Chemistry NMR facilities on Bruker Avance 400, 500, and 600 MHz spectrometers and spectra were referenced to solvent residual signals (^{1}H, ^{13}C{^{1}H}),[4] or externally referenced (^{11}B{^{1}H}, ^{19}F{^{1}H}, ^{31}P{^{1}H}). Unless otherwise indicated, all NMR spectra were reported at 292 K. All NMR spectra were analyzed with the MestReNova software package.

IR Spectroscopy: Infrared spectra were collected on a Thermo Scientific Nicolet iS10 FTIR spectrometer as KBr pellets. Abbreviations for IR spectroscopy are as follows: s, sharp; w, weak.

Microanalysis: Analyses were conducted by Dr. Elena Kreimer at the UC Berkeley College of Chemistry Microanalytical Faculty. Elemental analysis was performed with a Perkin Elmer CHNS 2400 Series II analyzer.

<u>Experimental Section:</u>

Synthesis of 9-(diisopropylphosphaneyl)-fluorene

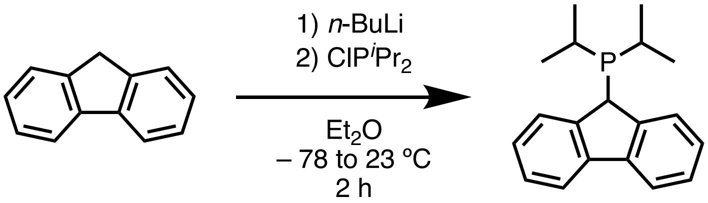

A Schlenk flask charged with a solution of fluorene (5.0 g, 30 mmol, 1.0 equiv.) in 80 mL of diethyl ether was cooled to 0 °C with an ice/water cold bath. Once cooled, a 1.8 M solution of *n*-BuLi in hexanes (19 mL, 33 mmol, 1.1 equiv.) was added portion-wise to the fluorene solution while stirring. The reaction mixture was warmed to 23 °C, and stirred for an additional 30 minutes, resulting in a vibrant orange solution. An additional Schlenk flask, charged with ClP^{i}Pr$_2$ (4.8 mL, 30 mmol, 1.0 equiv.) in 40 mL of diethyl ether, was cooled to –78 °C with a dry ice/acetone cold bath. The vibrant orange solution of the deprotonated fluorene was added portion-wise to the ClP^{i}Pr$_2$ solution and stirred for 15 minutes at –78 °C before warming to 23 °C and stirring for an additional 2 h. Upon completion of the reaction, the volatile materials were removed from the yellow suspension *in vacuo*, and the remaining solid was extracted with pentane (3 x 30 mL). The extracts were cannula filtered, combined in a Teflon-tapped Schlenk flask, and were left to crystallize for 16 h at –78 °C; after which, the mother liquor was decanted from the white crystals. The white crystals were washed with cold (–78 °C) pentane (3 x 20 mL), and all volatile material was removed *in vacuo* to yield an air-sensitive, white crystalline powder (7.1 g, 84% yield).

Spectroscopic data is in agreement with that previously reported.[5]

Synthesis of (diisopropylphosphaneyl)-fluorene-9-yl)lithium

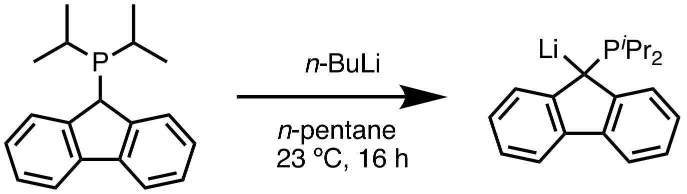

A 1.8 M solution of *n*-BuLi in hexanes (3.4 mL, 6.1 mmol, 1.1 equiv.) was added portion-wise to a solution of 9-(diisopropylphosphaneyl)-fluorene (1.5 g, 5.5 mmol, 1.0 equiv.) in 15 mL of pentane and the resulting mixture was stirred for 16 h to yield a yellow solid suspended in an orange solution. The orange supernatant was decanted and the remaining solid was washed with pentane (3 x 10 mL), and then all volatile material was removed *in vacuo* to yield a yellow powder (1.4 g, 86% yield).

Spectroscopic data is in agreement with that previously reported.[6]

Synthesis of 2,7-bis(9-(diisopropylphosphaneyl)-fluorene-9,9-diyl)-1,8-naphthyridine ($PNNP^{Flu}$)

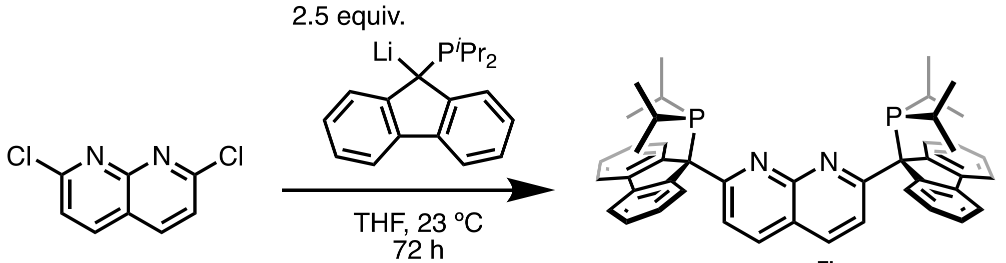

A 1.8 M solution of *n*-BuLi in hexanes (2.0 mL, 3.5 mmol, 2.5 equiv.) was added portion-wise to a solution of 9-(diisopropylphosphaneyl)-fluorene (1.0 g, 3.5 mmol, 2.5 equiv.) in 7 mL of THF and stirred for 1 h to yield an orange solution. Solid 2,7-dichloro-1,8-naphthyridine (0.28 g, 1.4 mmol, 1.0 equiv.) was added to the orange solution, which immediately resulted in a dark green suspension. The green suspension was stirred for 72 h before concentration *in vacuo* gave a blue foam that was subsequently triturated with pentane (20 mL). The pentane was decanted from the resulting blue solid and any remaining volatile material was removed *in vacuo* to yield a blue powder. The blue powder was extracted into toluene (3 x 10 mL) and the combined blue toluene extracts were filtered through a pad of celite supported on a fine fritted funnel. Concentration under vacuum gave a blue residue that was stirred vigorously in pentane (20 mL). The pentane was decanted, and all volatile material was removed *in vacuo* to yield a blue powder (0.70 g, 72% yield).

^{1}H NMR (400 MHz, benzene-d_6) δ 7.96 (d, J = 7.4 Hz, 4H, Flu CH), 7.64 (d, J = 7.2 Hz, 4H, Flu CH), 7.20 (m, 10H), 7.09 (d, J = 8.4 Hz, 2H, naph CH), 1.60 (septd, J = 7.3, 3.3 Hz, 4H, CH(CH$_3$)$_2$), 0.95 (dd, J = 14.8, 7.3 Hz, 12H, CH(CH_3)$_2$), 0.68 (dd, J = 10.5, 6.8 Hz, 12H, CH(CH_3)$_2$). **^{13}C{^{1}H} NMR** (125 MHz, benzene-d_6) δ 166.9 (d, $J_{C,P}$ = 12.0 Hz), 155.4, 148.2, 140.8, 136.6, 127.6 (d, $J_{C,P}$ = 17.7 Hz), 126.8 (d, $J_{C,P}$ = 4.0 Hz), 120.7 (d, $J_{C,P}$ = 4.7 Hz), 120.3, 119.8, 64.2 (d, $J_{C,P}$ = 31.1 Hz), 23.4 (d, $J_{C,P}$ = 24.4 Hz), 23.0 (d, $J_{C,P}$ = 27.2 Hz), 19.3, (d, $J_{C,P}$ = 11.7 Hz). **^{31}P{^{1}H} NMR** (162 MHz, benzene-d_6) δ 46.9. **HRMS (ESI)** calc'd for $[C_{46}H_{48}N_2P_2]^+$ $[M+H]^+$: *m/z* 691.3371, found 691.3436.

Synthesis of [(PNNP^{Flu})Cu_2($NCMe$)$_2$][NTf_2]$_2$ (1)

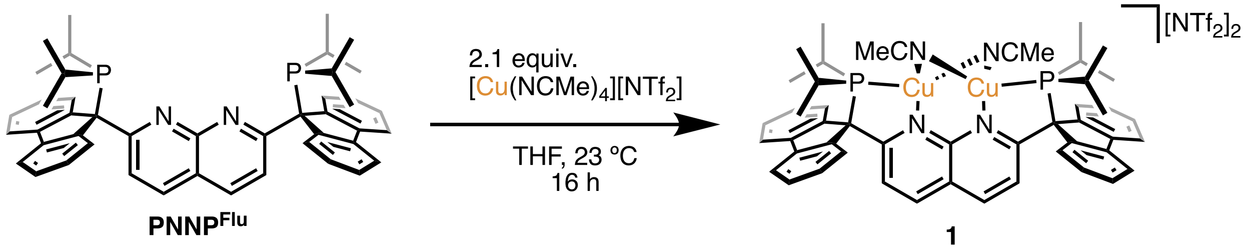

A solution of PNNP^{Flu} (0.25 g, 0.36 mmol, 1.0 equiv.) in 5 mL of THF was added to a stirred solution of [Cu(NCMe)$_4$][NTf_2] (0.39 g, 0.76 mmol, 2.1 equiv.) in 5 mL of THF. The reaction mixture immediately turned dark red and was stirred for an additional 16 h to yield a dark brown solution. The dark brown solution was filtered (glass microfiber in a Pasteur pipette) and concentrated *in vacuo*. The resulting sticky orange residue was washed with diethyl ether (3 x 10 mL) and placed under reduced pressure between each washing. The resulting orange foam was stirred vigorously in pentane (20 mL). The pentane was decanted from the orange solid, and all volatile material was removed *in vacuo* to yield a fine orange powder (0.46 g, 87% yield).

^{1}H NMR (500 MHz, acetonitrile-d_3) δ 8.37 (d, J = 8.7 Hz, 2H, naph CH), 8.05 (br, 2H, naph CH), 7.99 (d, J = 7.1 Hz, 4H, Flu CH), 7.97 (d, J = 6.7 Hz, 4H, Flu CH), 7.57 (t, J = 7.3 Hz, 4H, Flu CH), 7.49 (t, J = 7.3 Hz, 4H, Flu CH), 1.96 (s, 6H, NCCH_3), 1.80 (m, 4H, CH(CH$_3$)$_2$), 0.72 (dd, J = 16.4, 7.2 Hz, 12H, CH(CH_3)$_2$), 0.62 (dd, J = 13.9, 7.0 Hz, 12H, CH(CH_3)$_2$) . **^{13}C{^{1}H} NMR** (125 MHz, acetonitrile-d_3) δ 166.6, 144.6, 141.5, 140.0, 129.6, 128.7, 128.2, 122.2, 121.5, 119.7, 68.3, 64.4, 26.2, 24.3 (d, $J_{C,P}$ = 7.2 Hz), 20.9 (d, $J_{C,P}$ = 10.2 Hz), 18.7, (d, $J_{C,P}$ = 5.2 Hz), 1.8. **^{19}F{^{1}H} NMR** (470 MHz, acetonitrile-d_3) δ –80.2. **^{31}P{^{1}H} NMR** (202 MHz, acetonitrile-d_3) δ 44.0. **IR** (KBr, cm^{-1}): 2275 (w, $\nu_{C\equiv N}$).

Note: the persistent presence of unidentified minor products, as determined by multinuclear NMR spectroscopic techniques, prevented the isolation of analytically pure solid samples of this compound.

Synthesis of [(PNNP^{Flu})Cu_2(*μ*-Ph)][NTf_2] (2)

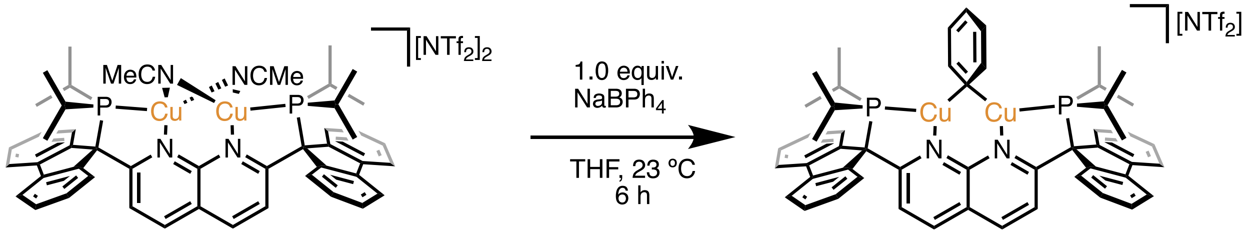

1 **2**

A solution of $NaBPh_4$ (12 mg, 0.035 mmol, 1.0 equiv.) in 1 mL of THF was added dropwise to a stirred solution of **1** (50 mg, 0.035 mmol, 1.0 equiv.) in 1 mL of THF. The reaction mixture immediately turned into a dark red solution. After 6 h, the reaction mixture was filtered (glass microfiber in a Pasteur pipette) and concentrated *in vacuo*. The resulting red residue was washed with pentane (2 x 2 mL) and diethyl ether (2 x 2 mL) and all volatile material was removed *in vacuo*. The resulting red residue was extracted into 2 mL of THF and this solution was layered under pentane (2 mL) in a 4 mL scintillation vial. The resulting bilayer was left to stand at 23 °C for 2 d. Afterwards, the mother liquor was decanted from the red crystals of sufficient quality for single crystal X-ray diffraction analysis. The red crystals were washed with pentane (4 mL), and the resulting mixture was decanted to give a red solid. All volatile material was removed *in vacuo* to yield an analytically pure red powder (33 mg, 82% yield).

^{1}H NMR (600 MHz, THF-d_8) δ 8.57 (d, J = 8.7 Hz, 2H, naph CH), 8.16 (d, J = 7.0 Hz, 2H, *o*-phenyl-C–H), 8.04 (d, J = 7.5 Hz, 4H, Flu CH), 7.86 (d, J = 7.7 Hz, 4H, Flu CH), 7.58 (t, J = 7.7 Hz, 4H, Flu CH), 7.51 (t, J = 7.1 Hz, 4H, Flu CH), 7.31 (d, J = 7.5 Hz, 2H, *m*-phenyl-C–H), 7.25 (d, J = 8.3 Hz, 2H, naph CH), 7.22 (d, J = 7.8 Hz, 1H, *p*-phenyl-C–H), 0.80 (dd, J = 17.9, 7.3 Hz, 12H, naph CH(CH_3)$_2$), 0.73 (dd, J = 14.1, 7.0 Hz, 12H, naph CH(CH_3)$_2$) Note: a resonance corresponding to the CH(CH_3)$_2$ group was not observable and is likely obscured by the THF-h_8/THF-d_8 solvent peaks between 1.70–1.80 ppm. **^{13}C{^{1}H} NMR** (151 MHz, THF-d_8) δ 169.4, 151.8, 145.2, 144.6, 143.8, 141.8, 130.3, 129.6, 129.5, 128.4, 126.6, 124.2, 122.3, 122.3, 120.3, 65.4 (d, $J_{C,P}$ = 6.4 Hz), 23.6 (d, $J_{C,P}$ = 7.2 Hz), 22.8 (d, $J_{C,P}$ = 11.2 Hz), 19.0 (d, $J_{C,P}$ = 3.4 Hz). **^{19}F{^{1}H} NMR** (565 MHz, THF-d_8) δ –81.6. **^{31}P{^{1}H} NMR** (243 MHz, THF-d_8) δ 49.1. **Anal. Calcd** for $C_{55}H_{57}Cu_2F_6N_3O_4P_2S_2$: C, 55.19; H, 4.55; N, 3.58. Found: C, 54.86; H, 4.30; N, 3.42.

Synthesis of [(PNNP^{Flu})Cu_2(μ-O^{t}Bu)][NTf_2] (3)

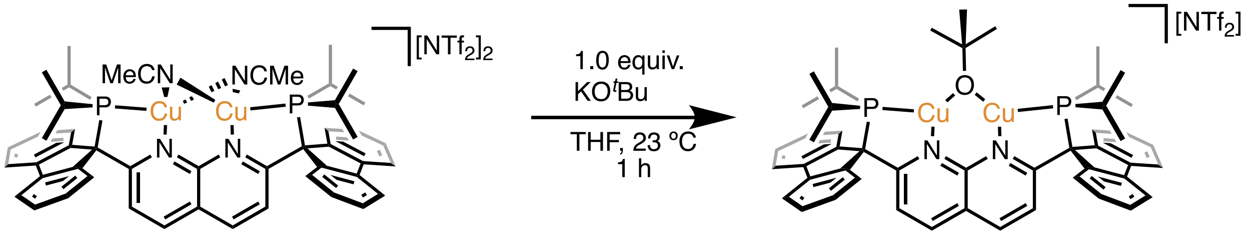

1 **3**

A solution of KO^{t}Bu (4.0 mg, 0.035 mmol, 1.0 equiv.) in 1 mL of THF was added dropwise to a stirring solution of **1** (50 mg, 0.035 mmol, 1.0 equiv.) in 1 mL of THF. The reaction mixture immediately turned into a dark green solution. After 1 h, the reaction mixture was filtered (glass microfiber in a Pasteur pipette) and concentrated *in vacuo*. The resulting green residue was washed with pentane (2 x 2 mL), and diethyl ether (2 x 2 mL) and any residual volatile materials were removed *in vacuo*. The green residue was extracted into THF (4 mL), and the resulting solution was filtered (glass microfiber in a Pasteur pipette) and layered under pentane (16 mL) in a 20 mL scintillation vial. The resulting bilayer was left to stand at 23 °C for 3 d. Afterwards, the mother liquor was decanted from the dichroic (orange with reflected light and green by transmitted light) crystals of sufficient quality for single crystal X-ray diffraction analysis. The crystals were washed with pentane (5 mL) and triturated with 20 mL of pentane. The pentane was decanted from the green solid. All volatile material was removed *in vacuo* to yield an analytically pure green powder (29 mg, 71% yield).

^{1}H NMR (500 MHz, THF-d_8) δ 8.40 (d, J = 8.5 Hz, 2H, naph CH), 8.07 (d, J = 7.4 Hz, 4H, Flu CH), 7.98 (d, J = 8.0 Hz, 4H, Flu CH), 7.60 (t, J = 7.6 Hz, 4H, Flu CH), 7.51 (t, J = 7.6 Hz, 4H, Flu CH), 7.15 (d, J = 8.6 Hz, 2H, naph CH), 1.86 (br s, 4H, CH(CH_3)$_2$), 1.69 (s, 9H, OC(CH_3)$_3$), 0.97 (dd, J = 17.7, 7.2 Hz, 12H, CH(CH_3)$_2$), 0.81 (br s, 12H, CH(CH_3)$_2$). **^{13}C{^{1}H} NMR** (125 MHz, THF-d_8) δ 167.9, 153.0, 144.9, 142.8, 142.0, 130.4, 129.5, 127.0, 122.3, 122.1, 120.1, 74.7, 64.2 (d, $J_{C,P}$ = 12.8 Hz), 37.0, 23.4 (d, $J_{C,P}$ = 14.6 Hz), 23.2 (d, $J_{C,P}$ = 8.6 Hz), 19.3. **^{19}F{^{1}H} NMR** (270 MHz, THF-d_8) δ – 79.8. **^{31}P{^{1}H} NMR** (202 MHz, THF-d_8) δ 54.2. **Anal. Calcd** for $C_{52}H_{57}Cu_2F_6N_3O_5P_2S_2$: C, 53.33; H, 4.91; N, 3.59. Found: C, 53.55; H, 4.69; N, 3.64.

Synthesis of [(PNNP^{Flu})Cu_2(*μ*-Bcat)][NTf_2] (4)

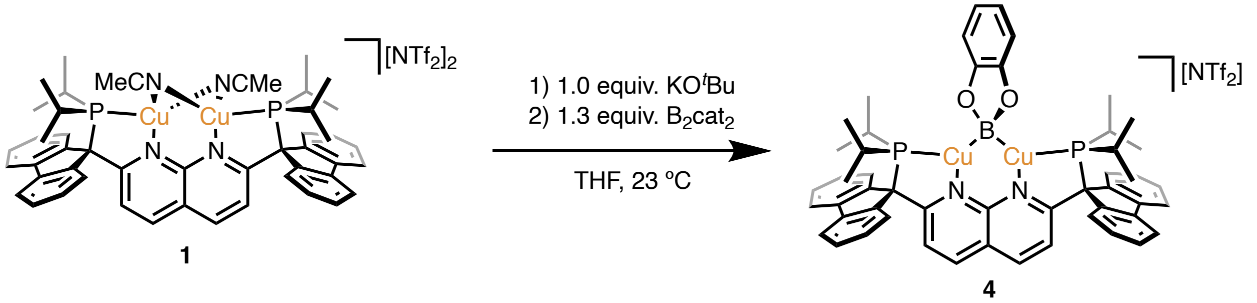

A solution of KO^{t}Bu (8.6 mg, 0.071 mmol, 1.0 equiv.) in 1 mL of THF was added dropwise to a stirred solution of **1** (100 mg, 0.071 mmol, 1.0 equiv.) in 1 mL of THF. The reaction mixture immediately turned dark green. After 1 h, a solution of B_2cat_2 (22 mg, 0.092 mmol, 1.3 equiv.) in 1 mL of THF was added dropwise to the green reaction mixture to yield a dark orange solution. After an additional 1 h, the dark orange reaction mixture was filtered (glass microfiber in a Pasteur pipette) and concentrated *in vacuo*. The resulting orange residue was washed with pentane (3 x 5 mL), and all volatile material was removed *in vacuo*. The orange residue was extracted into 1,2-difluorobenzene (3 x 1 mL), and the resulting solution was filtered (glass microfiber in a Pasteur pipette), the combined extracts were layered under pentane (12 mL) in a 20 mL scintillation vial. The resulting bilayer was left to stand at 23 °C for 2 d. Afterwards, the mother liquor was decanted from the orange crystals of sufficient quality for single crystal X-ray diffraction analysis. The crystals were washed with pentane (5 mL), and triturated with pentane (20 mL), which was decanted from the orange solid. All volatile material was removed *in vacuo* to yield an analytically pure orange powder (61 mg, 71% yield).

^{1}H NMR (500 MHz, THF-d_8) δ 8.66 (d, J = 8.4 Hz, 2H, naph C*H*), 8.07 (dd, J = 7.5 Hz, 4H, Flu C*H*), 7.71 (d, J = 7.7 Hz, 4H, Flu C*H*), 7.60 (t, J = 7.0 Hz, 4H, Flu C*H*), 7.55 (t, J = 7.5 Hz, 4H, Flu C*H*), 7.30 (d, J = 8.4 Hz, 2H, naph C*H*), 7.18 (dd, J = 5.8, 3.3 Hz, 2H, Bcat C*H*), 6.93 (dd, J = 5.9, 3.3 Hz, 2H, Bcat C*H*), 1.84 (m, 4H, C*H*(CH_3)$_2$), 0.89 (dd, J = 17.5, 7.3 Hz, 12H, CH(CH_3)$_2$), 0.77 (dd, J = 14.0, 7.1 Hz, 12H, CH(CH_3)$_2$). **^{11}B{^{1}H} NMR** (160 MHz, THF-d_8) δ 46.8. **^{13}C{^{1}H} NMR** (151 MHz, THF-H_8) δ 170.0, 150.0, 144.2, 143.4, 140.8, 129.6, 129.1, 125.6, 121.5, 121.4, 121.3, 111.2, 81.3, 53.8, 23.2 (t, $J_{C,P}$ = 4.4 Hz), 21.8 (t, $J_{C,P}$ = 5.5 Hz), 17.9. **^{19}F{^{1}H} NMR** (470 MHz, THF-d_8) δ –79.6. **^{31}P{^{1}H} NMR** (202 MHz, THF-d_8) δ 53.4. **Anal. Calcd** for $C_{54}H_{52}BCu_2F_6N_3O_6P_2S_2$: C, 53.30; H, 4.31; N, 3.45. Found: C, 53.41; H, 4.09; N, 3.77.

Synthesis of [(PNNP^{Flu})Cu_2(μ-Bpin)][NTf_2] (5)

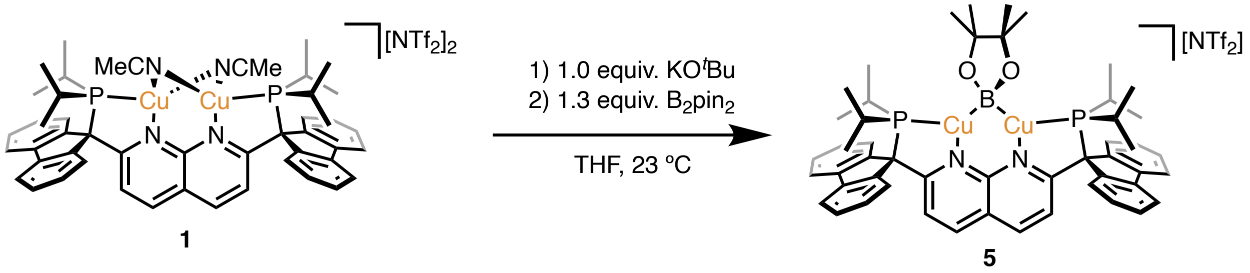

A solution of KO^{t}Bu (8.6 mg, 0.071 mmol, 1.0 equiv.) in 1 mL of THF was added dropwise to a stirred solution of **1** (100 mg, 0.071 mmol, 1.0 equiv.) in 1 mL of THF. The reaction mixture immediately turned dark green and after 1 h, a solution of B_2pin_2 (23 mg, 0.092 mmol, 1.3 equiv.) in 1 mL of THF was added dropwise to yield a dark orange solution. After an additional 6 h, the dark orange reaction mixture was filtered (glass microfiber in a Pasteur pipette) and concentrated *in vacuo*. The resulting orange residue was washed with pentane (3 x 5 mL), and all volatile material was removed *in vacuo*. The orange residue was extracted into 1,2-difluorobenzene (3 x 1 mL), and the resulting solution was filtered (glass microfiber in a Pasteur pipette), and the combined extracts were evacuated to dryness. The orange residue was extracted with dioxane (4 x 2 mL), and the dioxane extracts were combined and layered under pentane (12 mL) in a 20 mL scintillation vial. The resulting bilayer was left to stand at 23 °C for 1 d, and then the mother liquor was decanted from the orange crystals of sufficient quality for single crystal X-ray diffraction analysis. The crystals were washed with pentane (5 mL) and stirred vigorously in pentane (20 mL). The pentane was decanted from the orange solid. All volatile material was removed *in vacuo* to yield an analytically pure orange powder (79 mg, 91% yield).

^{1}H NMR (500 MHz, THF-d_8) δ 8.57 (d, J = 8.4 Hz, 2H, naph CH), 8.16 (d, J = 8.0 Hz, 4H, Flu CH), 7.67 (d, J = 7.3 Hz, 4H, Flu CH), 7.59 (t, J = 7.3 Hz, 4H, Flu CH), 7.52 (t, J = 7.2 Hz, 4H, Flu CH), 7.23 (d, J = 8.7 Hz, 2H, naph CH), 2.03 (sex, 4H, CH(CH_3)$_2$), 1.30 (s, 12H, Bpin CH_3), 0.96 (dd, J = 15.9, 6.9 Hz, 12H, CH(CH_3)$_2$), 0.88 (dd, J = 15.2, 7.0 Hz, 12H, CH(CH_3)$_2$). **^{11}B{^{1}H} NMR** (160 MHz, THF-d_8) δ 47.5. **^{13}C{^{1}H} NMR** (125 MHz, THF-d_8) δ 170.7, 144.6, 144.3, 141.8, 130.4, 129.8, 126.8, 122.5, 122.4, 121.9, 119.9, 82.2, 71.5, 24.6 (t, $J_{C,P}$ = 3.8 Hz), 22.1 (t, $J_{C,P}$ = 4.0 Hz), 19.4. **^{19}F{^{1}H} NMR** (470 MHz, THF-d_8) δ –80.2. **^{31}P{^{1}H} NMR** (202 MHz, THF-d_8) δ 51.2. **Anal. Calcd** for $C_{54}H_{60}BCu_2F_6N_3O_6P_2S_2$: C, 52.94; H, 4.94; N, 3.43. Found: C, 53.05; H, 4.85; N, 3.37.

Synthesis of [(PNNP^{Flu})Cu_2(μ-CCPh)][NTf_2] (6) from 1

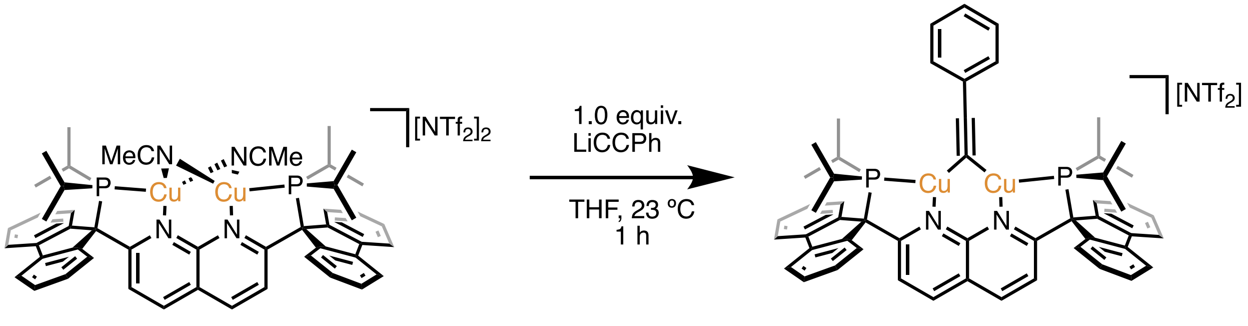

1 **6**

A solution of lithium phenylacetylide (4.6 mg, 0.042 mmol, 1.0 equiv.) in 1 mL of THF was added dropwise to a stirred solution of **1** (60 mg, 0.042 mmol, 1.0 equiv.) in 1 mL of THF. The reaction mixture immediately turned dark green and after 1 h, the reaction mixture was filtered (glass microfiber in a Pasteur pipette) and concentrated *in vacuo*. The resulting green residue was washed with pentane (2 x 2 mL), and diethyl ether (2 x 2 mL) and all volatile material was removed *in vacuo*. The green residue was extracted with THF (1 mL) and filtered into a 4 mL scintillation vial (glass microfiber in a Pasteur pipette). Vapor diffusion of pentane into the THF solution after 1 d at 23 °C resulted in dichroic (orange with reflected light and green by transmitted light) crystals of sufficient quality for single crystal X-ray diffraction analysis. Afterwards, the mother liquor was decanted from the crystals, and the crystals were washed with pentane (4 mL) and vigorously stirred in pentane (4 mL). The pentane was decanted from the orange solid. All volatile material was removed *in vacuo* to yield an analytically pure orange powder (33 mg, 67% yield).

^{1}H NMR (500 MHz, THF-d_8) δ 8.55 (d, J = 8.5 Hz, 2H, naph CH), 8.08 (d, J = 7.6 Hz, 4H, Flu CH), 7.90 (d, J = 7.6 Hz, 4H, Flu CH), 7.60 (t, J = 7.4 Hz, 4H, Flu CH), 7.55 (m, 3H, Ph CH), 7.52 (t, J = 7.4 Hz, 4H), 7.30 (d, J = 6.8 Hz, 2H, naph CH), 7.28 (d, J = 8.6 Hz, 2H, Ph CH), 1.91 (m, 4H, CH(CH_3)$_2$), 0.94 (dd, J = 17.6, 7.2 Hz, 12H, CH(CH_3)$_2$), 0.81 (dd, J = 14.0, 7.0 Hz, 12H, CH(CH_3)$_2$). **^{13}C{^{1}H} NMR** (125 MHz, THF-d_8) δ 169.6 (d, $J_{C,P}$ = 6.0 Hz), 144.7, 143.9, 141.9, 132.7, 130.4, 129.7, 129.4, 129.0, 126.9, 122.6, 122.3, 120.1, 65.4, 65.3 (d, $J_{C,P}$ = 7.2 Hz), 35.2, 23.7 (d, $J_{C,P}$ = 9.4 Hz), 23.4, 22.8 (d, $J_{C,P}$ = 10.2 Hz), 19.1, 14.5. **^{19}F{^{1}H} NMR** (470 MHz, THF-d_8) δ –79.8. **^{31}P{^{1}H} NMR** (202 MHz, THF-d_8) δ 51.0. **IR** (KBr, cm^{-1}): 1968 (w, $\nu_{C\equiv CPh}$). **Anal. Calcd** for $C_{56}H_{53}Cu_2F_6N_3O_4P_2S_2$: C, 56.09; H, 4.45; N, 3.50. Found: C, 56.47; H, 4.73; N, 3.63.

Synthesis of [(PNNP^{Flu})Cu_2(μ-CCPh)][NTf_2] (6) from 4

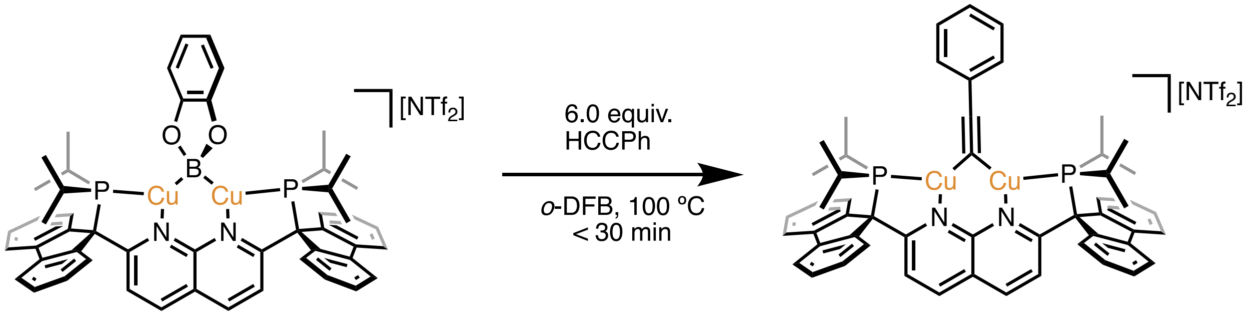

4 **6**

Phenylacetylene (10.8 uL, 0.099 mmol, 6.0 equiv.) was added by means of a microsyringe to a J. Young NMR tube that had been charged with complex **4** (20 mg, 0.016 mmol, 1.0 equiv.) in 0.5 mL of 1,2-difluorobenzene and an internal standard (1.0 µL of hexamethyldisiloxane). The NMR tube was placed in an oil bath at 100 °C for 0.5 h to quantitively yield **6** by ^{1}H NMR spectroscopy.

Spectroscopic data agrees with that of an independently synthesized sample (*vide supra*).

Complex **4** reacts with 6.0 equivalents of phenylacetylene at 100 °C to reach 100% conversion after 0.5 h while [(DPFN)Cu_2(μ-Bpin)][NTf_2] requires 24 h to reach 56% conversion.[7]

Synthesis of [(PNNP^{Flu})Cu_2(μ-CCPh)][NTf_2] (6) from 5

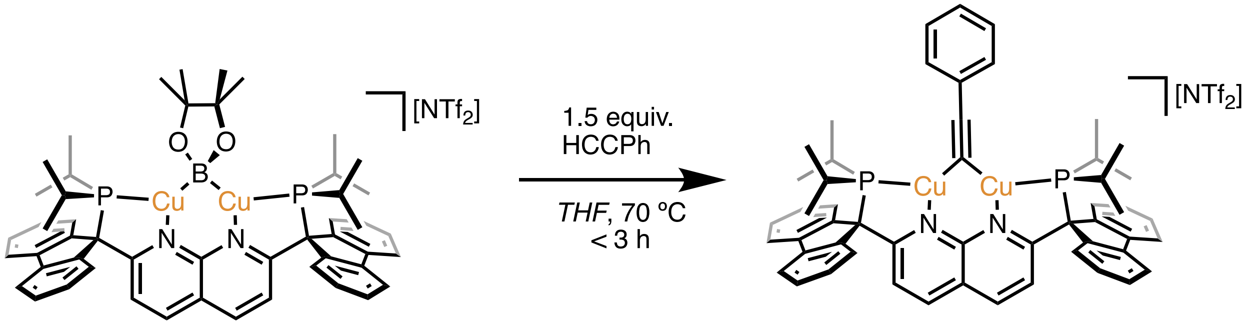

5 **6**

Phenylacetylene (2.7 uL, 0.025 mmol, 1.5 equiv.) was added by means of a microsyringe to a J. Young NMR tube that had been charged with complex **5** (20 mg, 0.016 mmol, 1.0 equiv.) in 0.5 mL of THF and an internal standard (1.0 µL of hexamethyldisiloxane). The NMR tube was placed in an oil bath at 70 °C for 3 h to quantitively yield **6** by ^{1}H NMR spectroscopy.

Spectroscopic data agrees with that of an independently synthesized sample (*vide supra*).

Complex **4** reacts with 1.5 equivalents of phenylacetylene at 70 °C to reach 100% conversion after 3 h while [(DPFN)Cu_2(μ-Bcat)][NTf_2] requires 22 h to reach 45% conversion.[7]

Synthesis of [(PNNP^{Flu})Cu$_2$(μ-Bcat)(CNXyl)][NTf$_2$] (7)

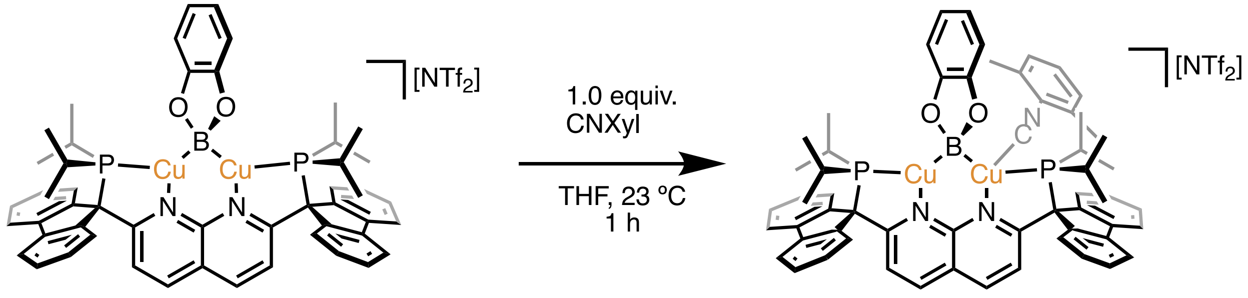

4 **7**

A solution of xylyl isocyanide (1.1 mg, 0.0082 mmol, 1.0 equiv.) in 0.2 mL of THF was added dropwise to a stirred solution of **4** (10 mg, 0.0082 mmol, 1.0 equiv.) in 0.2 mL of THF. The reaction mixture immediately turned red and after 1 h, the red reaction mixture was filtered (glass microfiber in a Pasteur pipette) and concentrated *in vacuo*. The red residue was washed with pentane (2 x 2 mL) and diethyl ether (2 x 2 mL) and all volatile material was removed *in vacuo*. The red residue was extracted into THF (1 mL) and the resulting solution was filtered into a 4 mL scintillation vial (glass microfiber in a Pasteur pipette). Vapor diffusion of pentane into the THF solution after 1 d at 23 °C resulted in dark red crystals of sufficient quality for single crystal X-ray diffraction analysis. Afterwards, the mother liquor was decanted from the crystals, and the crystals were washed with pentane (4 mL) and vigorously stirred in pentane (4 mL). The pentane was decanted from the red solid. All volatile material was removed *in vacuo* to yield an analytically pure red powder (11 mg, quant. yield).

^{1}H NMR (500 MHz, THF-d_8) δ 8.59 (d, J = 8.5 Hz, 2H, naph CH), 8.06 (d, J = 7.8 Hz, 4H, Flu CH), 7.81 (br s, 4H, Flu CH), 7.57 (br s, 4H, Flu CH), 7.40 (br s, 4H, Flu CH), 7.31 (br s, 3H, xylyl CH), 7.21 (d, J = 8.5 Hz, 2H, naph CH), 7.14 (dd, J = 5.8, 3.2 Hz, 2H, Bcat CH), 6.91 (dd, J = 5.9, 3.3 Hz, 2H, Bcat CH), 2.68 (br s, 6H, xylyl CH_3), 0.93 (br s, 12H, CH(CH_3)$_2$), 0.72 (br s, 12H, CH(CH_3)$_2$). *Note: a resonance corresponding to the CH(CH$_3$)$_2$ group was not observable.* **^{11}B{^{1}H} NMR** (160 MHz, THF-d_8) δ 48.4. **^{13}C{^{1}H} NMR** (125 MHz, THF-d_8) δ 149.7, 129.2, 128.4, 125.5, 121.5, 121.2, 121.0, 118.9, 110.8, 23.0, 21.1. *Note: a number of resonances were not observed due to the hypothesized molecular dynamic processes active on the NMR time scale.* **^{19}F{^{1}H} NMR** (470 MHz, THF-d_8) δ –79.8. **^{31}P{^{1}H} NMR** (202 MHz, THF-d_8) δ 52.6. **IR** (KBr, cm^{-1}): 2128 (s, $\nu_{C\equiv N}$). **Anal. Calcd** for $C_{63}H_{61}BCu_2F_6N_4O_6P_2S_2$: C, 56.13; H, 4.56; N, 4.16. Found: C, 55.78; H, 4.49; N, 4.25.

Synthesis of [(PNNP^{Flu})Cu$_2$(μ-OBcat)][NTf$_2$] (8)

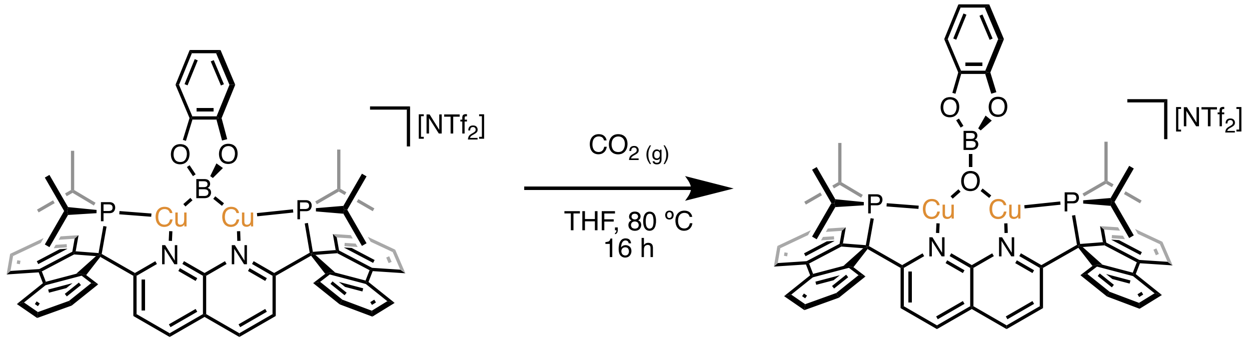

4 **8**

A J. Young NMR sample tube was charged with complex **4** (20 mg, 0.016 mmol, 1.0 equiv.) in 0.5 mL of THF and an internal standard (1.0 µL of hexamethyldisiloxane). Using a Schlenk line, the contents of the tube were degassed with four freeze-pump-thaw cycles. At 23 °C, CO_2 was introduced at 1 atm. The NMR tube was placed in an 80 °C oil bath for 16 h, and then the contents of the J. Young NMR tube were concentrated *in vacuo*. The orange residue was washed with pentane (2 x 2 mL) and diethyl ether (2 x 2 mL) and all volatile material was removed *in vacuo*. The resulting orange solid was extracted with 1,2-difluorobenzene (1 mL) and the resulting mixture was filtered into a 4 mL scintillation vial (glass microfiber in a Pasteur pipette). Vapor diffusion of pentane into the 1,2-difluorobenzene solution after 1 d at 23 °C resulted in orange crystals of sufficient quality for single crystal X-ray diffraction analysis. Afterwards, the mother liquor was decanted from the crystals, and the crystals were washed with pentane (4 mL) and vigorously stirred in pentane (4 mL). The pentane was decanted from the orange solid. All volatile material was removed *in vacuo* to yield an analytically pure orange powder (17 mg, 86% yield).

^{1}H NMR (400 MHz, THF-d_8) δ 8.46 (d, J = 8.5 Hz, 2H, naph C*H*), 8.07 (d, J = 7.6 Hz, 4H, Flu C*H*), 8.00 (d, J = 7.7 Hz, 4H, Flu C*H*), 7.61 (d, J = 7.4 Hz, 4H, Flu C*H*), 7.51 (t, J = 7.4 Hz, 4H, Flu C*H*), 7.28 (d, J = 8.5 Hz, 2H, naph C*H*), 7.01 (dd, J = 5.8, 3.4 Hz, 2H, Bcat C*H*), 6.93 (dd, J = 6.1, 3.4 Hz 2H, Bcat C*H*), 1.89 (sex, J = 7.4 Hz, 4H, C*H*(CH_3)$_2$), 1.04 (dd, J = 18.0, 7.4 Hz, 12H, CH(CH_3)$_2$), 0.84 (dd, J = 14.9, 7.0 Hz, 12H, CH(CH_3)$_2$). **^{11}B{^{1}H} NMR** (128 MHz, THF-d_8) δ 23.5. **^{13}C{^{1}H} NMR** (125 MHz, THF-d_8) δ 168.5 (d, $J_{C,P}$ = 3.7 Hz), 150.3, 144.7, 143.0, 142.0, 130.4, 129.6, 127.0, 122.5, 122.3, 122.2, 120.1, 112.1, 64.2, 23.6 (d, $J_{C,P}$ = 14.6 Hz), 23.1 (d, $J_{C,P}$ = 9.9 Hz), 19.0 (d, $J_{C,P}$ = 2.5 Hz). **^{19}F{^{1}H} NMR** (376 MHz, THF-d_8) δ –79.7. **^{31}P{^{1}H} NMR** (162 MHz, THF-d_8) δ 56.8. **Anal. Calcd** for $C_{54}H_{52}BCu_2F_6N_3O_7P_2S_2$: C, 52.60; H, 4.25; N, 3.41. Found: C, 52.96; H, 4.28; N, 3.29.

Synthesis of [(PNNP^{Flu})Cu_2(μ-OBpin)][NTf_2] (9)

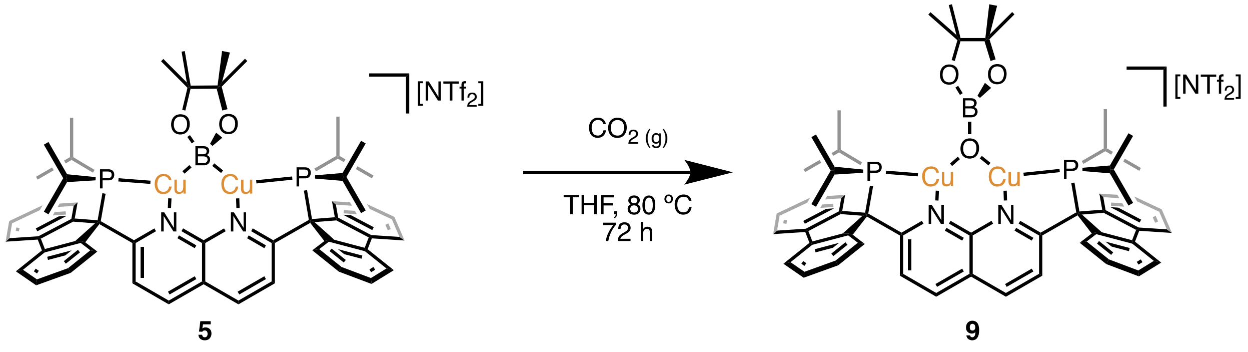

A J. Young NMR sample tube was charged with complex **5** (20 mg, 0.016 mmol, 1.0 equiv.) in 0.5 mL of THF and an internal standard (1.0 µL of hexamethyldisiloxane). Using a Schlenk line, the contents of the tube were degassed with four freeze-pump-thaw cycles. At 23 °C, CO_2 was introduced at 1 atm. The NMR tube was placed in an 80 °C oil bath for 16 h and then the contents of the NMR tube were concentrated *in vacuo*. The orange residue was washed with pentane (2 x 2 mL) and diethyl ether (2 x 2 mL) and all volatile material was removed *in vacuo*. The orange solid was extracted with THF (1 mL) and the resulting THF solution was filtered into a 4 mL scintillation vial (glass microfiber in a Pasteur pipette). Vapor diffusion of pentane into the THF solution after 1 d at 23 °C resulted in orange crystals of sufficient quality for single crystal X-ray diffraction analysis. Afterwards, the mother liquor was decanted from the crystals, and the crystals were washed with pentane (4 mL) and vigorously stirred in pentane (4 mL). The pentane was decanted from the orange powder. All volatile material was removed *in vacuo* to yield an analytically pure orange powder (18 mg, 92% yield).

^{1}H NMR (500 MHz, THFd_8) δ 8.40 (d, J = 8.5 Hz, 2H, naph CH), 8.06 (d, J = 7.2 Hz, 4H, Flu CH), 7.97 (d, J = 7.8 Hz, 4H, Flu CH), 7.59 (t, J = 7.5 Hz, 4H, Flu CH), 7.52 (t, J = 7.6 Hz, 4H, Flu CH), 7.24 (d, J = 8.5 Hz, 2H, naph CH) 1.85 (sex, J = 7.1 Hz, 4H, CH(CH_3)$_2$), 1.29 (s, 12H, Bpin CH_3), 0.99 (dd, J = 18.1, 7.2 Hz, 12H, CH(CH_3)$_2$), 0.81 (dd, J = 14.8, 6.9 Hz, 12H, CH(CH_3)$_2$). **^{11}B{^{1}H} NMR** (160 MHz, THF-d_8) δ 23.1. **^{13}C{^{1}H} NMR** (125 MHz, THF-d_8) δ 168.1, 144.7, 142.7, 141.9, 130.3, 129.6, 127.0, 122.2, 121.9, 83.5, 82.7, 82.1, 64.0, 23.5 (d, $J_{C,P}$ = 15.7 Hz), 23.1 (d, $J_{C,P}$ =10.6 Hz), 19.0. **^{19}F{^{1}H} NMR** (470 MHz, THF-d_8) δ –79.8. **^{31}P{^{1}H} NMR** (202 MHz, THF-d_8) δ 57.2. **Anal. Calcd** for $C_{54}H_{60}BCu_2F_6N_3O_7P_2S_2$: C, 52.26; H, 4.87; N, 3.39. Found: C, 52.06; H, 4.61; N, 3.41.

Synthesis of {[(PNNP^{Flu})Cu$_2$]$_2$[*μ*-S$_2$C(Bcat)$_2$]}[NTf$_2$]$_2$ (10)

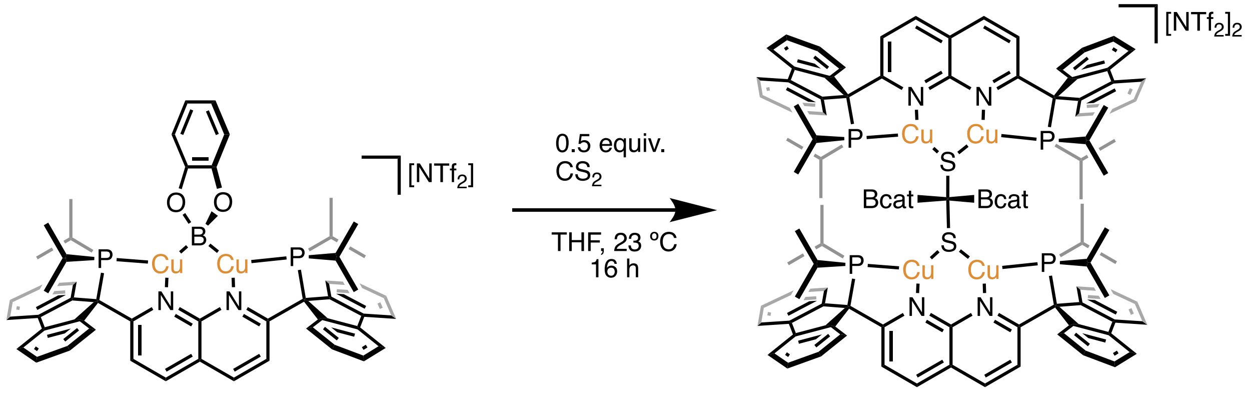

4 **10**

A solution of CS_2 (1.5 µL, 0.025 mmol, 0.5 equiv.) in 1 mL of THF was layered on top of a solution of **4** (60 mg, 0.049 mmol, 1.0 equiv.) in 1 mL of THF. The resulting bilayer solution was left to stand at 23 °C for 16 h, and then the mother liquor was decanted from the orange crystals of sufficient quality for single crystal X-ray diffraction analysis. The crystals were washed with pentane (4 mL) and stirred vigorously in pentane (4 mL). The pentane was decanted from the orange solid. All volatile material was removed *in vacuo* to yield an analytically pure orange powder (30 mg, 53% yield).

Anal. Calcd for $C_{97}H_{96}Cu_4F_{12}N_6O_8P_4S_6$: C, 51.27; H, 4.26; N, 3.70. Found: C, 51.58; H, 4.09; N, 3.37. *Note: Due to the complex's insolubility, characterization by NMR spectroscopy was not possible.*

Synthesis of [(PNNP^{Flu})Cu_2(μ,κ^2-S_2CBpin)][NTf_2] (11)

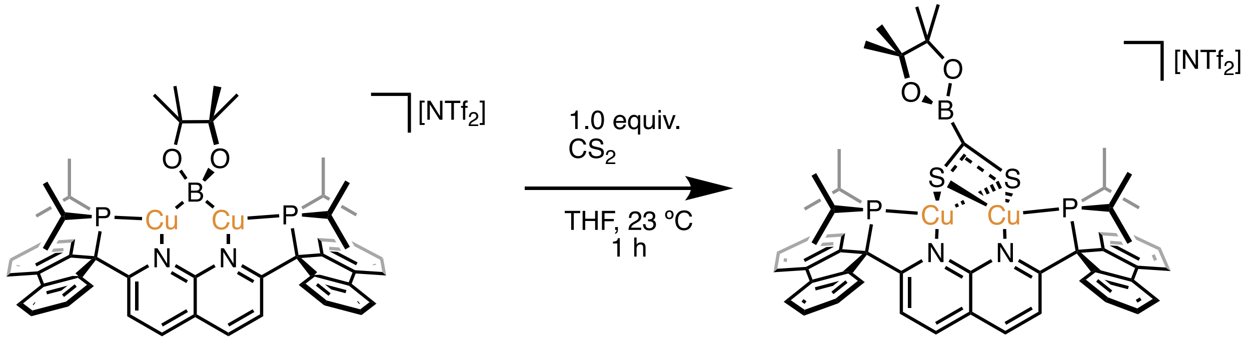

5 **11**

A solution of CS_2 (5.0 uL, 0.082 mmol, 1.0 equiv.) in 0.2 mL of THF was added dropwise to a stirring solution of **5** (100 mg, 0.082 mmol, 1.0 equiv.) in 1.0 mL of THF. The reaction mixture immediately turned red, and then the red reaction mixture was filtered (glass microfiber in a Pasteur pipette) and concentrated *in vacuo*. The red residue was washed with pentane (2 x 2 mL), and stirred vigorously in pentane (4 mL). The pentane was decanted from the red solid. All volatile material was removed *in vacuo* to yield an analytically pure red powder (80 mg, 75% yield).

Red crystals, suitable for single crystals X-ray diffraction analysis, were obtained from a diethyl ether/pentane vapor diffusion after 24 h at 23 °C.

^{1}H NMR (600 MHz, THF-*d*$_8$) δ 8.53 (d, J = 8.5 Hz, 2H, naph C*H*), 8.07 (d, J = 7.7 Hz, 4H, Flu C*H*), 7.88 (d, J = 7.8 Hz, 4H, Flu C*H*), 7.59 (t, J = 7.5 Hz, 4H, Flu C*H*), 7.47 (t, J = 7.6 Hz, 4H, Flu C*H*), 7.24 (d, J = 8.5 Hz, 2H, naph C*H*), 2.13 (sex, J = 6.9 Hz, 4H, C*H*(CH_3)$_2$), 1.29 (s, 12H, Bpin C*H*$_3$), 0.95 (dd, J = 16.4, 7.1 Hz, 12H, CH(C*H*$_3$)$_2$), 0.85 (dd, J = 15.5, 7.4 Hz, 12H, CH(C*H*$_3$)$_2$). **^{11}B{^{1}H} NMR** (128 MHz, THF-*d*$_8$) δ 25.3. **^{13}C{^{1}H} NMR** (151 MHz, THF-*d*$_8$) δ 171.2, 144.6, 144.4, 141.8, 130.4, 129.5, 127.0, 122.4, 122.3, 120.1, 86.6, 71.5, 25.0, 24.3 (d, $J_{C,P}$ = 8.2 Hz), 21.1 (d, $J_{C,P}$ = 7.3 Hz), 19.2 (d, $J_{C,P}$ = 4.3 Hz). **^{19}F{^{1}H} NMR** (565 MHz, THF-*d*$_8$) δ –80.1. **^{31}P{^{1}H} NMR** (243 MHz, THF-*d*$_8$) δ 44.8. **Anal. Calcd** for $C_{55}H_{60}BCu_2F_6N_3O_6P_2S_4$: C, 50.77; H, 4.65; N, 3.23. Found: C, 50.40; H, 4.59; N, 3.33

NMR and IR Spectra:

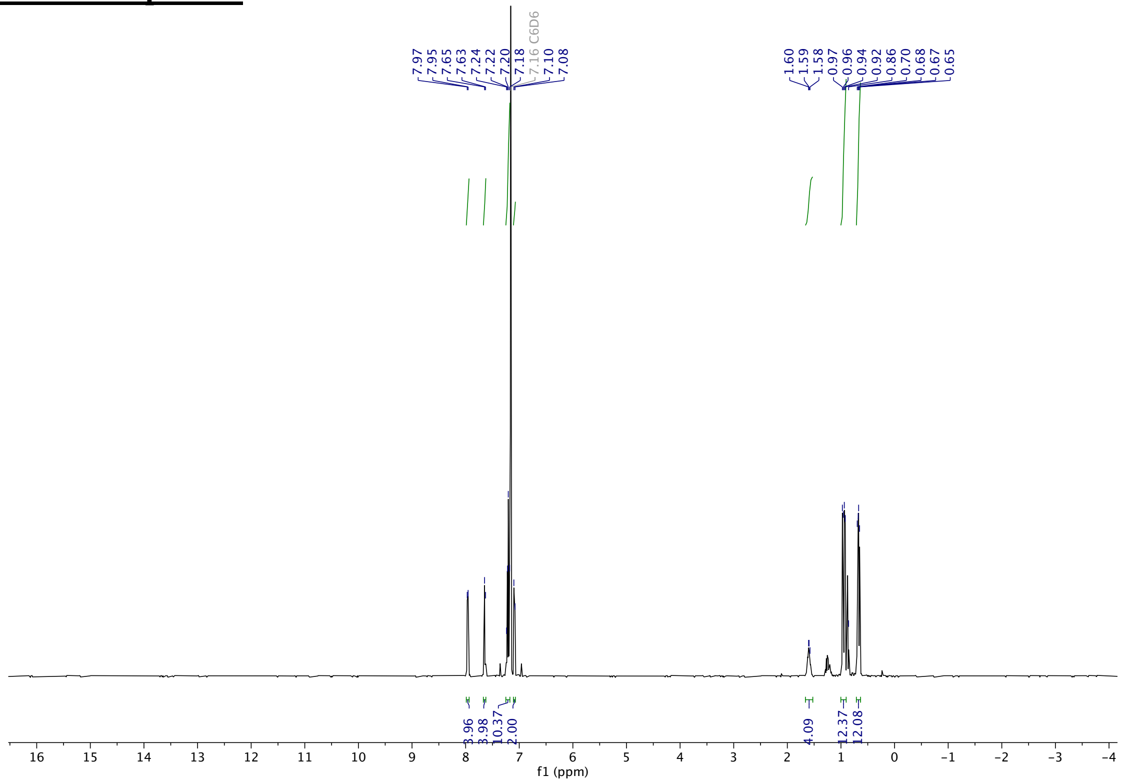

Figure S1. ^{1}H NMR spectrum (benzene-d_6, 500 MHz) of **PNNP^{Flu}**.

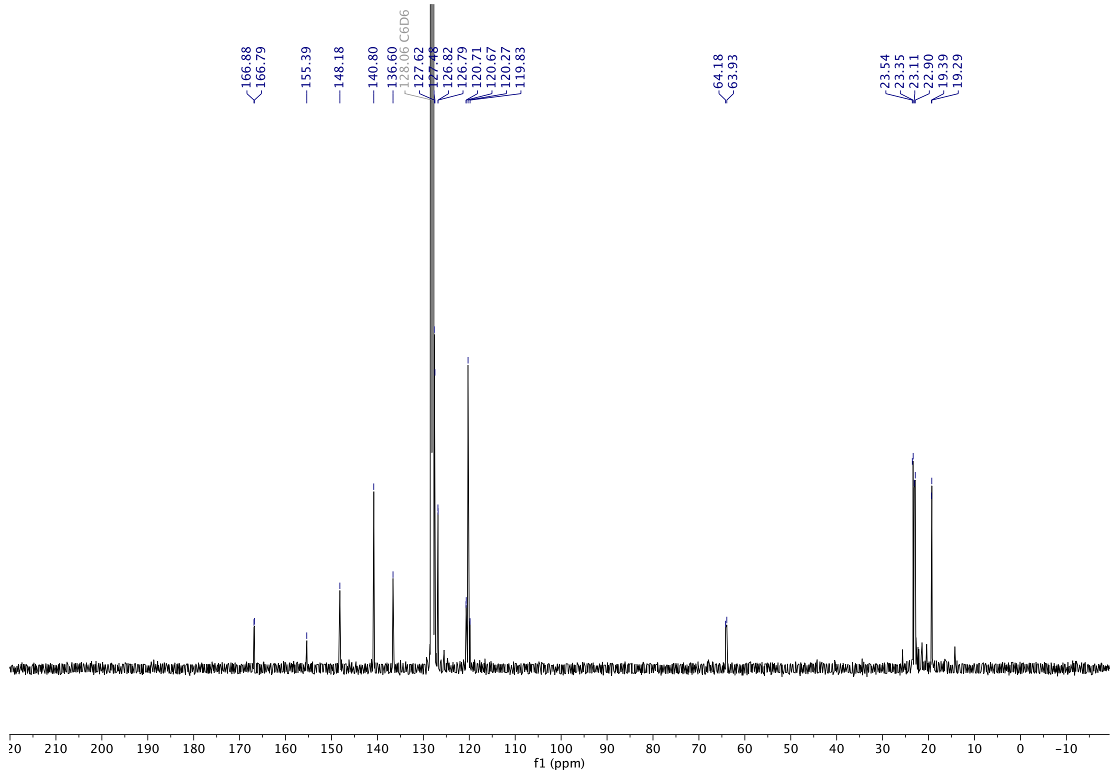

Figure S2. $^{13}C\{^{1}H\}$ NMR spectrum (benzene-d_6, 125 MHz) of **PNNP^{Flu}**.

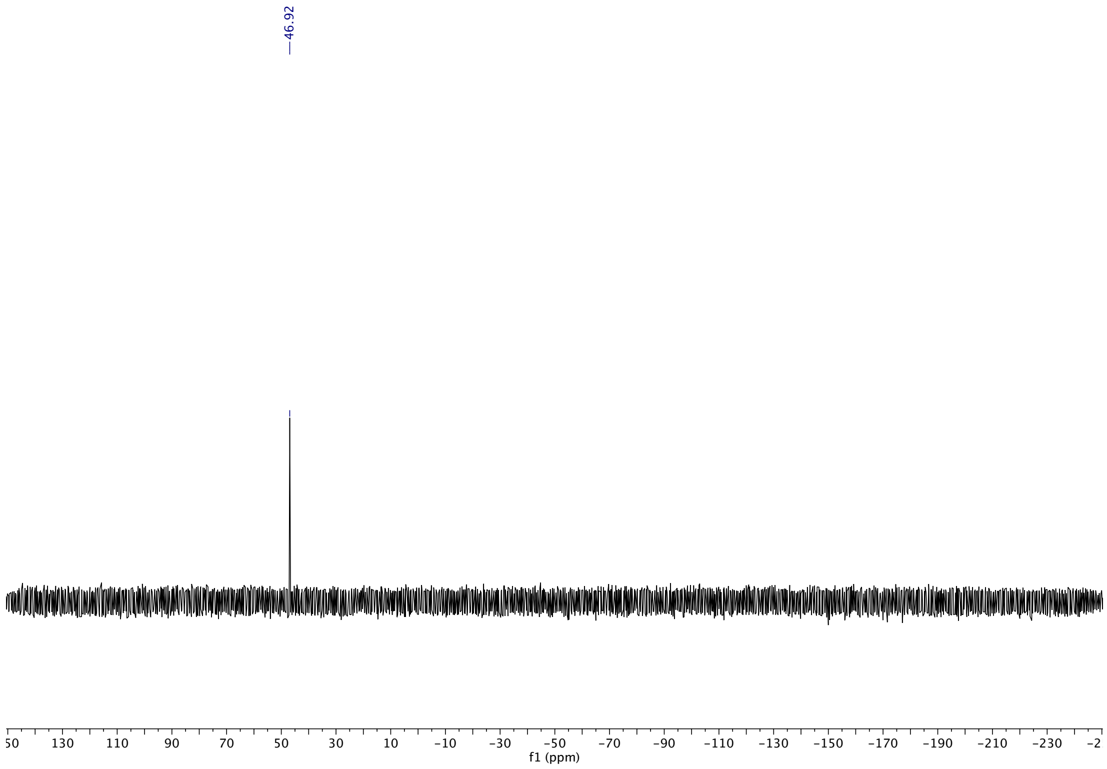

Figure S3. $^{31}P\{^{1}H\}$ NMR spectrum (benzene-d_6, 202 MHz) of Complex **PNNP^{Flu}**.

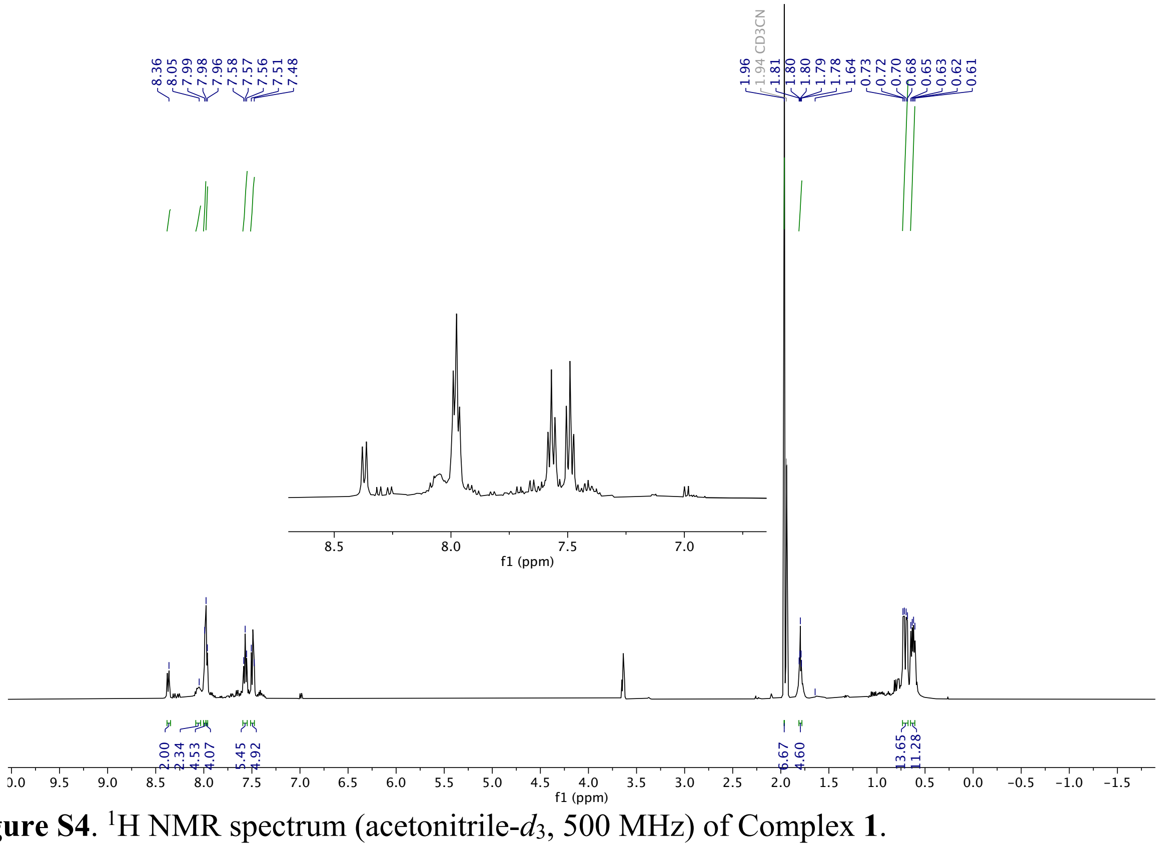

Figure S4. ^{1}H NMR spectrum (acetonitrile-d_3, 500 MHz) of Complex **1**.

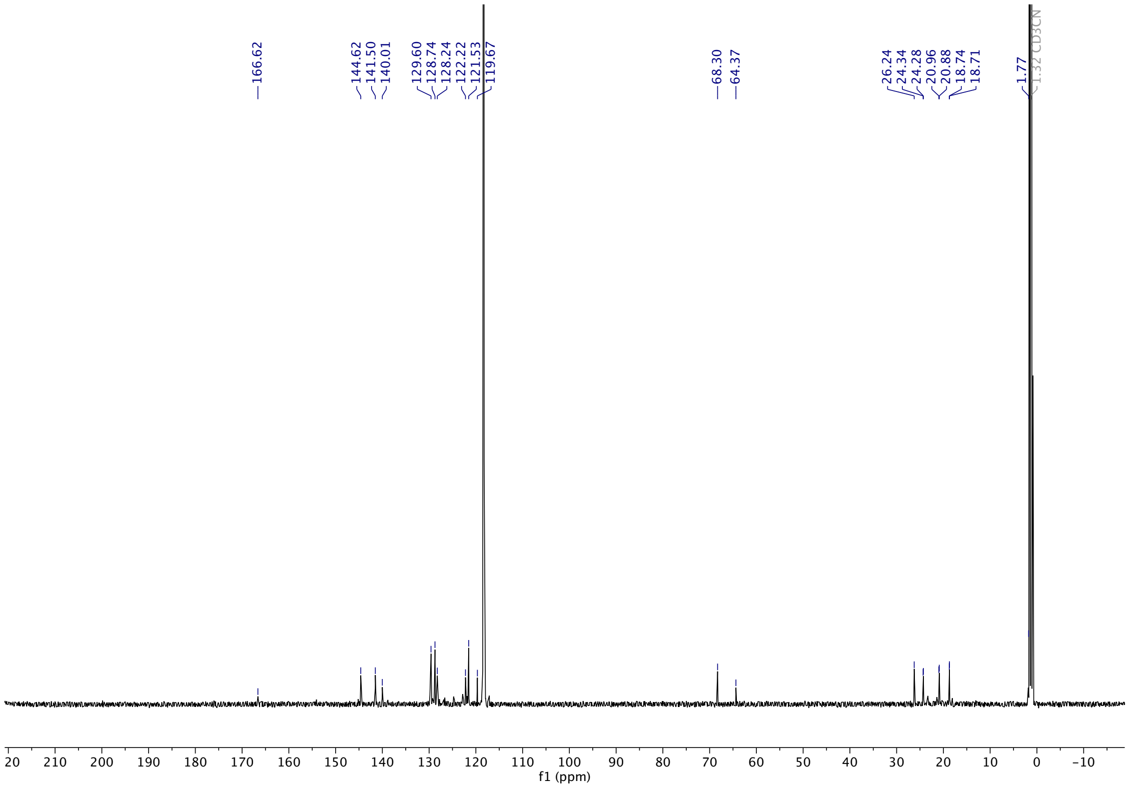

Figure S5. $^{13}C\{^1H\}$ NMR spectrum (acetonitrile-d_3, 125 MHz) of Complex **1**.

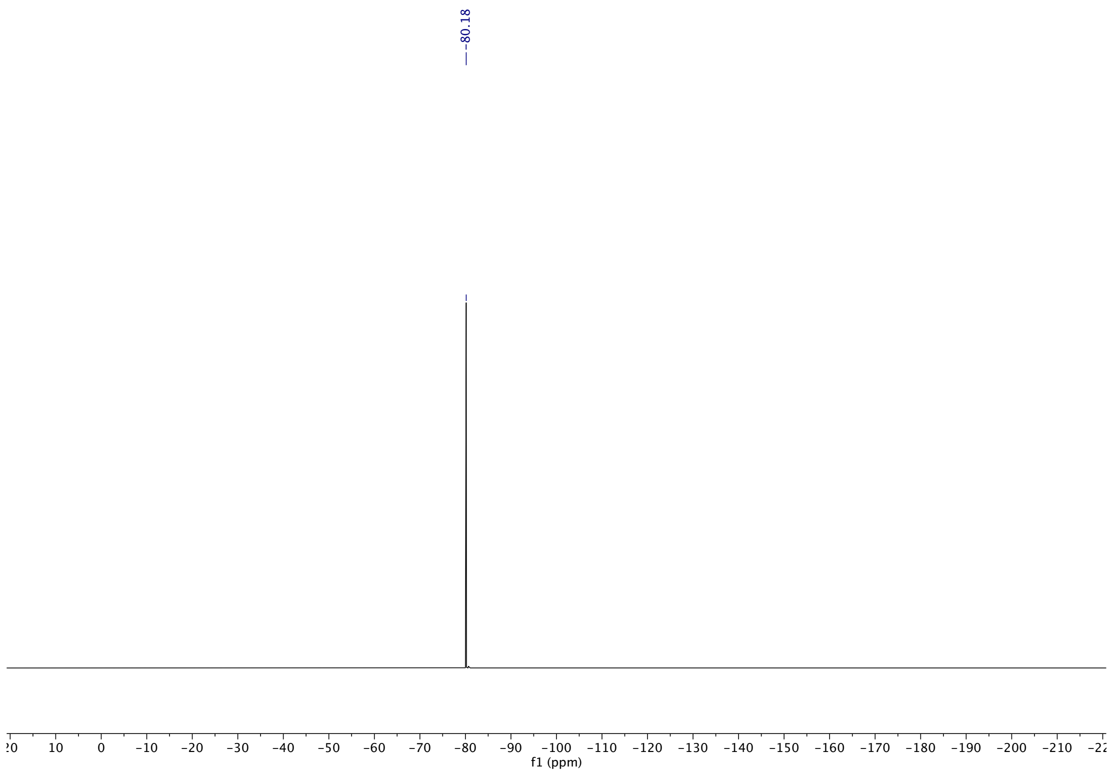

Figure S6. $^{19}F\{^1H\}$ NMR spectrum (acetonitrile-d_3, 470 MHz) of Complex **1**.

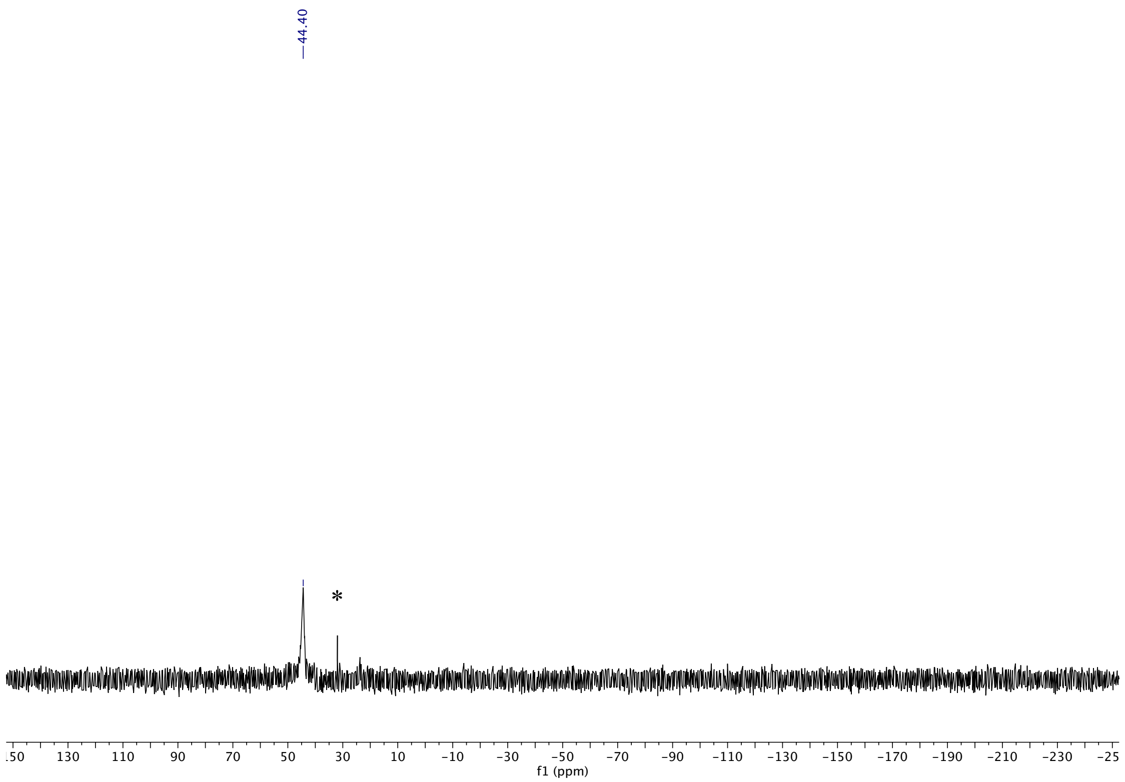

Figure S7. $^{31}P\{^{1}H\}$ NMR spectrum (acetonitrile-d_3, 202 MHz) of Complex **1**. *indicates an unidentified impurity.

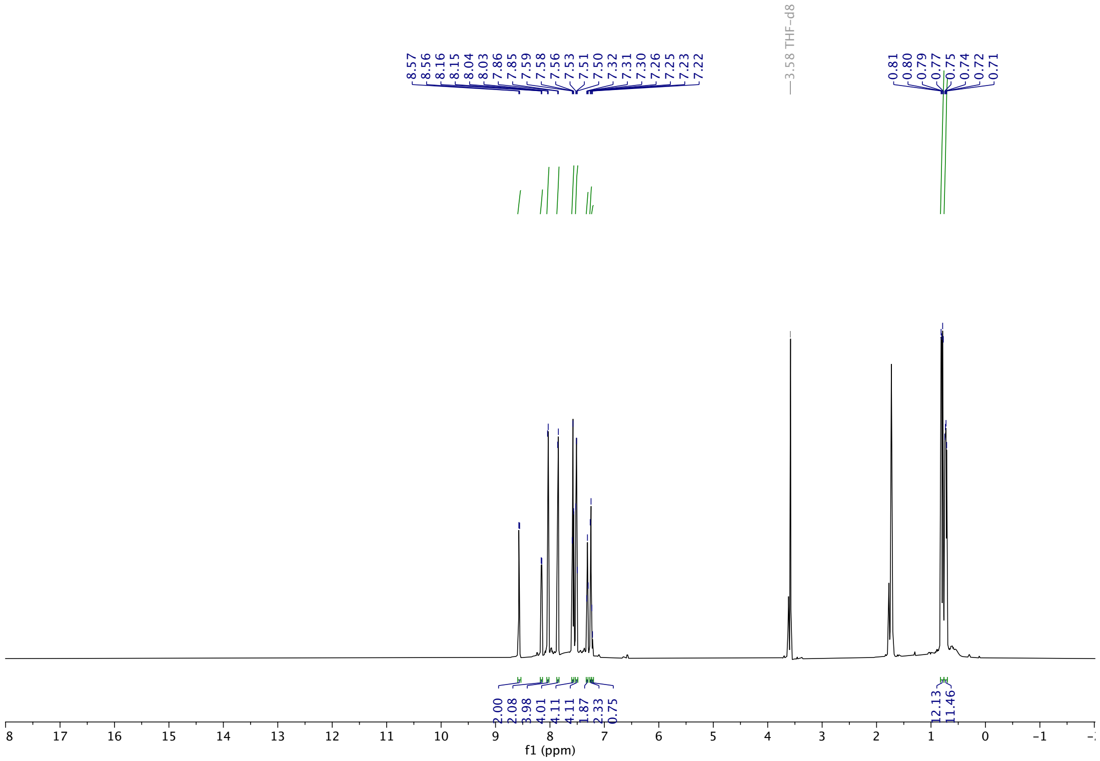

Figure S8. ^{1}H NMR spectrum (THF-d_8, 600 MHz) of Complex **2**.

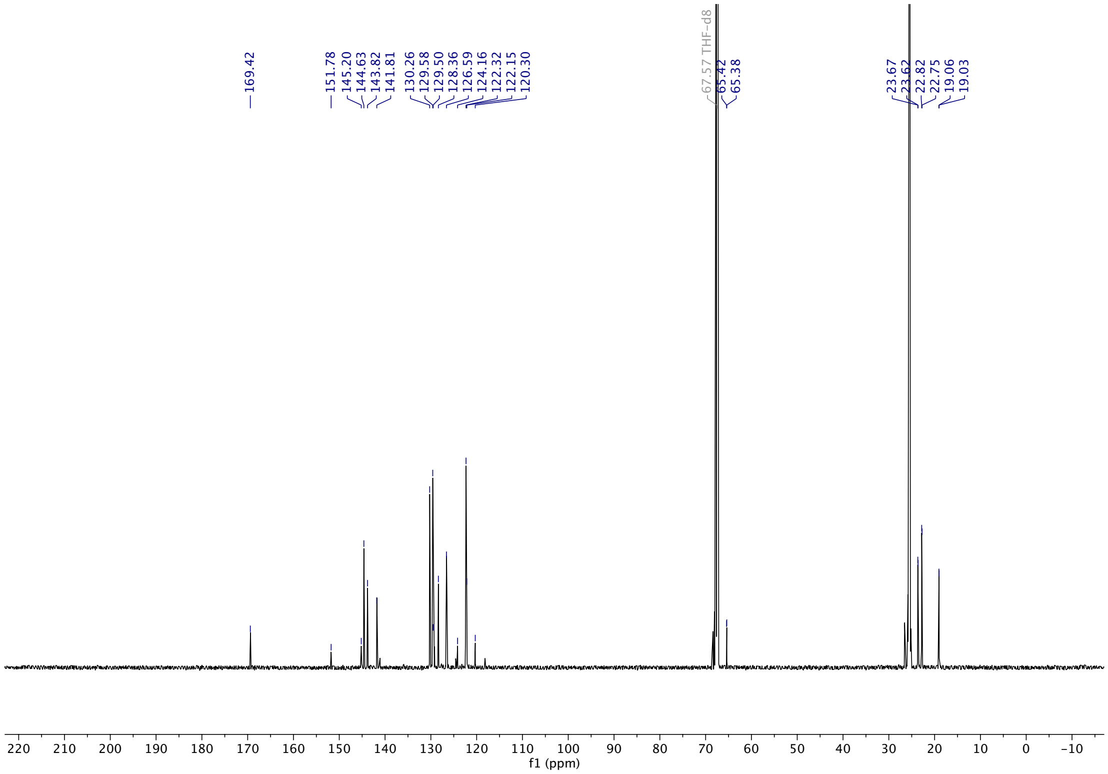

Figure S9. $^{13}C\{^{1}H\}$ NMR spectrum (THF-d_8, 151 MHz) of Complex **2**.

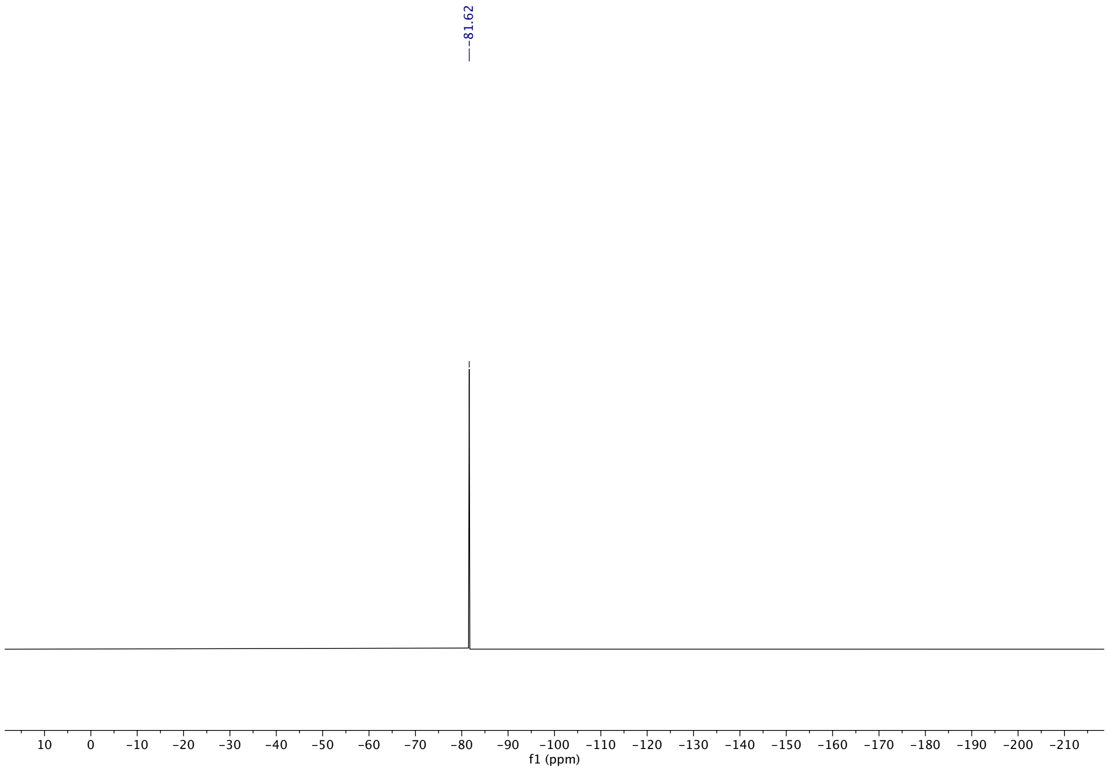

Figure S10. $^{19}F\{^{1}H\}$ NMR spectrum (THF-d_8, 565 MHz) of Complex **2**.

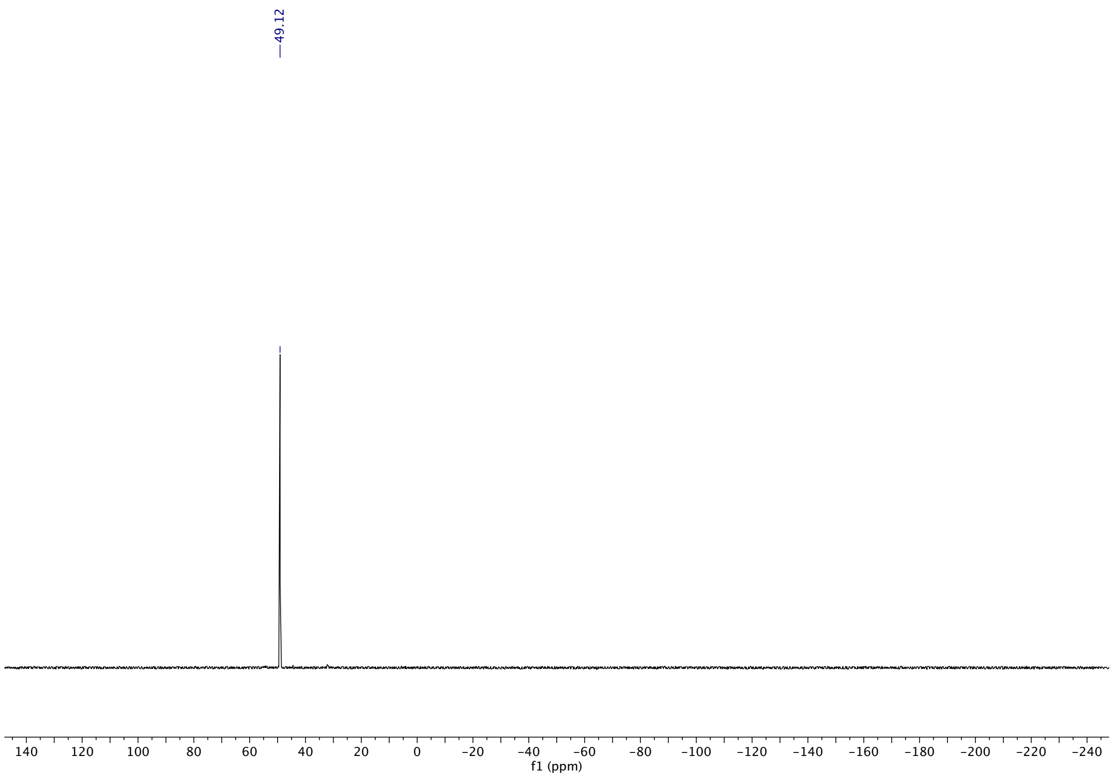

Figure S11. $^{31}P\{^{1}H\}$ NMR spectrum (THF-d_8, 243 MHz) of Complex **2**.

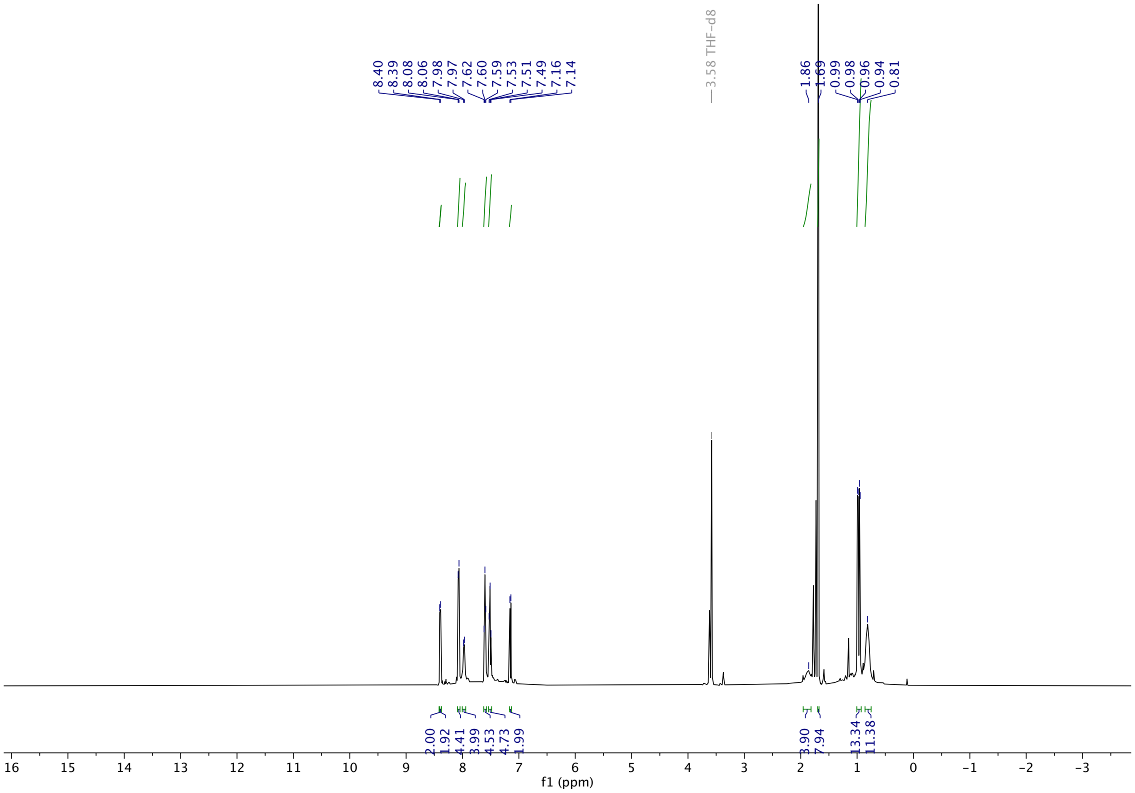

Figure S12. ^{1}H NMR spectrum (THF-d_8, 500 MHz) of Complex **3**.

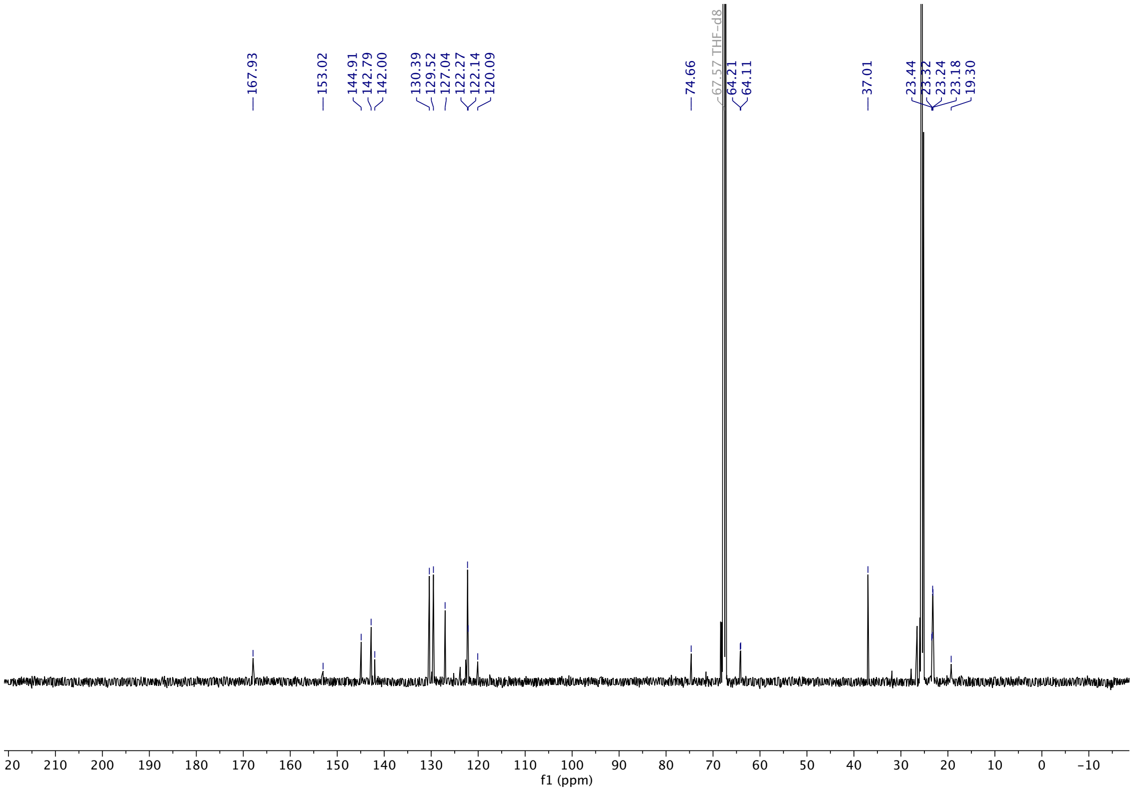

Figure S13. $^{13}C\{^{1}H\}$ NMR spectrum (THF-d_8, 125 MHz) of Complex **3**.

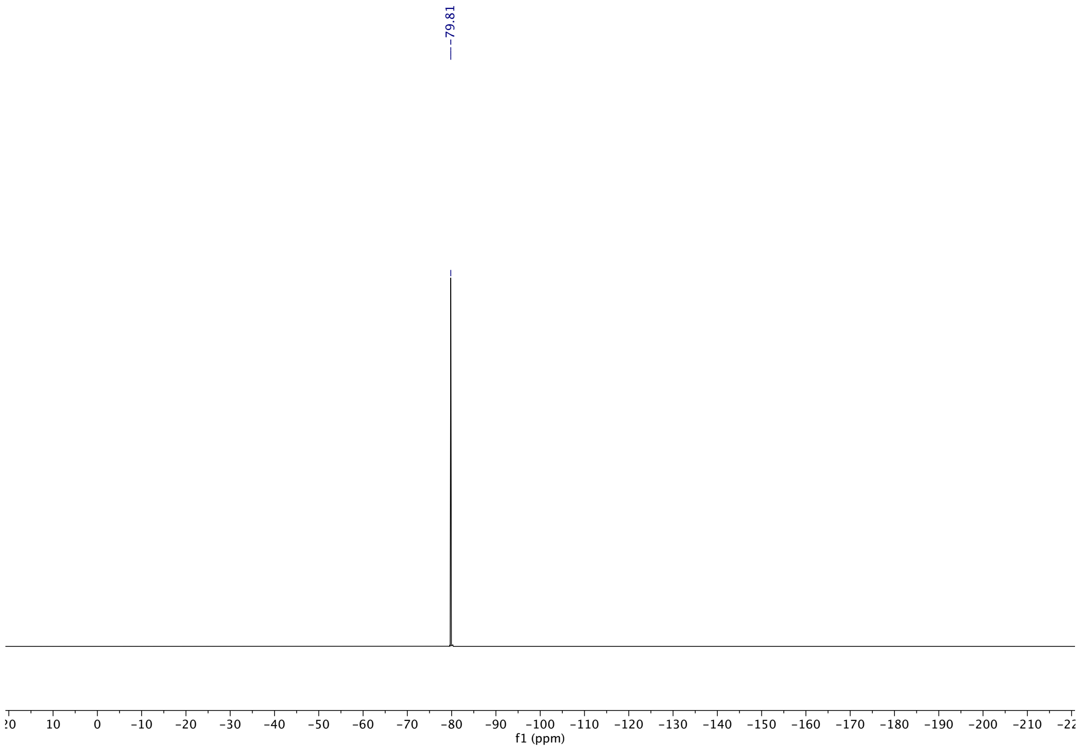

Figure S14. $^{19}F\{^{1}H\}$ NMR spectrum (THF-d_8, 470 MHz) of Complex **3**.

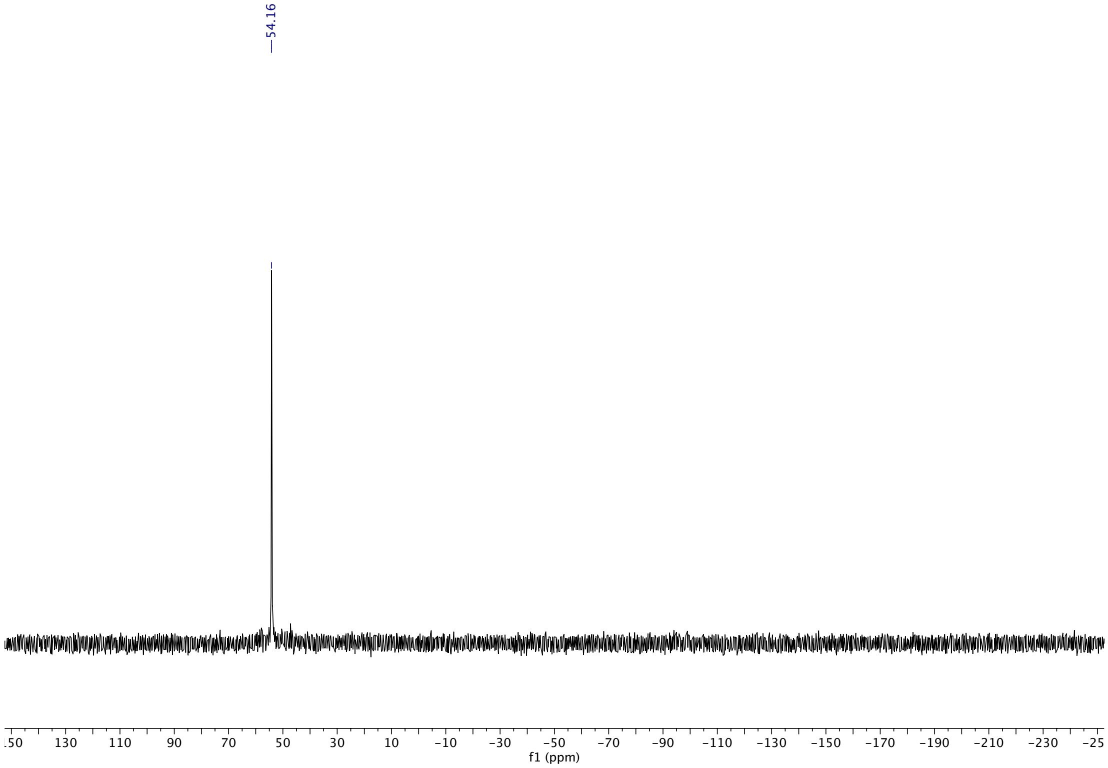

Figure S15. $^{31}P\{^1H\}$ NMR spectrum (THF-d_8, 202 MHz) of Complex **3**.

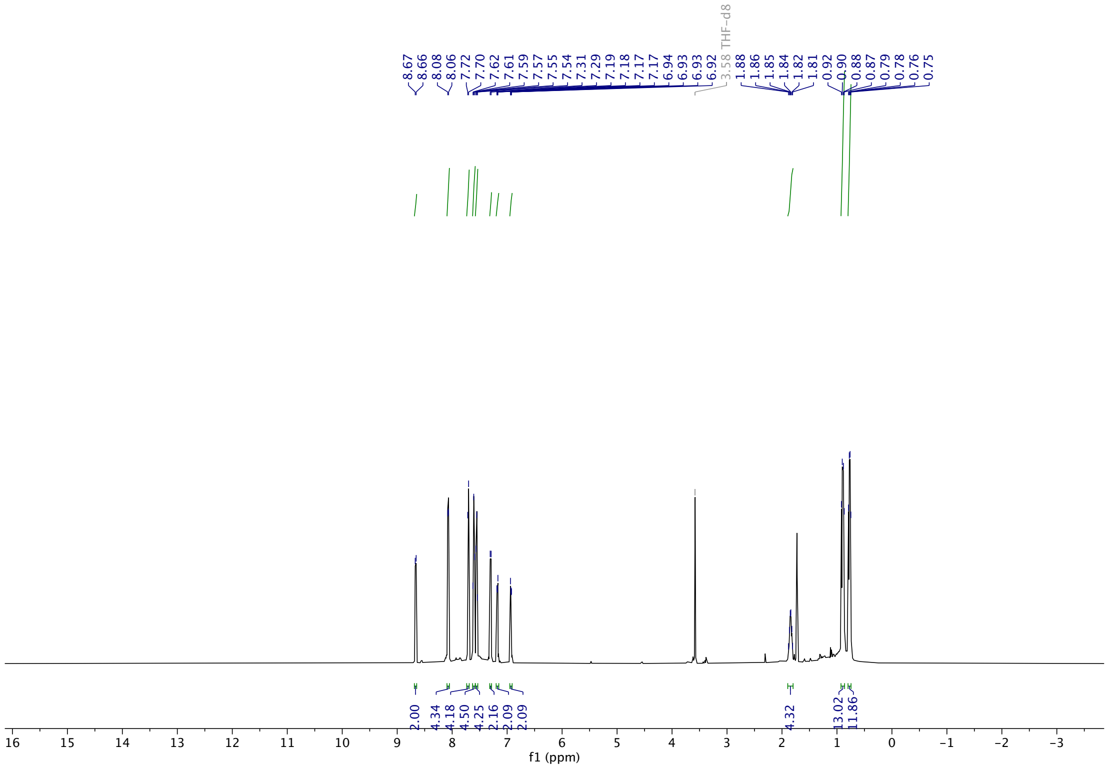

Figure S16. ^1H NMR spectrum (THF-d_8, 500 MHz) of Complex **4**.

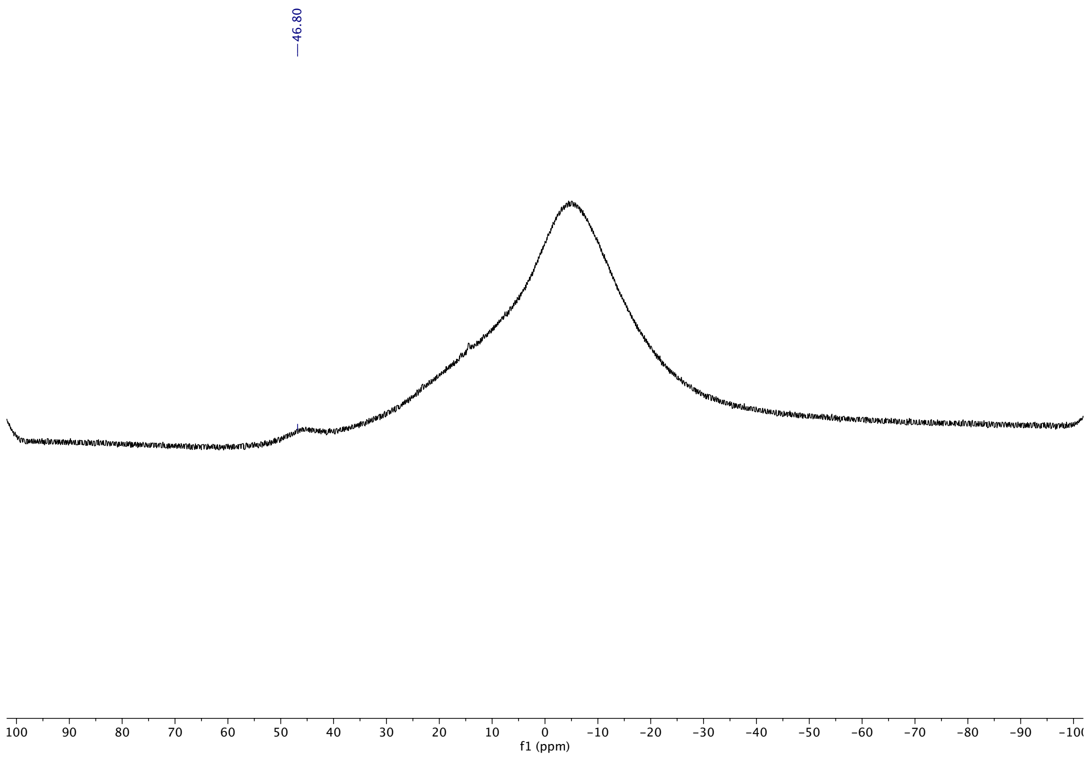

Figure S17. $^{11}B\{^{1}H\}$ NMR spectrum (THF-d_8, 160 MHz) of Complex **4**.

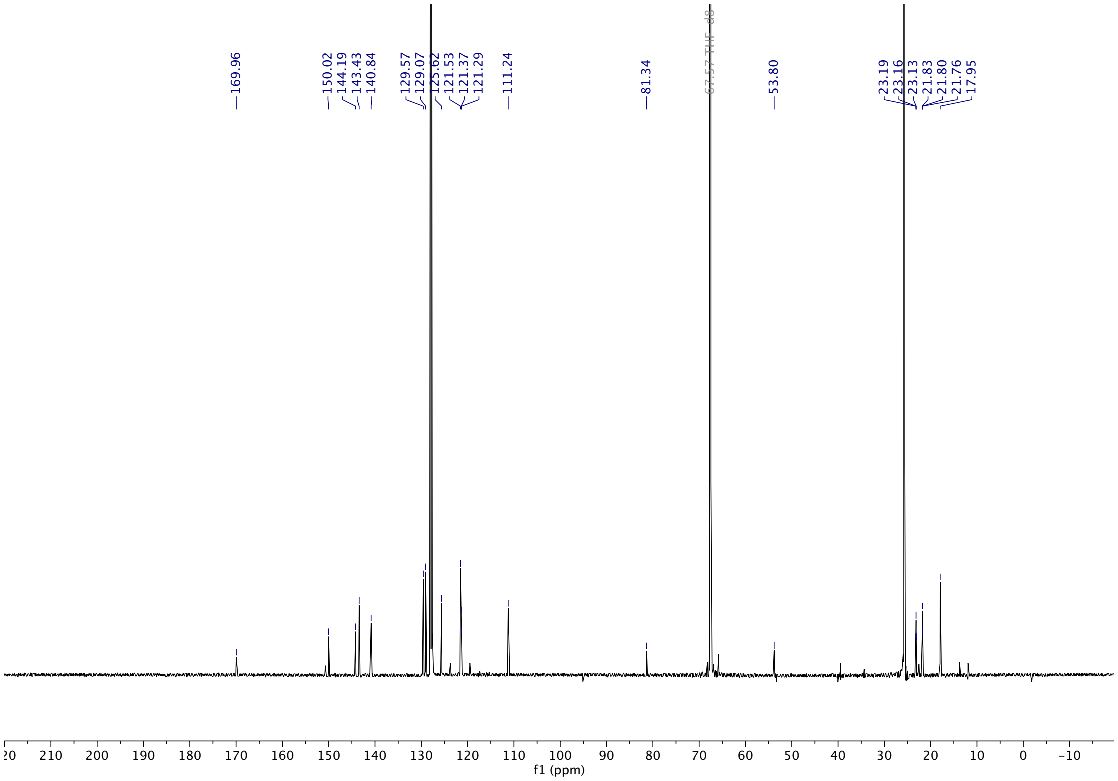

Figure S18. $^{13}C\{^{1}H\}$ NMR spectrum (THF-d_8, 125 MHz) of Complex **4**.

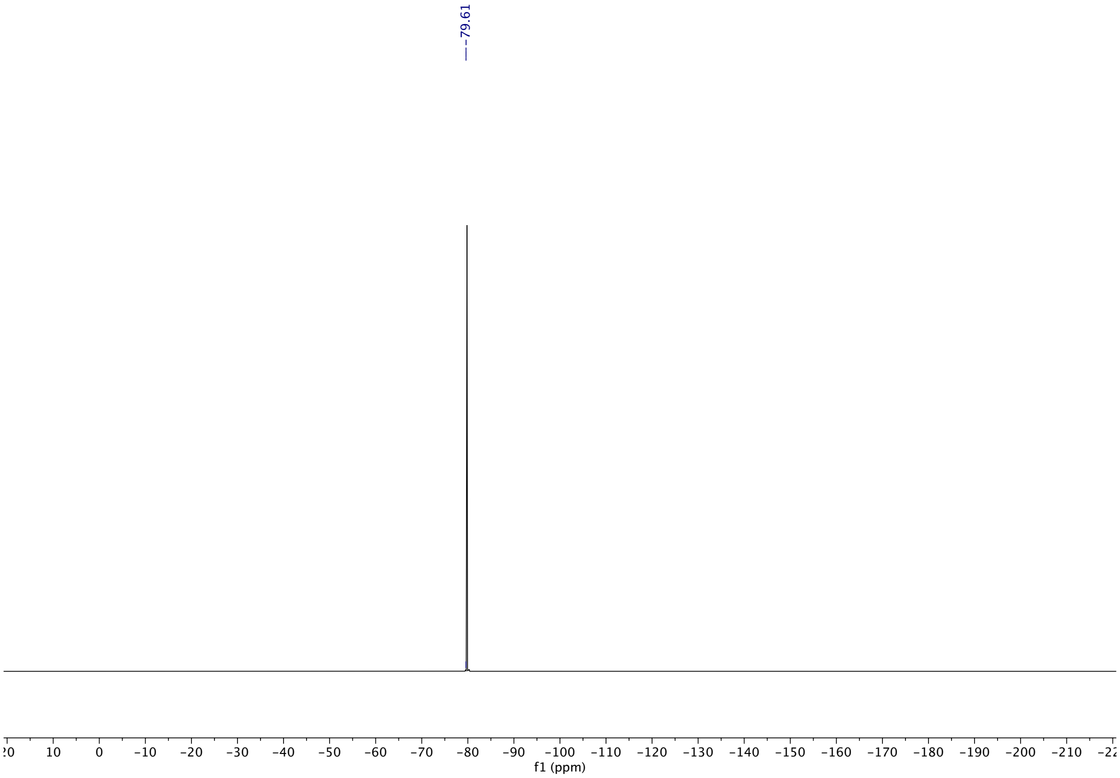

Figure S19. $^{19}F\{^{1}H\}$ NMR spectrum (THF-d_8, 470 MHz) of Complex **4**.

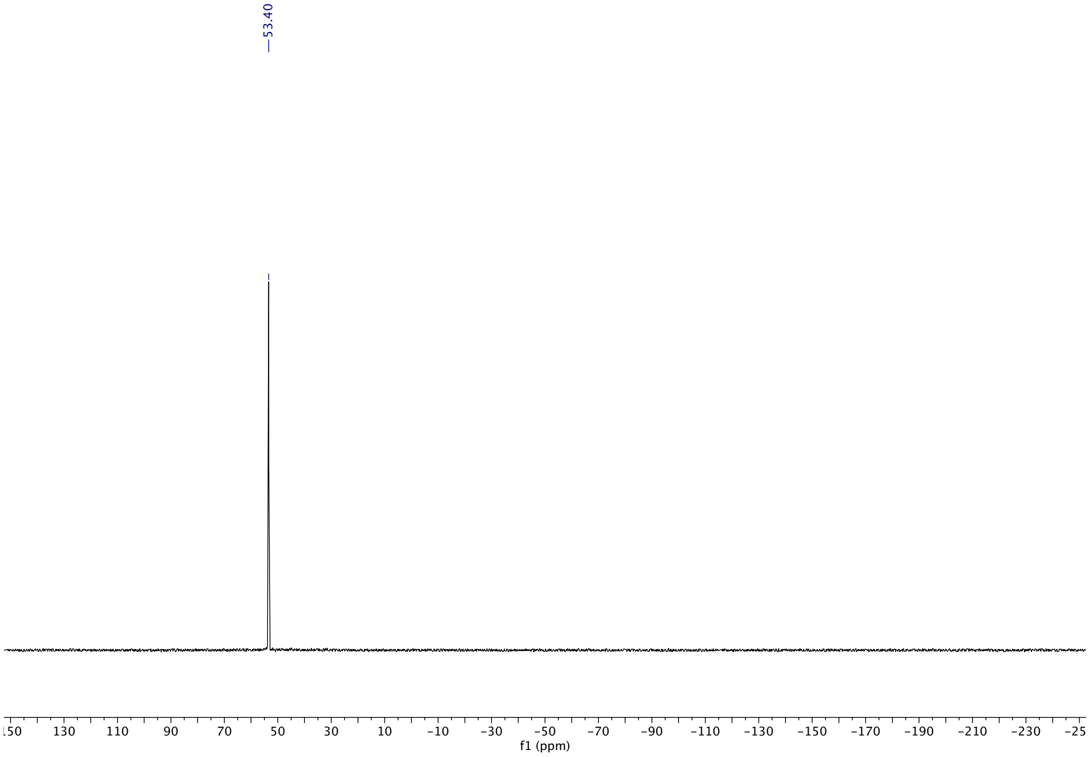

Figure S20. $^{31}P\{^{1}H\}$ NMR spectrum (THF-d_8, 202 MHz) of Complex **4**.

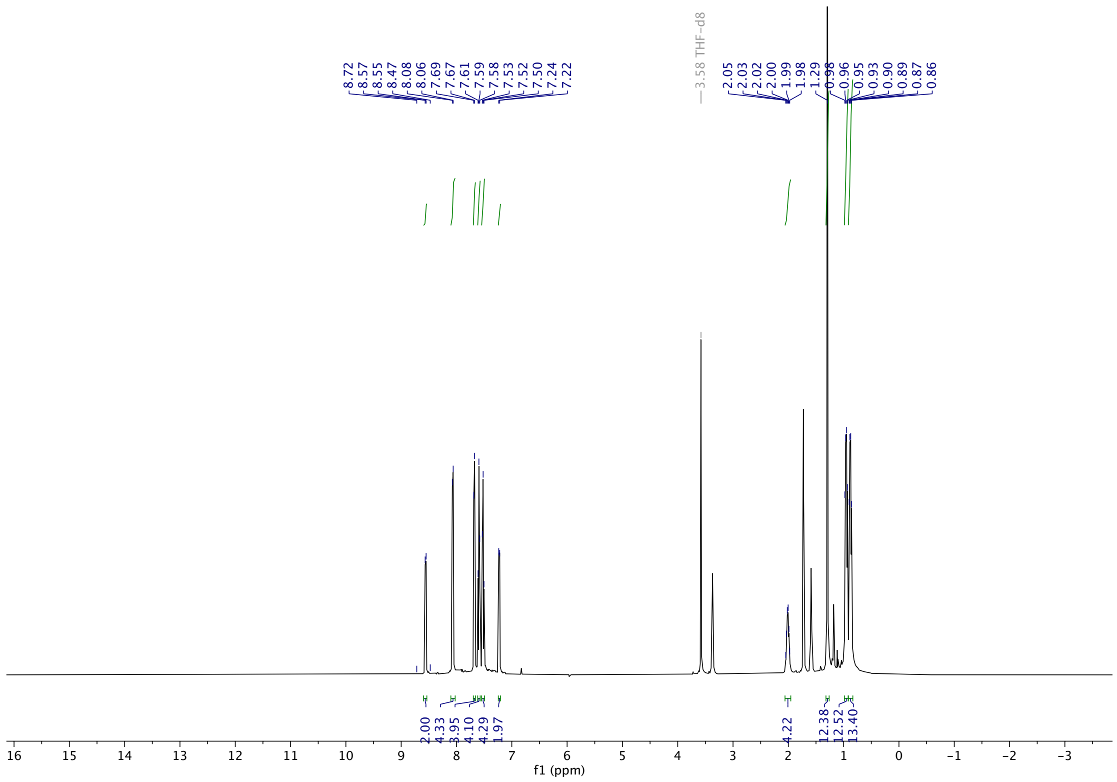

Figure S21. ^{1}H NMR spectrum (THF-d_8, 500 MHz) of Complex **5**.

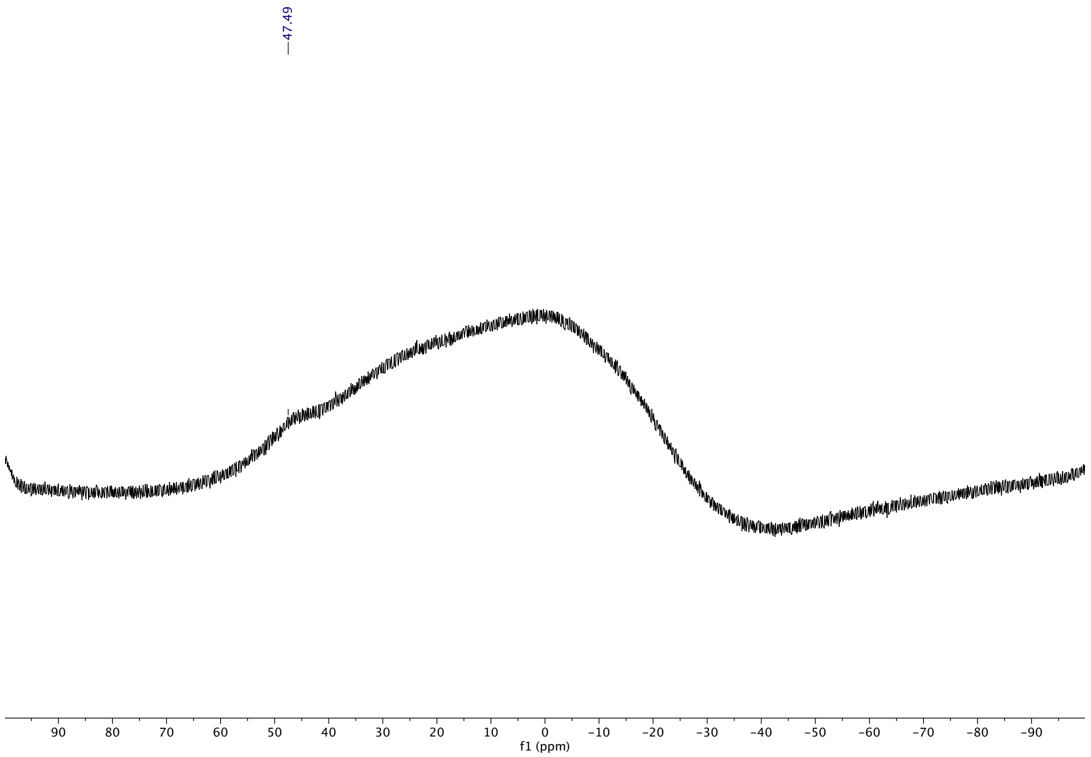

Figure S22. $^{11}B\{^{1}H\}$ NMR spectrum (THF-d_8, 160 MHz) of Complex **5**.

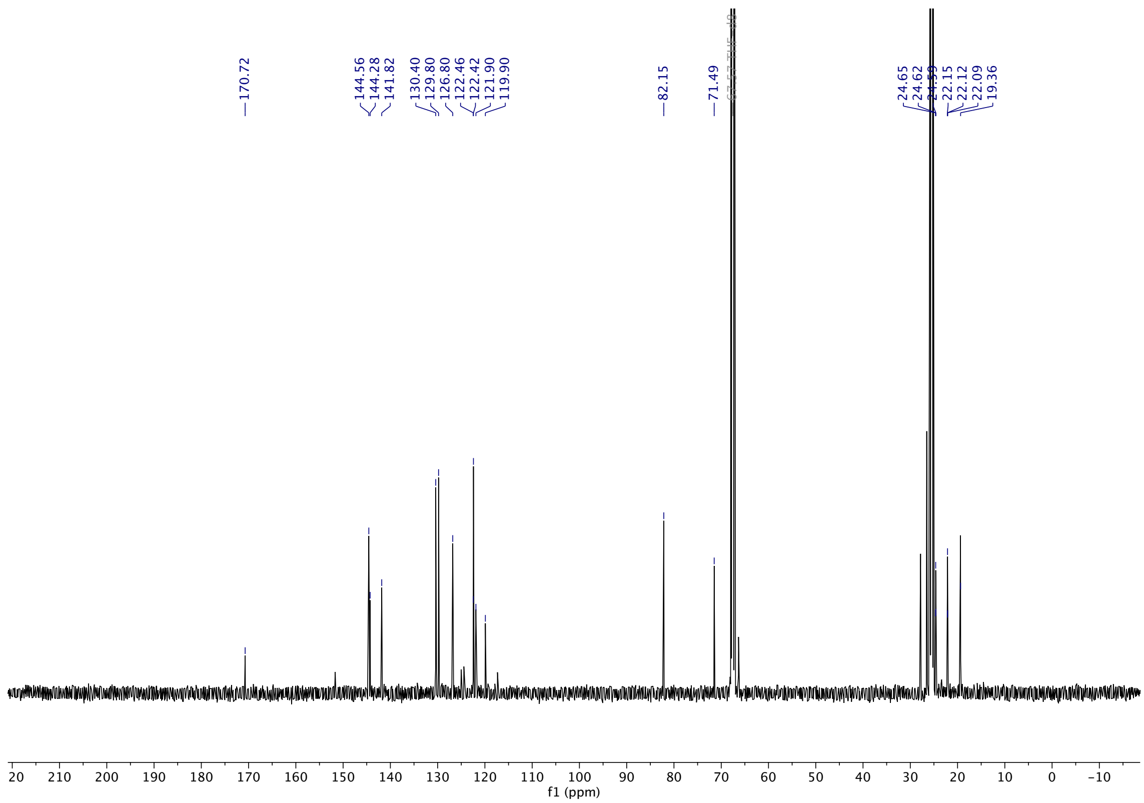

Figure S23. $^{13}C\{^1H\}$ NMR spectrum (THF-d_8, 125 MHz) of Complex **5**.

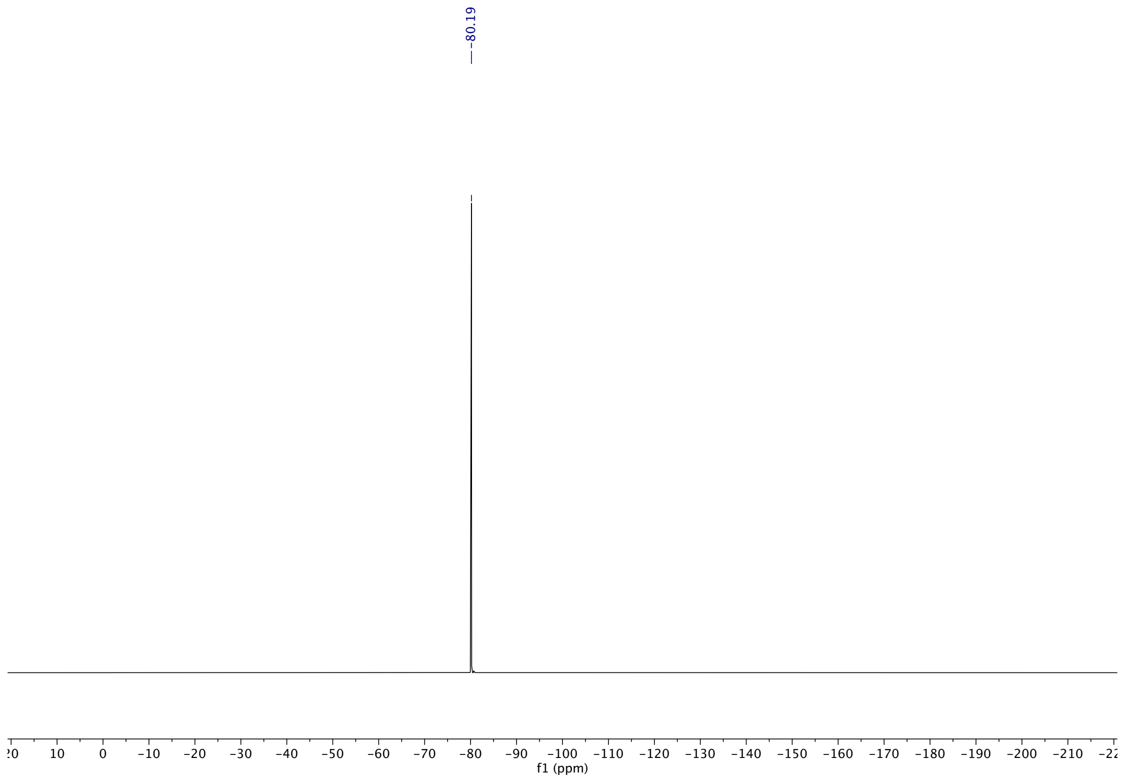

Figure S24. $^{19}F\{^1H\}$ NMR spectrum (THF-d_8, 470 MHz) of Complex **5**.

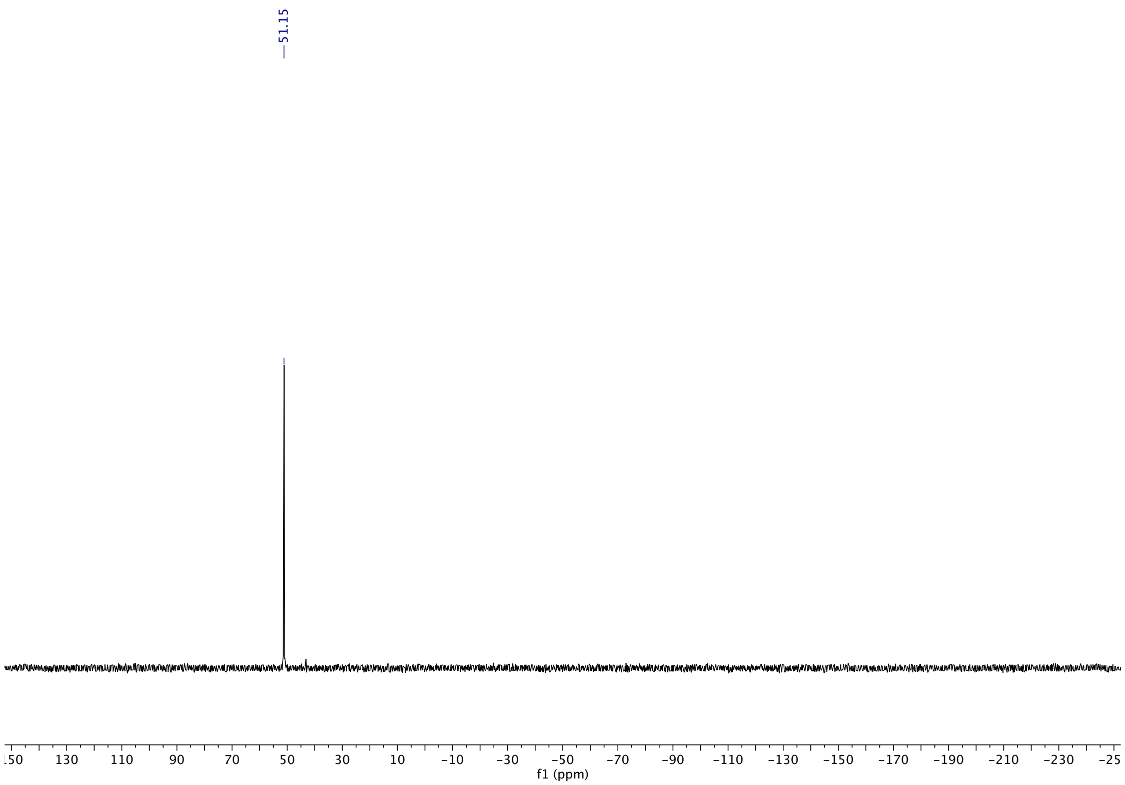

Figure S25. $^{31}P\{^{1}H\}$ NMR spectrum (THF-d_8, 202 MHz) of Complex **5**.

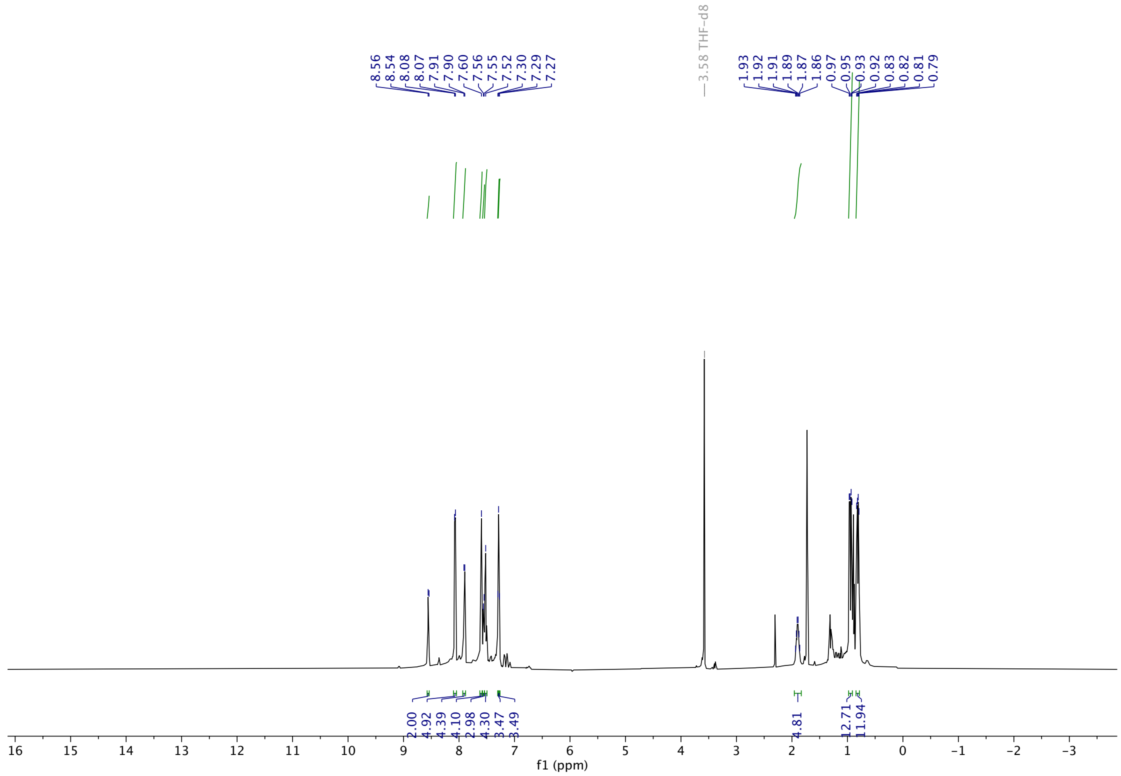

Figure S26. ^{1}H NMR spectrum (THF-d_8, 500 MHz) of Complex **6**.

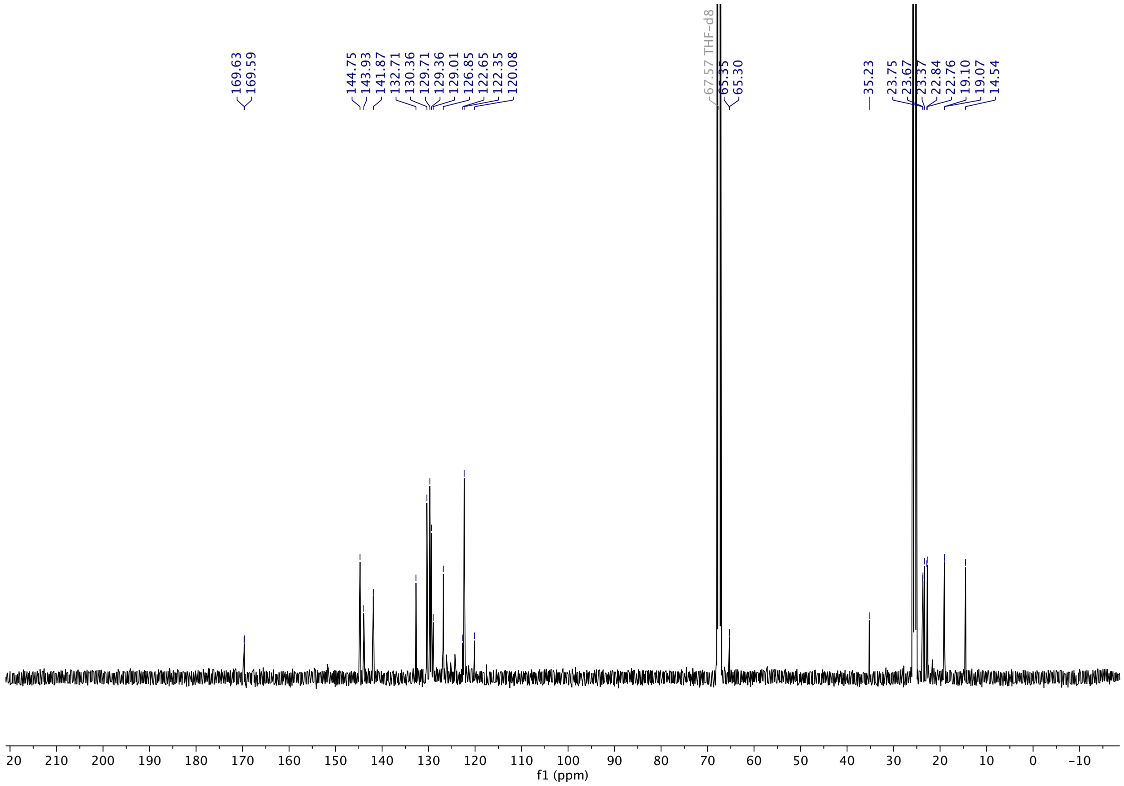

Figure S27. $^{13}C\{^{1}H\}$ NMR spectrum (THF-d_8, 125 MHz) of Complex **6**.

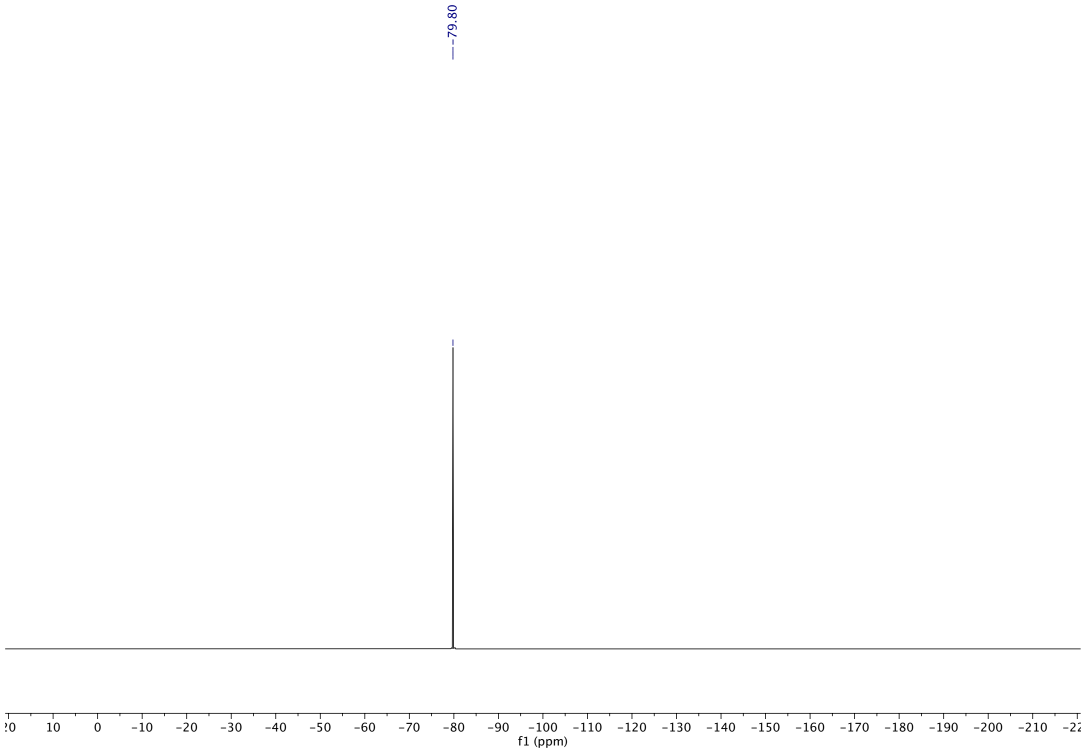

Figure S28. $^{19}F\{^{1}H\}$ NMR spectrum (THF-d_8, 470 MHz) of Complex **6**.

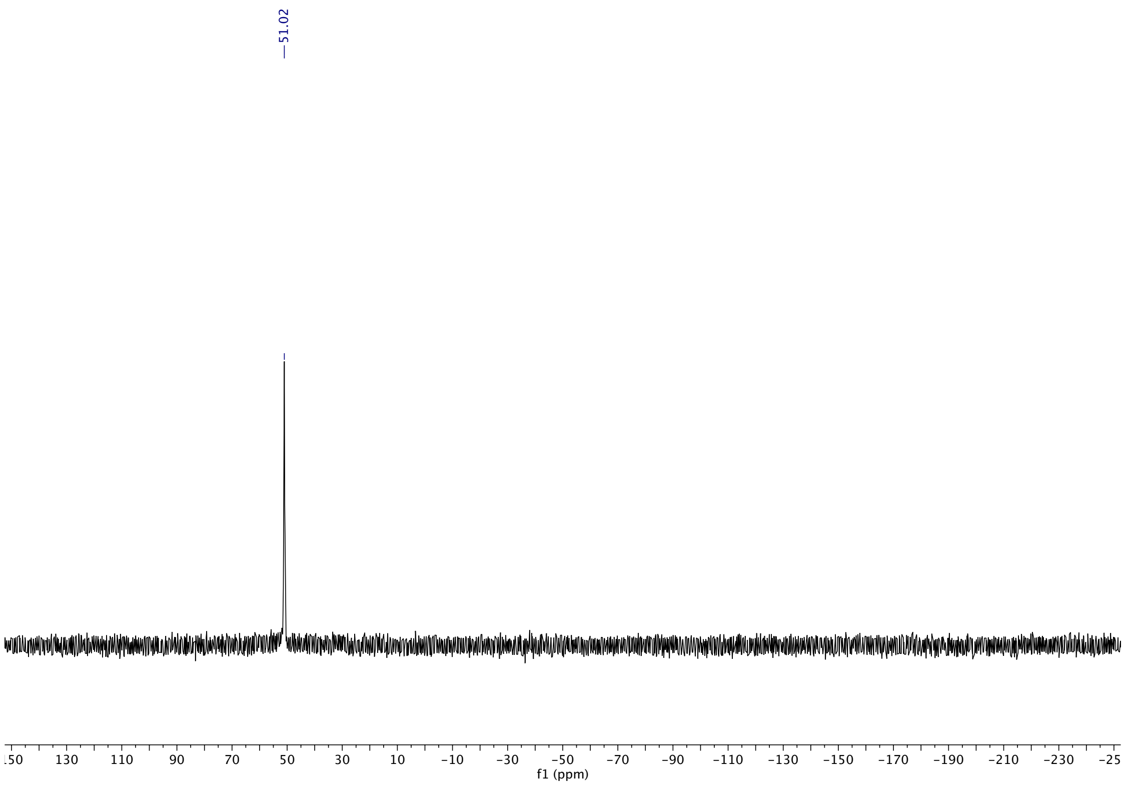

Figure S29. $^{31}P\{^{1}H\}$ NMR spectrum (THF-d_8, 202 MHz) of Complex **6**.

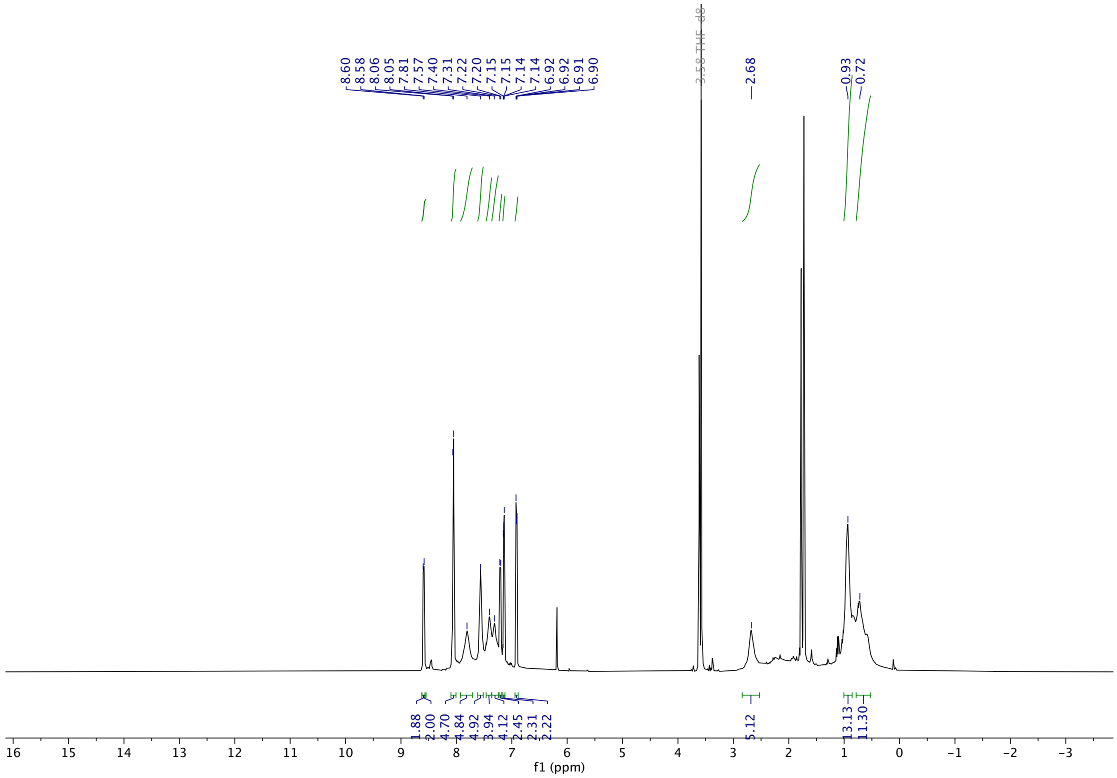

Figure S30. ^{1}H NMR spectrum (THF-d_8, 500 MHz) of Complex **7**.

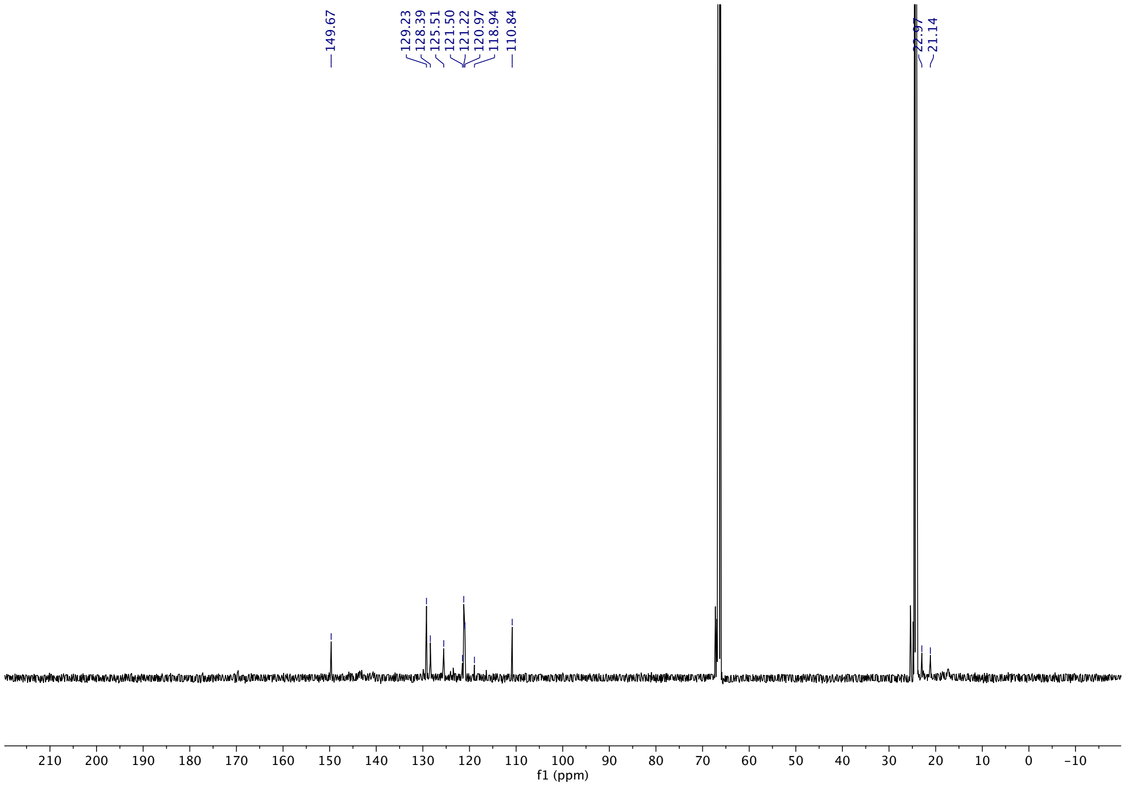

Figure S31. $^{13}C\{^{1}H\}$ NMR spectrum (THF-d_8, 125 MHz) of Complex **7**.

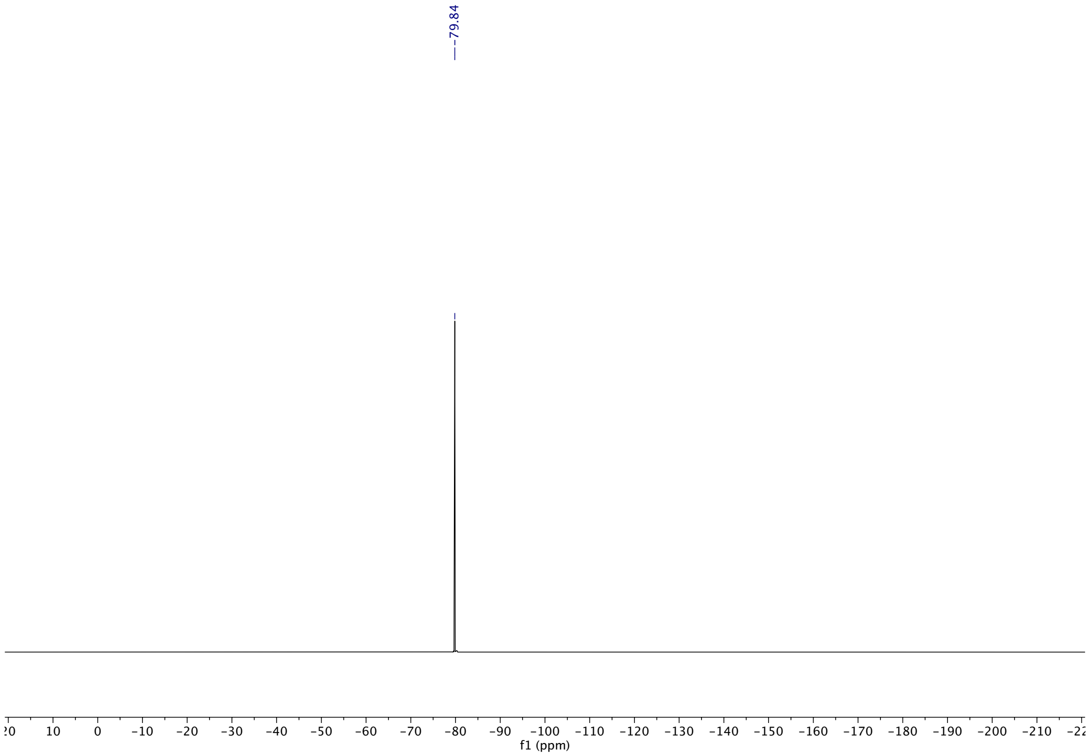

Figure S32. $^{19}F\{^{1}H\}$ NMR spectrum (THF-d_8, 470 MHz) of Complex **7**.

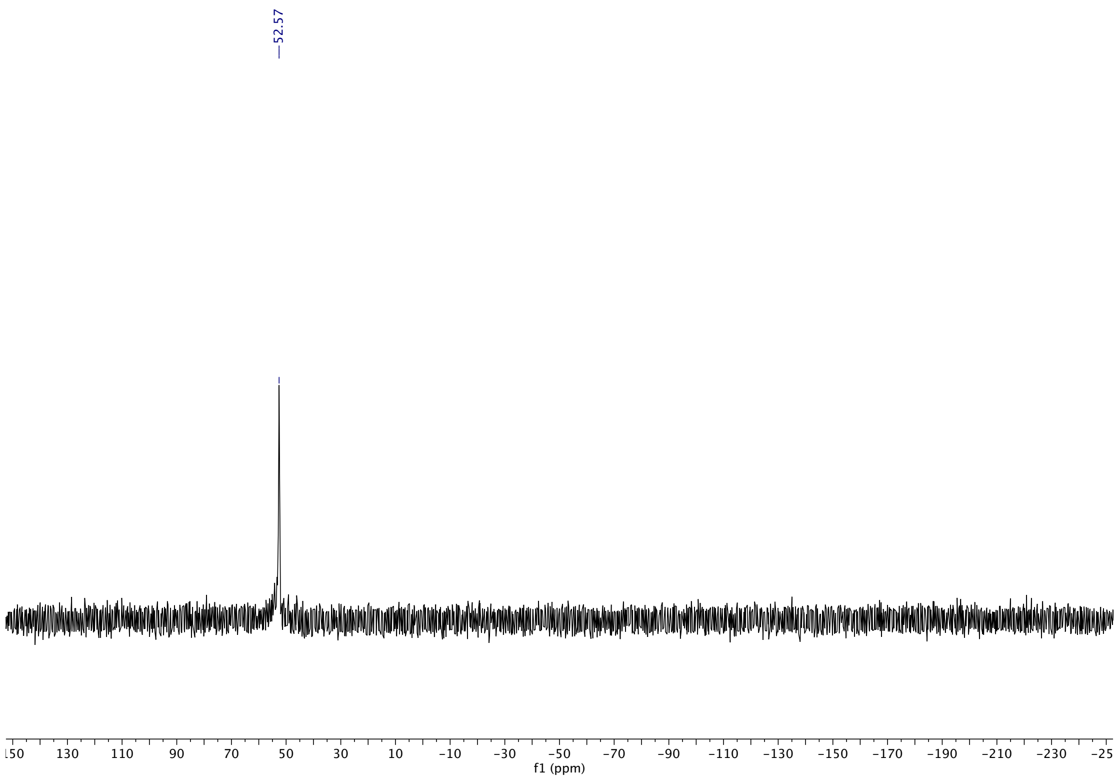

Figure S33. $^{31}P\{^{1}H\}$ NMR spectrum (THF-d_8, 202 MHz) of Complex **7**.

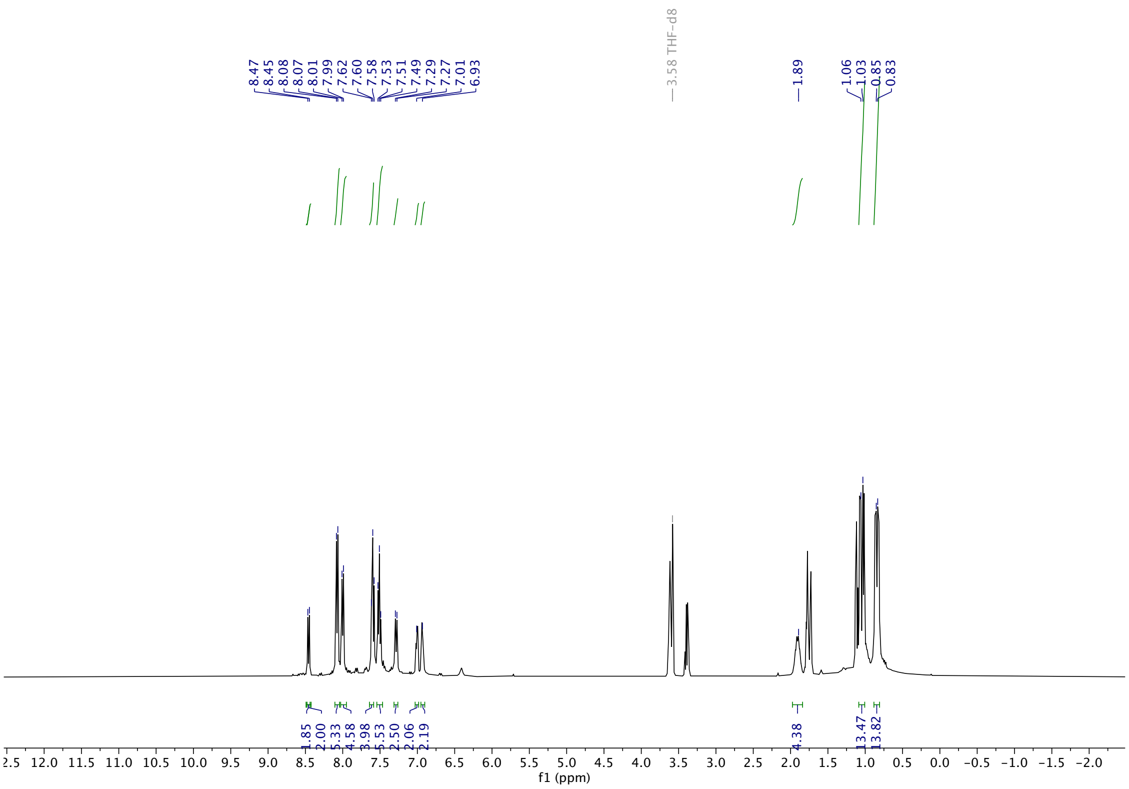

Figure S34. ^{1}H NMR spectrum (THF-d_8, 500 MHz) of Complex **8**.

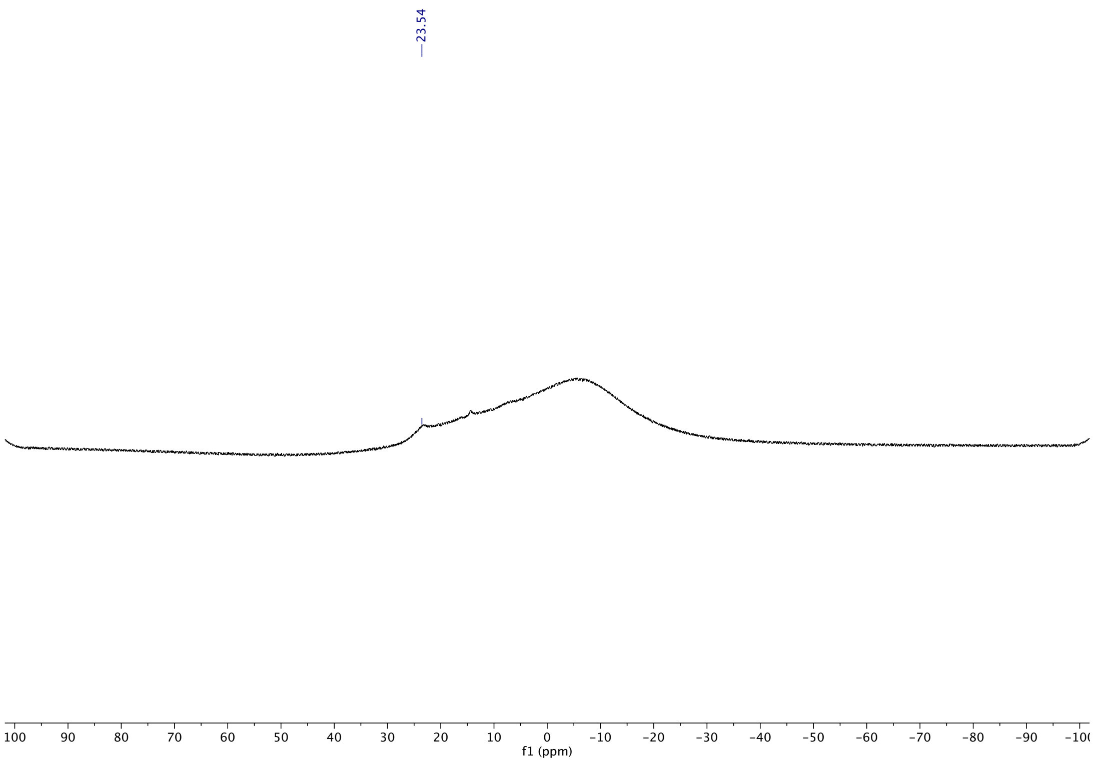

Figure S35. $^{11}B\{^{1}H\}$ NMR spectrum (THF-d_8, 160 MHz) of Complex **8**.

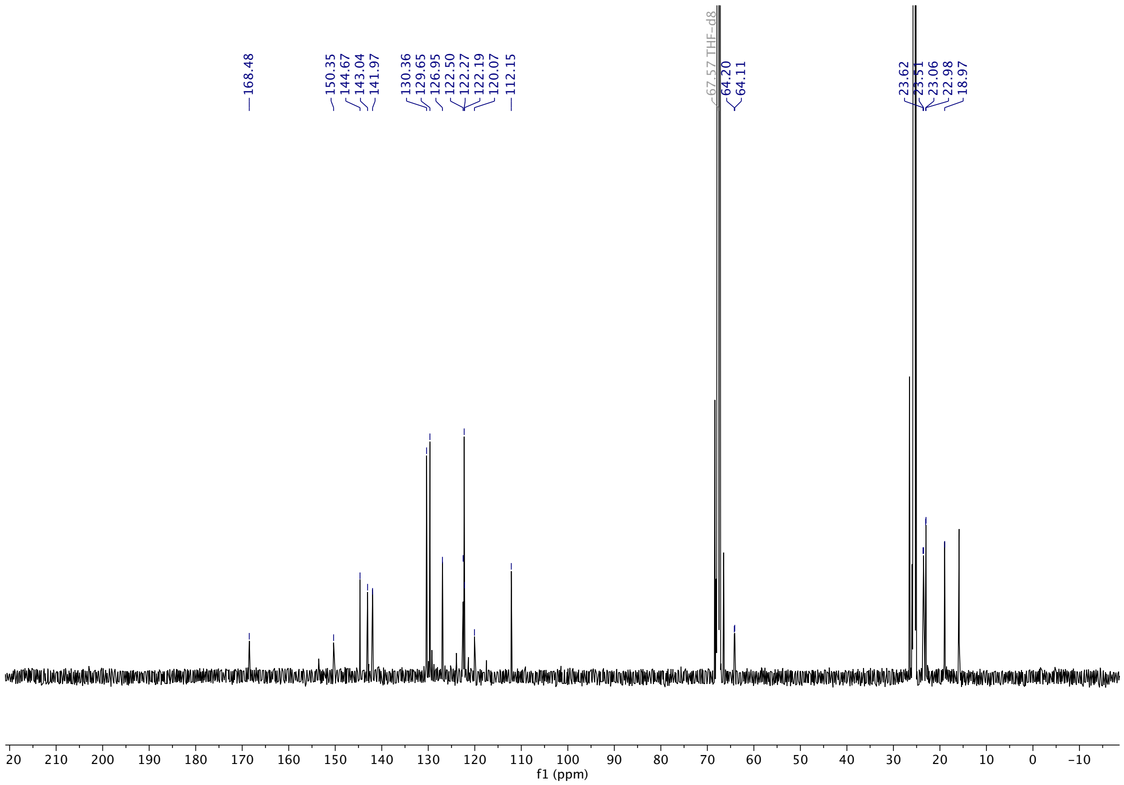

Figure S36. $^{13}C\{^{1}H\}$ NMR spectrum (THF-d_8, 125 MHz) of Complex **8**.

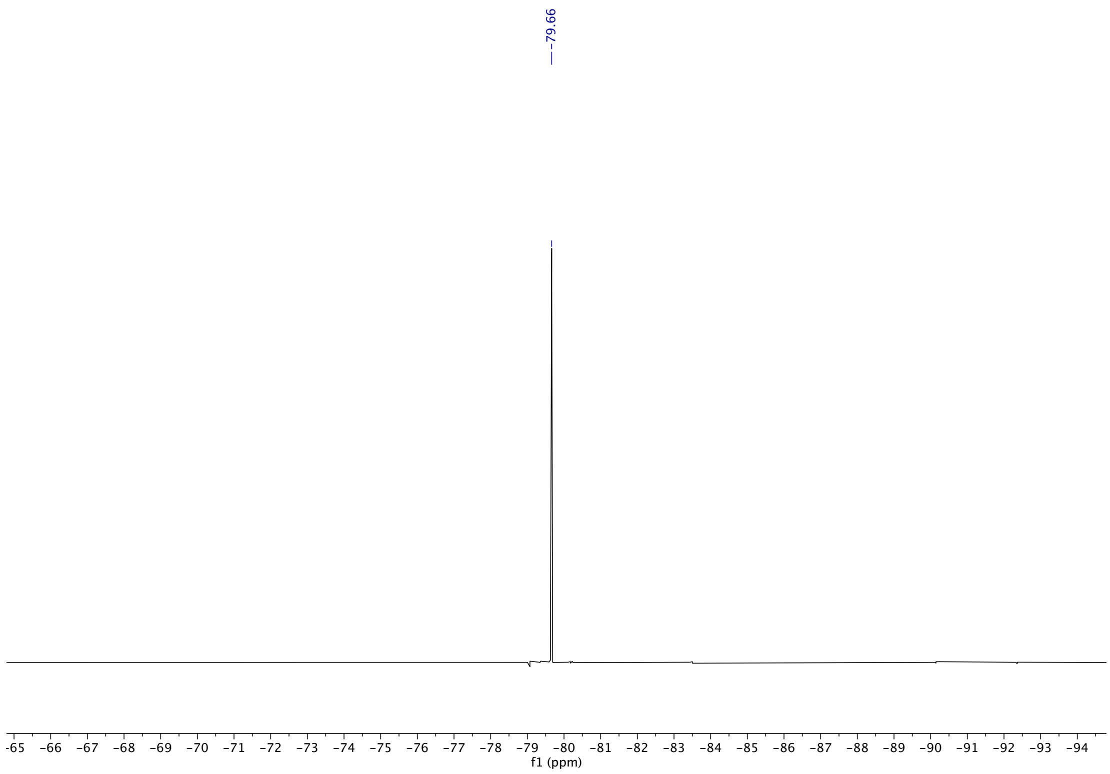

Figure S37. $^{19}F\{^1H\}$ NMR spectrum (THF-d_8, 470 MHz) of Complex **8**.

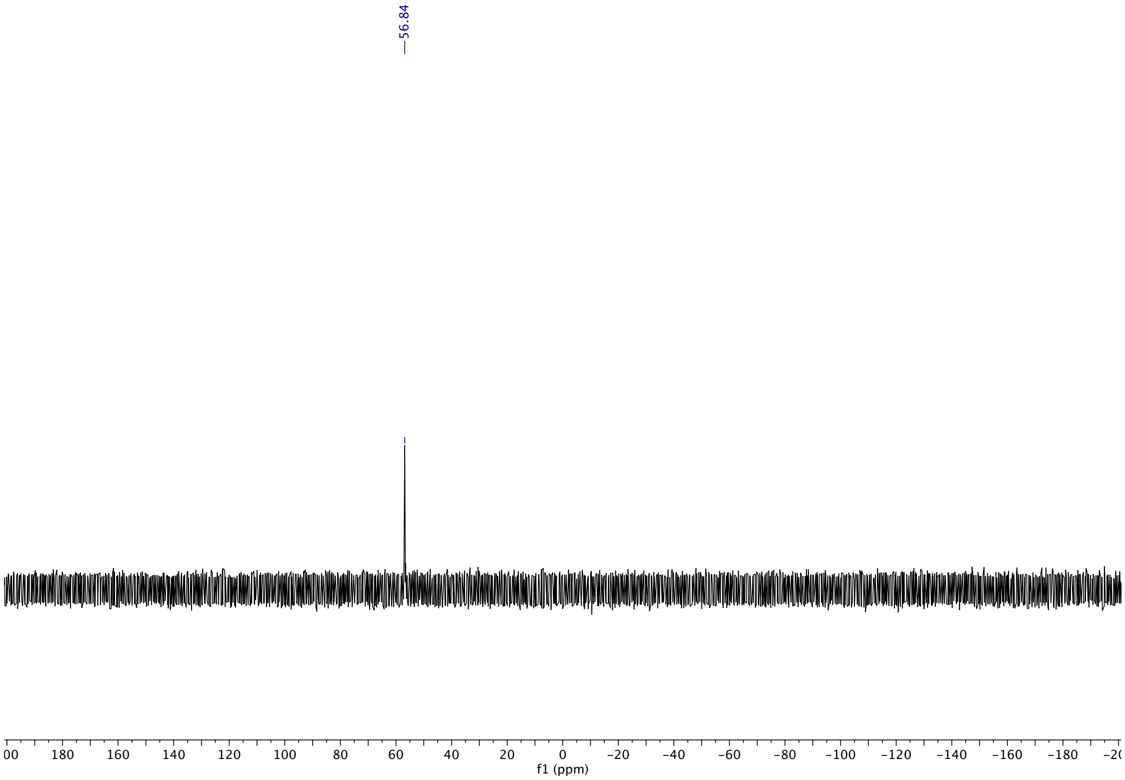

Figure S38. $^{31}P\{^1H\}$ NMR spectrum (THF-d_8, 202 MHz) of Complex **8**.

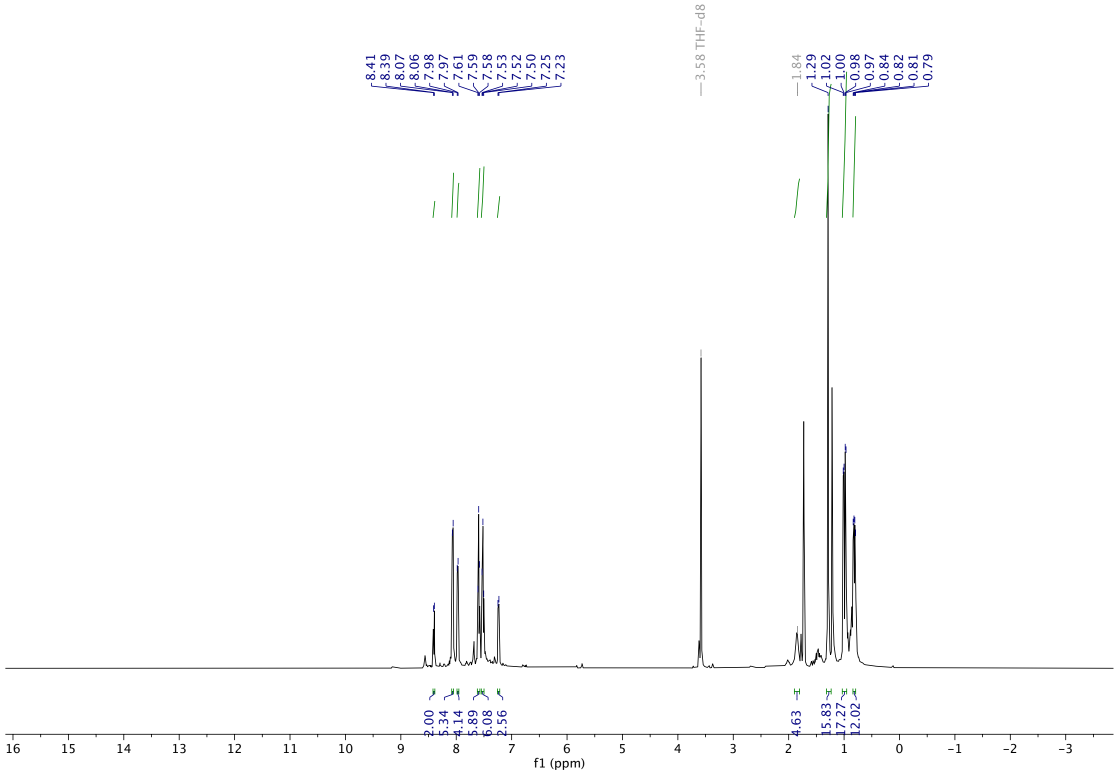

Figure S39. ^{1}H NMR spectrum (THF-d_8, 500 MHz) of Complex **9**.

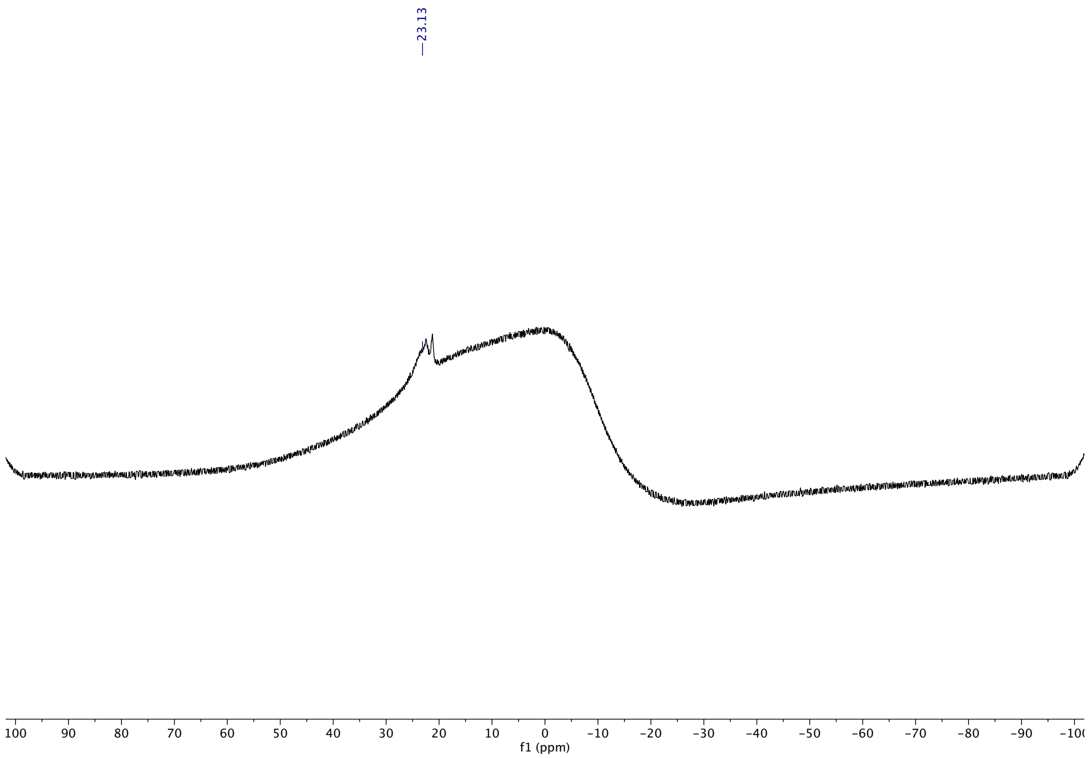

Figure S40. ^{11}B{^{1}H} NMR spectrum (THF-d_8, 160 MHz) of Complex **9**.

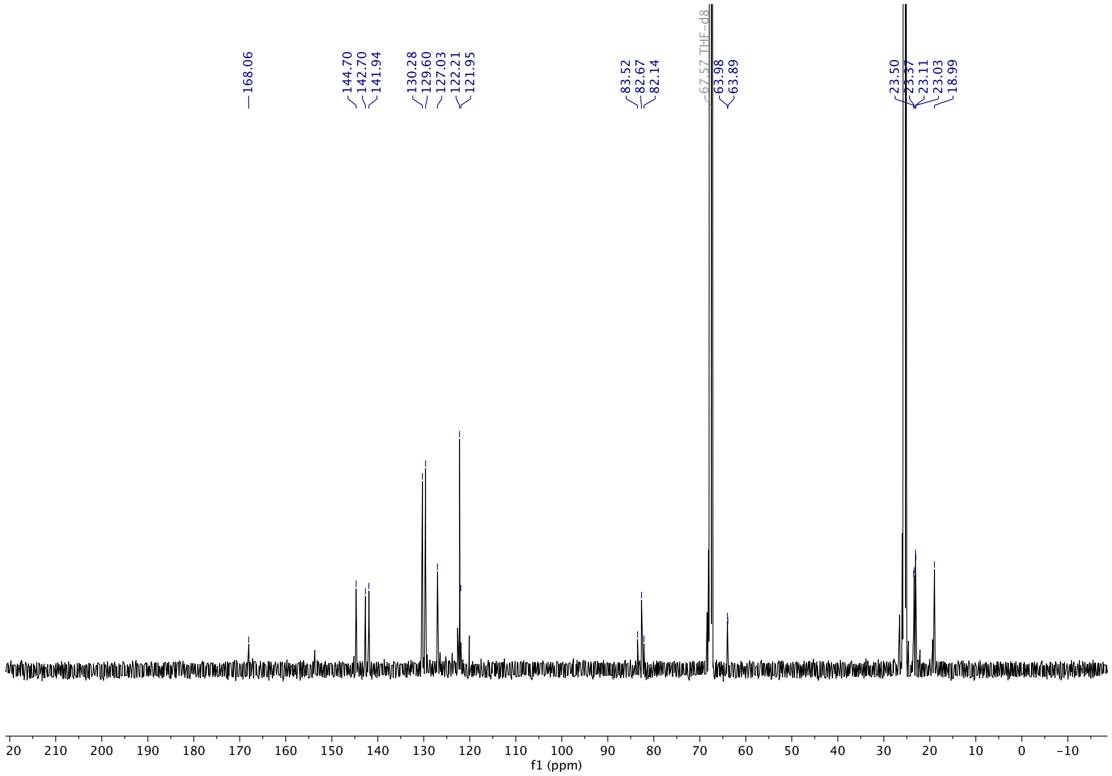

Figure S41. $^{13}C\{^{1}H\}$ NMR spectrum (THF-d_8, 125 MHz) of Complex **9**.

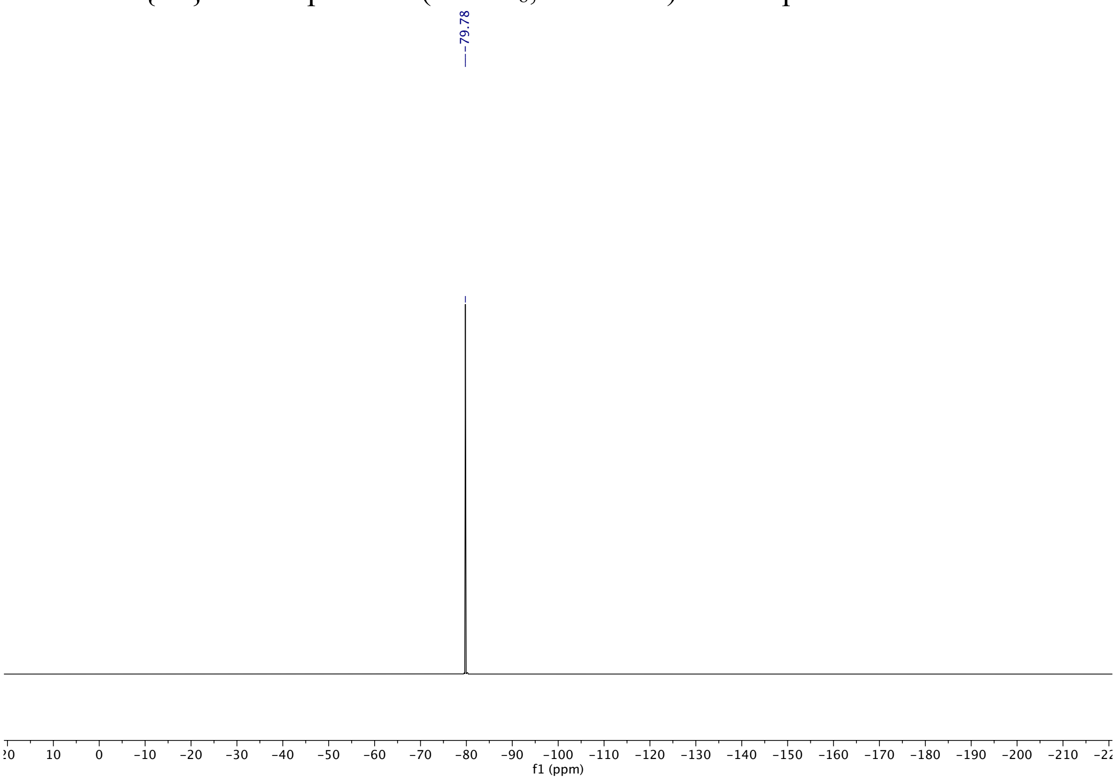

Figure S42. $^{19}F\{^{1}H\}$ NMR spectrum (THF-d_8, 470 MHz) of Complex **9**.

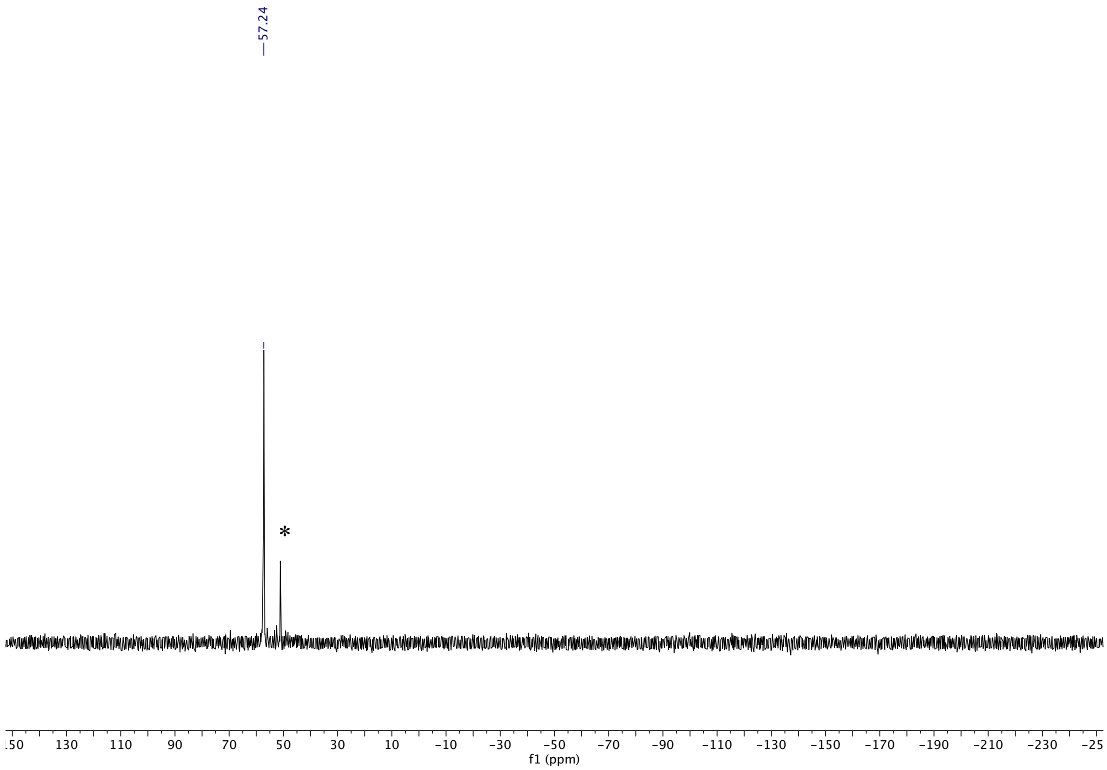

Figure S43. $^{31}P\{^{1}H\}$ NMR spectrum (THF-d_8, 202 MHz) of Complex **9**. *indicates residual Complex **5**.

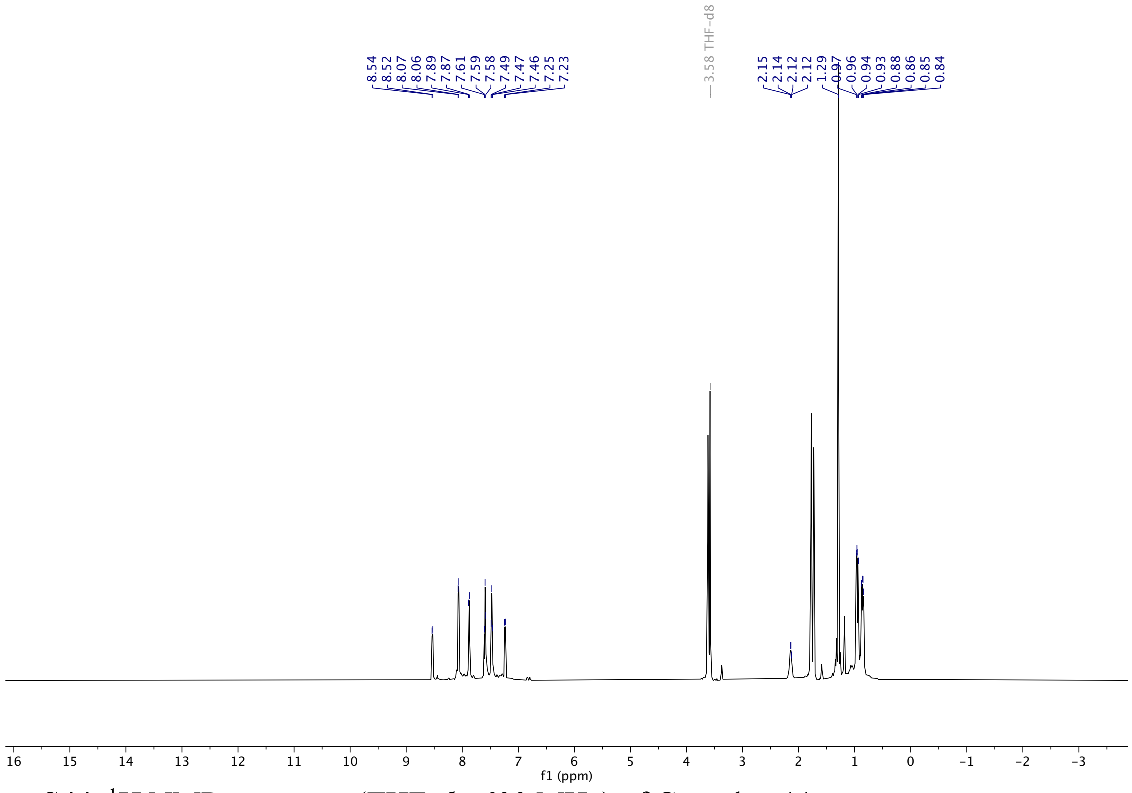

Figure S44. ^{1}H NMR spectrum (THF-d_8, 600 MHz) of Complex **11**.

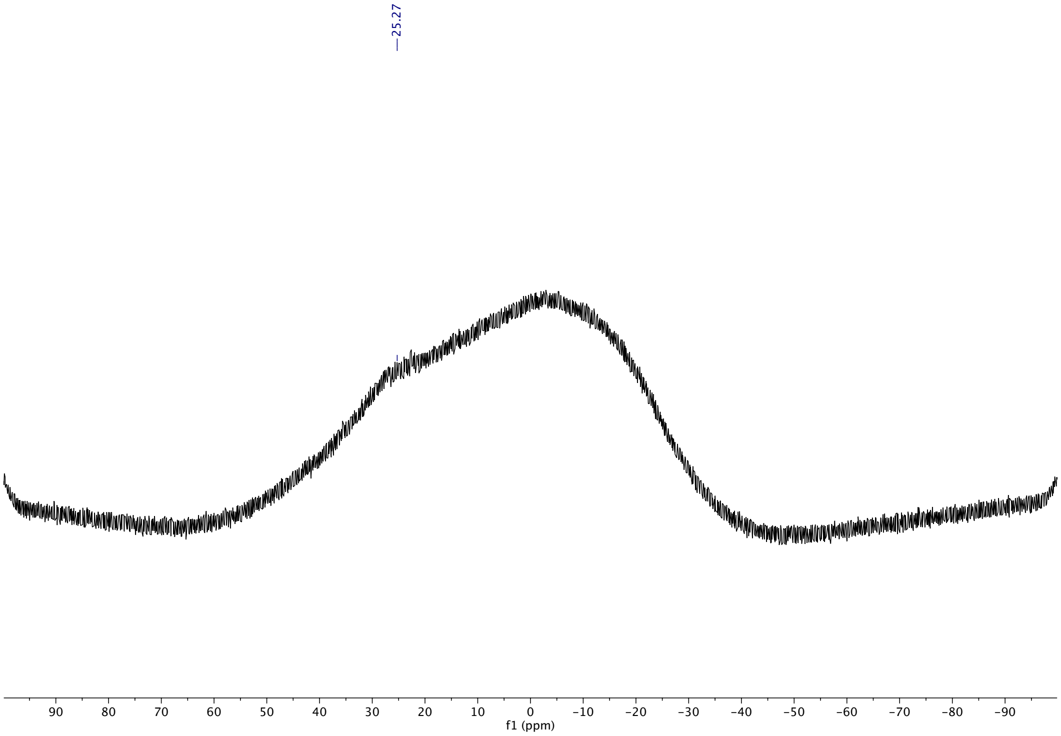

Figure S45. $^{11}B\{^{1}H\}$ NMR spectrum (THF-d_8, 160 MHz) of Complex **11**.

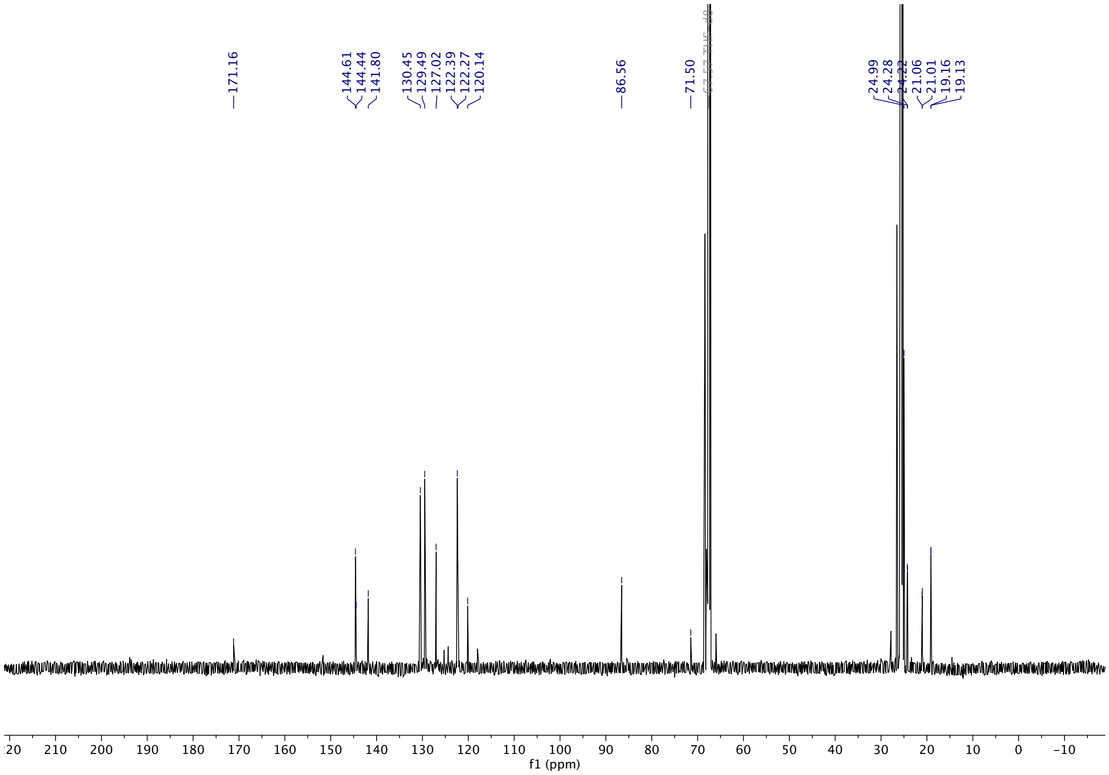

Figure S46. $^{13}C\{^{1}H\}$ NMR spectrum (THF-d_8, 151 MHz) of Complex **11**.

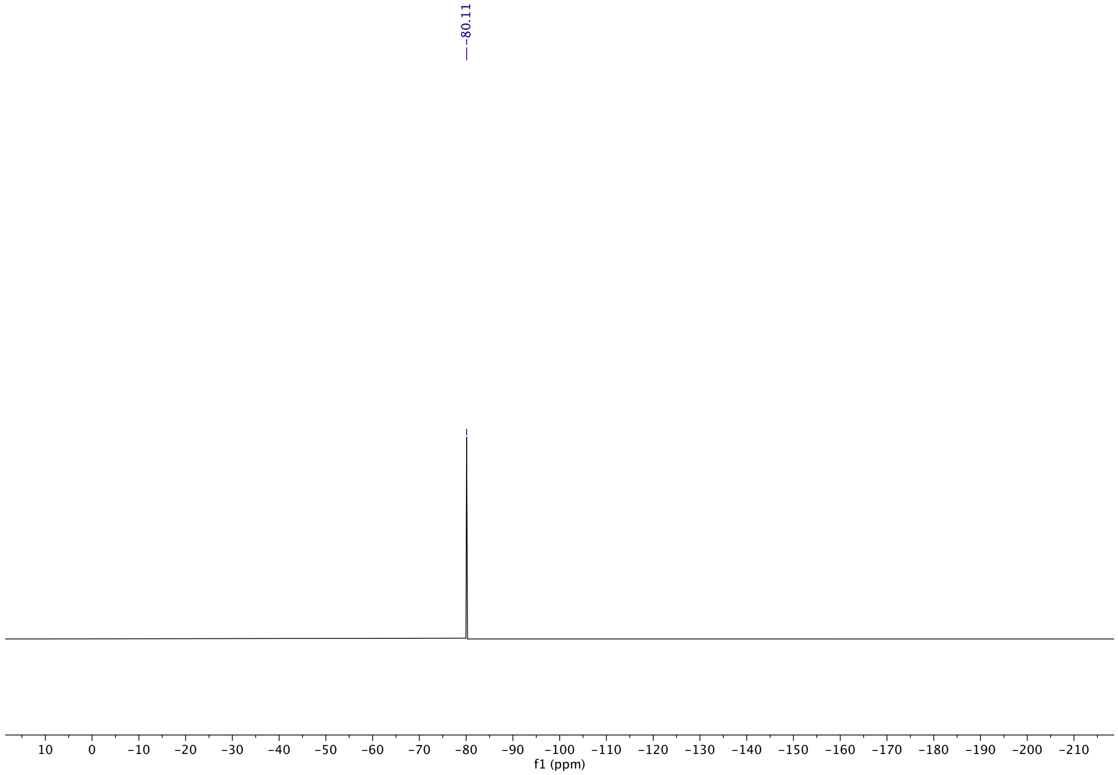

Figure S47. $^{19}F\{^1H\}$ NMR spectrum (THF-d_8, 565 MHz) of Complex **11**.

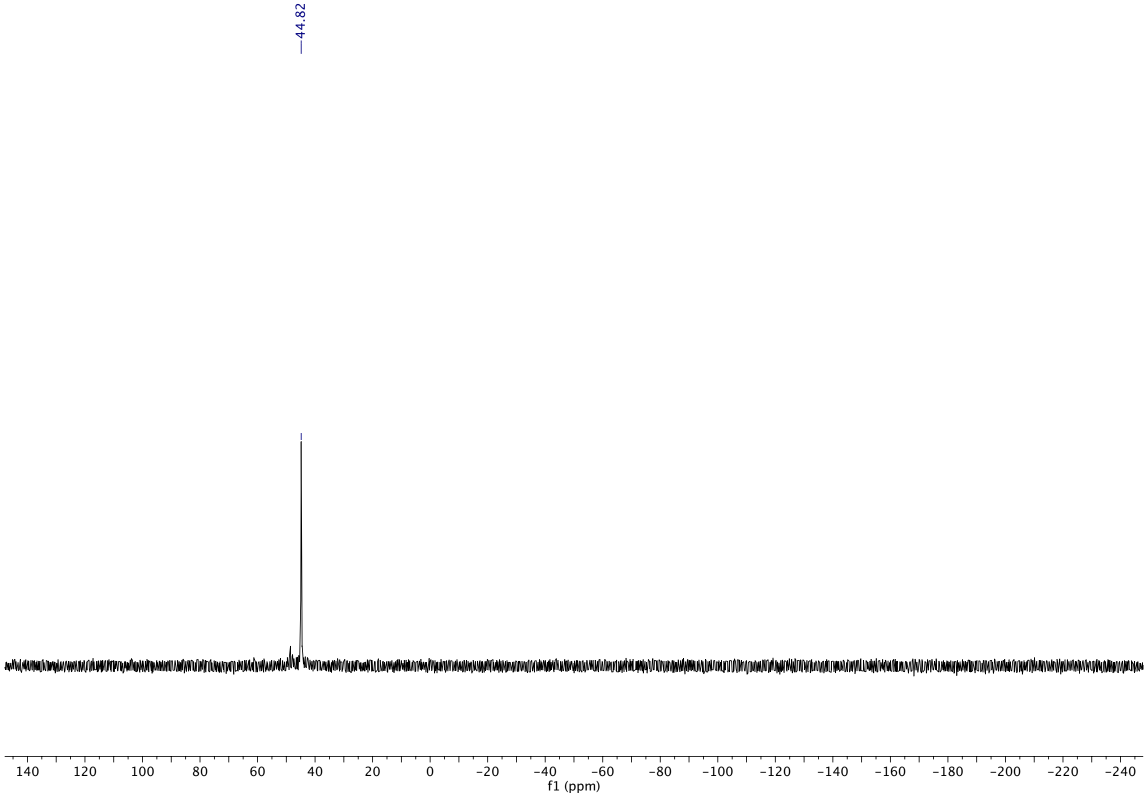

Figure S48. $^{31}P\{^1H\}$ NMR spectrum (THF-d_8, 162 MHz) of Complex **11**.

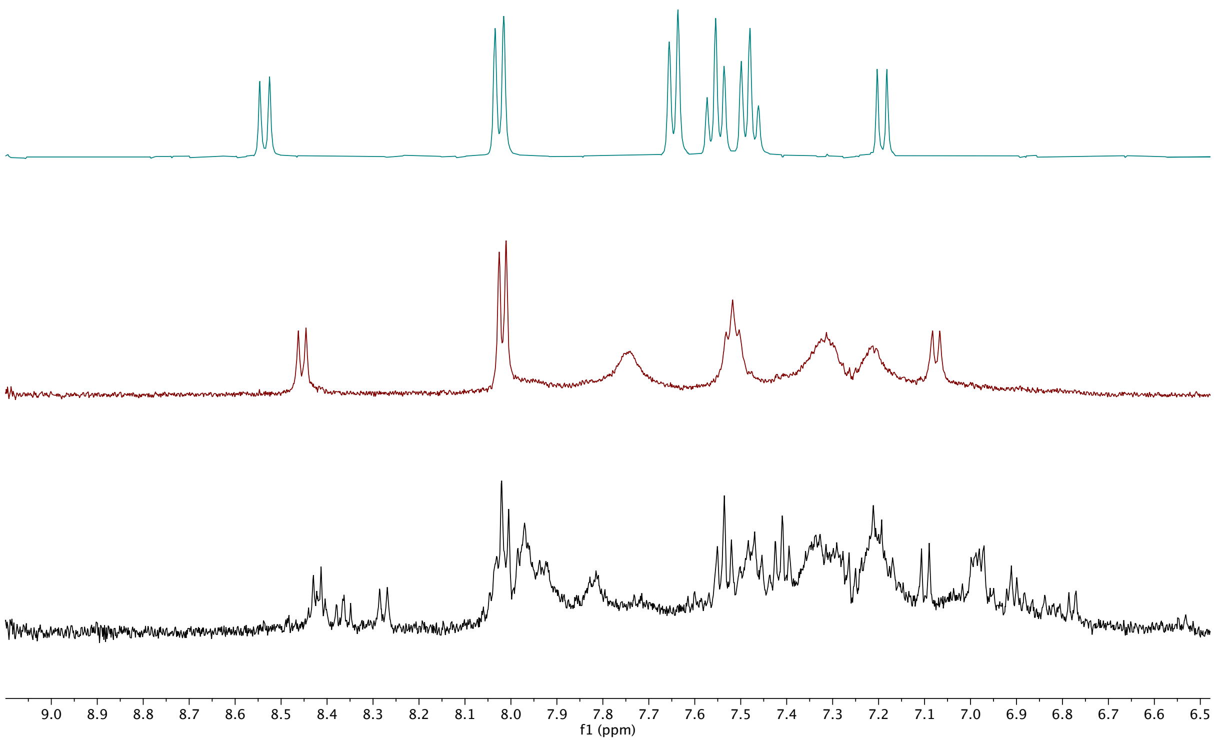

Figure S49. ^{1}H NMR spectra (THF-h_8, 400 MHz) of Complex **5** (top, blue), 30 minutes after the addition of 1.0 equivalent of xylyl isocyanide to **5** (middle, red), and 3 d stirring in solution after xylyl isocyanide addition (bottom, black).

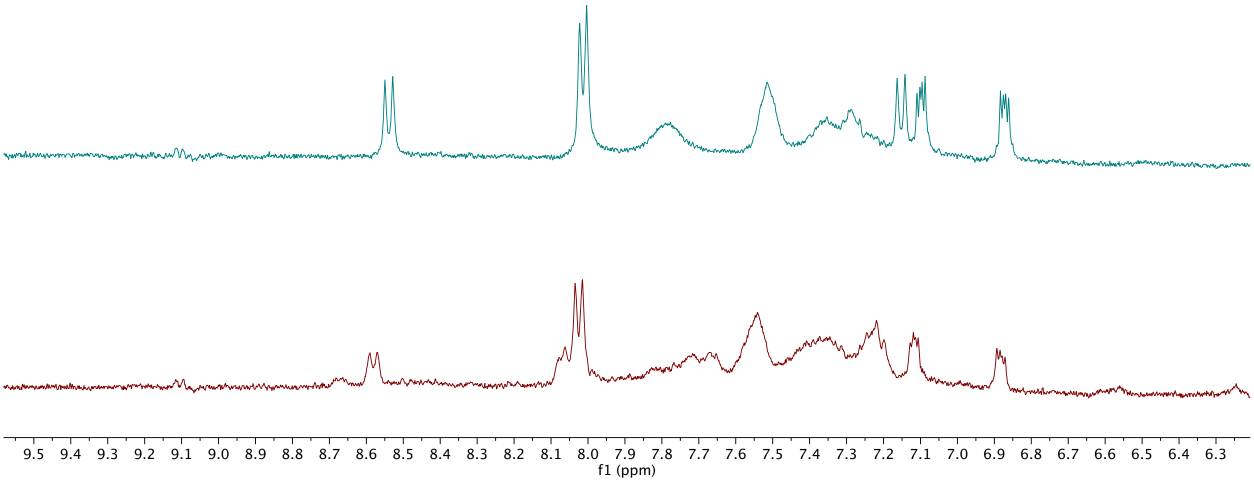

Figure S50. ^{1}H NMR spectra (THF-h_8, 400 MHz) of Complex **7** (top, blue) and after storing in the solid state for 71 d (bottom, red).

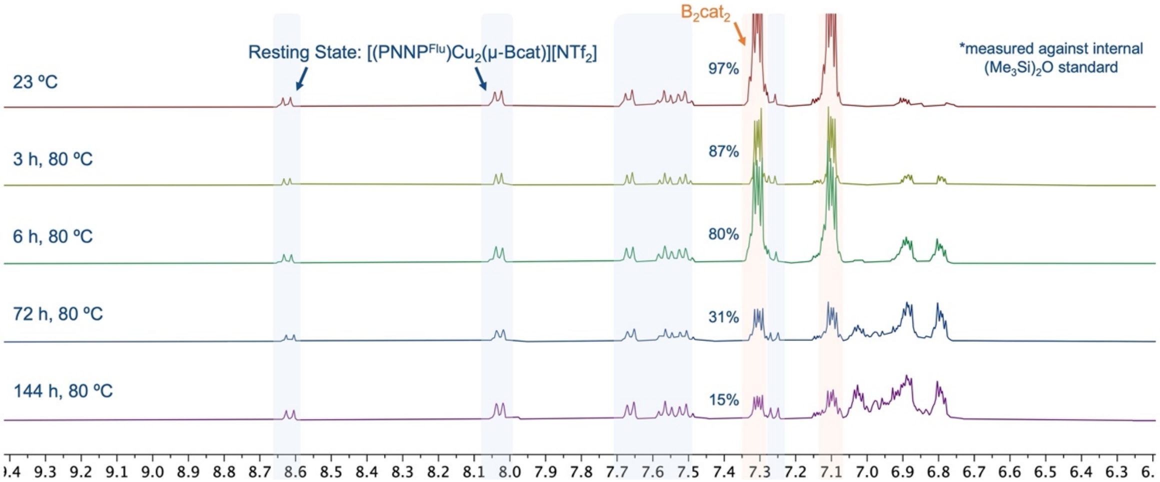

Figure S51. ^{1}H NMR spectra (THF-h_8, 400 MHz) of Complex **4** with 5.0 equivalents B$_2$cat$_2$ with 1 atm CO$_2$.

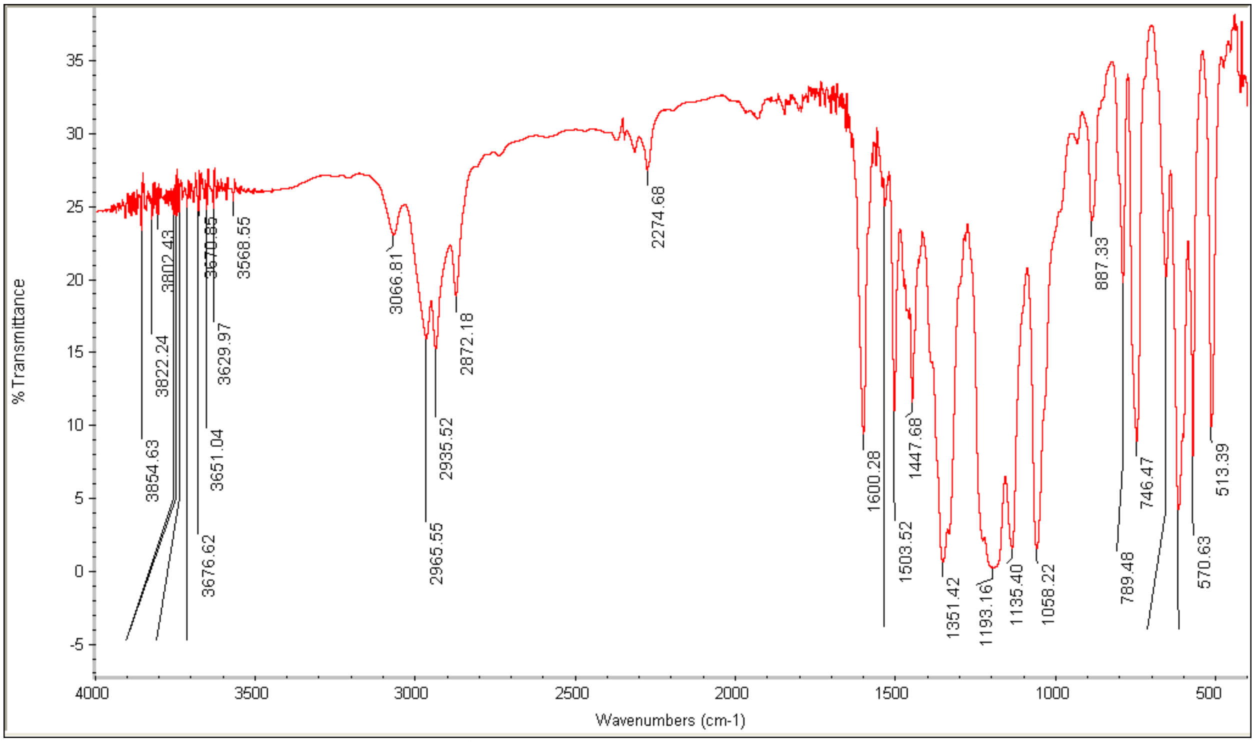

Figure S52. IR spectrum (KBr, cm^{-1}) of Complex **1**.

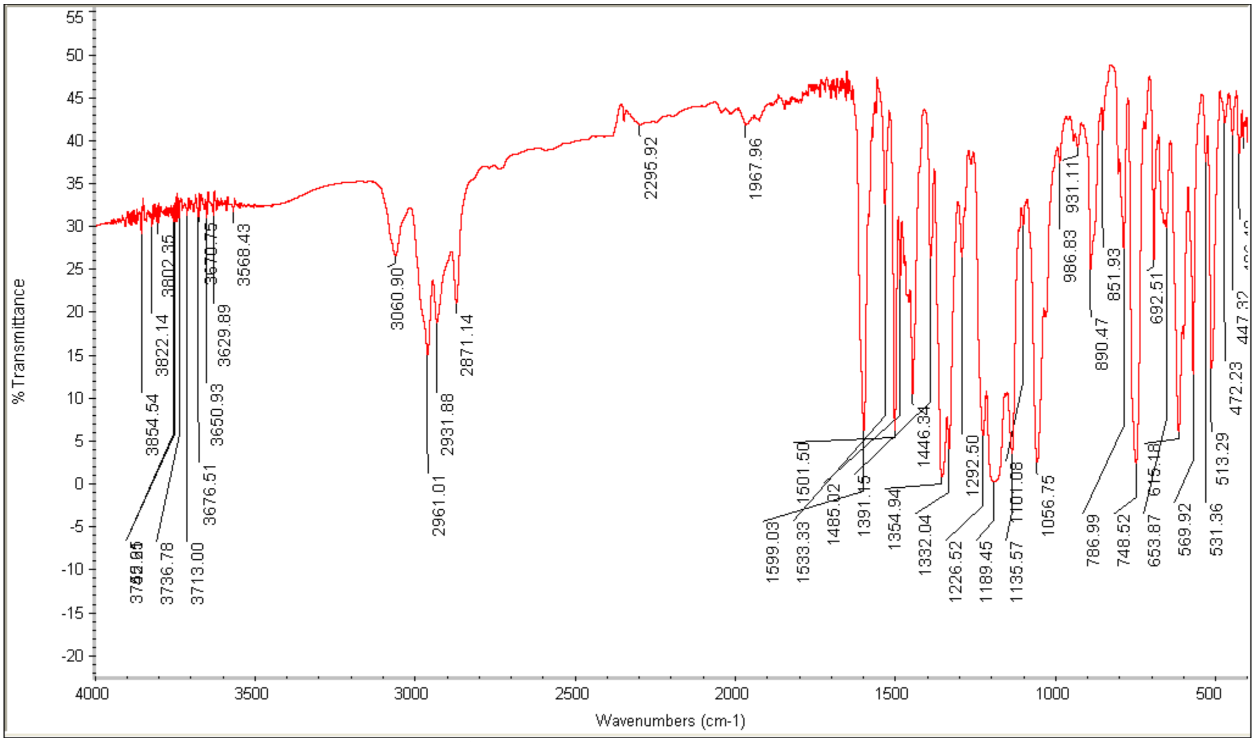

Figure S53. IR spectrum (KBr, cm^{-1}) of Complex **6**.

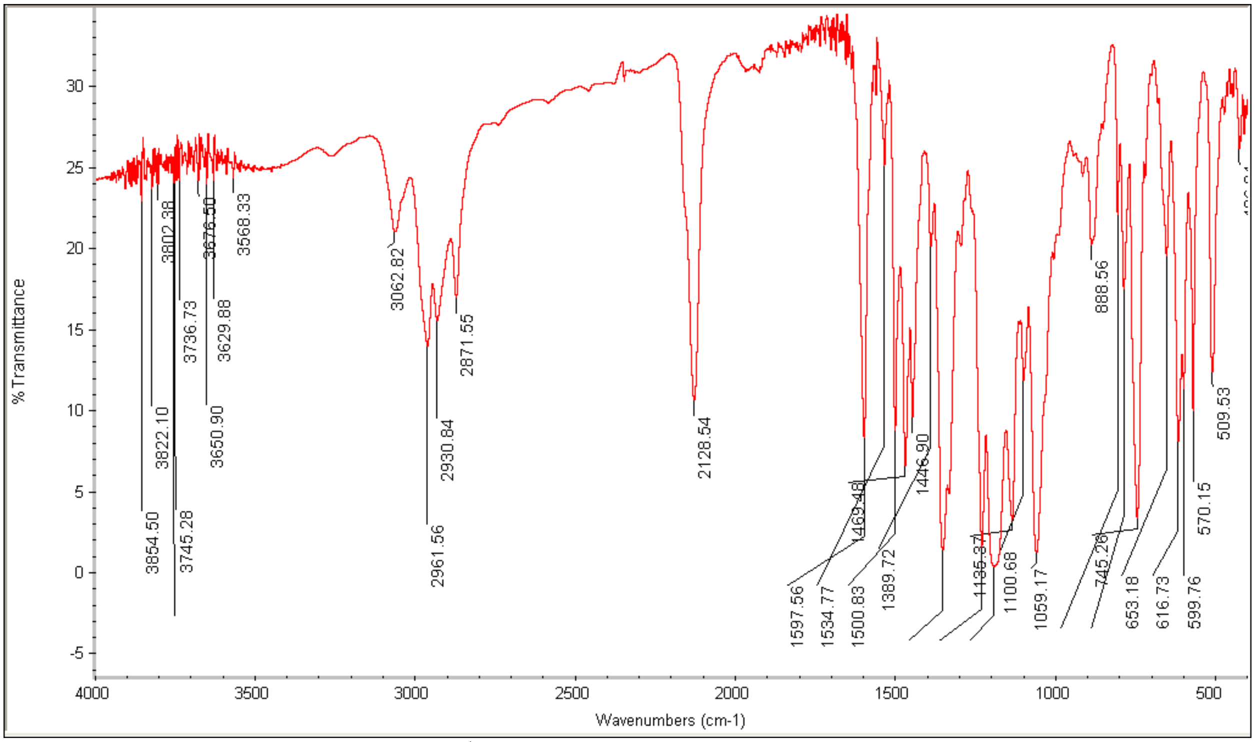

Figure S54. IR spectrum (KBr, cm^{-1}) of Complex **7**.

X-ray Crystallography:

General Considerations: Crystalline samples were prepared in a glovebox by decanting residual supernatant and immersing the crystals under a protective layer of Paratone N oil. All samples were frozen in a container of dry ice prior to data collection. Data for **2**, **3**, **4**, **5**, **6**, **7**, **9**, **10**, and **11** were collected at the Advanced Light Source beamline 12.2.1 using a Bruker D85 three-circle diffractometer equipped with a PHOTON II CCD area detector using synchrotron radiation (λ = 0.7288 Å) from a Si(111) double crystal Si(111) monochromator. Data for **8** was collected at the Molecular Foundry at Lawrence Berkeley National Laboratory on a Bruker D8 Venture diffractometer using Mo Kα radiation (λ = 0.71073 Å). Structures were solved by intrinsic phasing using the SHELXT[8] software package and refined using SHELXL[9] in the OLEX2 interface.[10]

CCDC 2323738-2323747 contain the supplementary crystallographic data for this paper. These data can be obtained free of charge via www.ccdc.cam.ac.uk/dat_request/cif, or by emailing data_request@ccdc.cam.ac.uk, or by contacting the Cambridge Crystallographic Data Centre, 12 Union Road, Cambridge CB2 1EZ, UK; fax: +44 1223 336033

Structure Determination of 2. The solid-state molecular structure of **2** exhibits disorder of both triflimide anions in the asymmetric unit; the relative occupancies of these sites were refined with free variables. Various restraints and constraints (SIMU, DFIX, DANG) were used to model the disordered triflimide molecules and to maintain physically reasonable anisotropic displacement parameters and geometries. A solvent mask is applied that accounts for two and a half molecules of THF. All non-hydrogen atoms were refined anisotropically and the H atoms were treated as riding models.

A residual electron density peak was noted near atom S3 of the minor component of the disordered triflimide molecule. This peak could not be assigned in a chemically reasonable fashion and was left as-is. There are no indications in the data to suggest twinning.

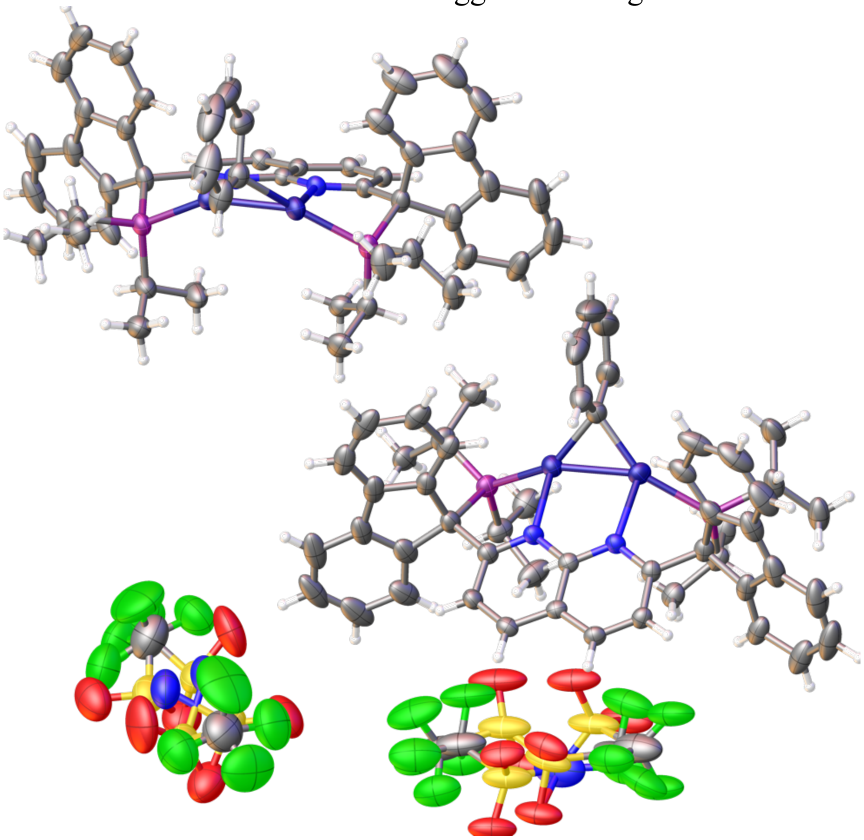

Figure S55. Solid-state molecular structure of **2**. Color scheme: C, gray; H, white; N, light blue; O, red; S, light yellow; F, green; P, purple; Cu, dark blue.

Structure Determination of 3. [(PNNP^{Flu})Cu_2(μ-O^{t}Bu)]$^+$ occupies a special position such that the asymmetric unit contains half a unit of [(PNNP^{Flu})Cu_2(μ-O^{t}Bu)]$^+$. The complete molecule is generated across the mirror planes. The solid-state molecular structure of **3** exhibits disorder of the triflimide anion across two different special positions with additional positional disorder about one of the special positions; the relative occupancies of these sites were refined with a free variable. Various restraints and constraints (SADI, RIGU, SIMU, AFIX, DFIX, DANG, EADP, ISOR) were used to model the disordered triflimide molecule and to maintain physically reasonable anisotropic displacement parameters and geometries. A solvent mask is applied that accounts for half of a molecule of THF. All non-hydrogen atoms were refined anisotropically and the H atoms were treated as riding models.

A residual negative density peak was noted near atom F1 of one of the bistriflimide anions. This negative density could not be assigned in a chemically reasonable fashion and was left as-is. There are no indications in the data to suggest twinning.

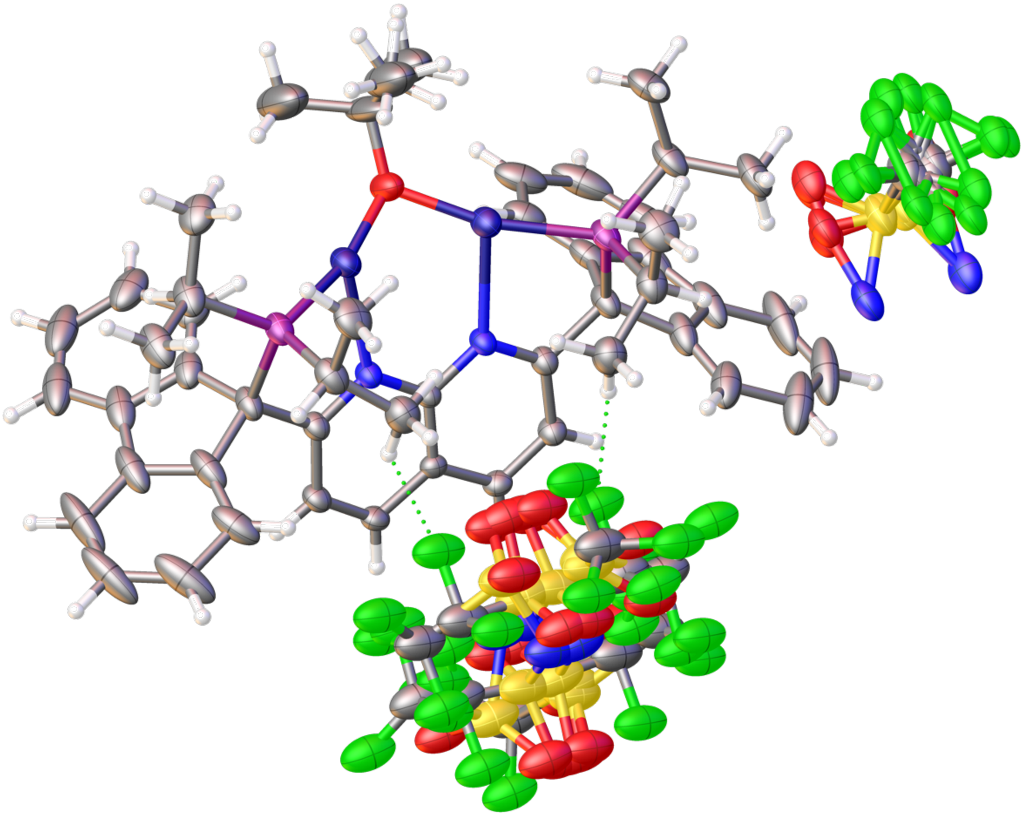

Figure S56. Solid-state molecular structure of **3**. Color scheme: C, gray; H, white; N, light blue; O, red; S, light yellow; F, green; P, purple; Cu, dark blue.

Structure Determination of 4. The solid-state molecular structure of **4** exhibits disorder of triflimide anion in the asymmetric unit; the relative occupancies of these sites were refined with free variables. Various restraints and constraints (SIMU, DFIX, SADI, EADP, DANG) were used to model the disordered triflimide molecule and to maintain physically reasonable anisotropic displacement parameters and geometries. One molecule of 1,2-difluorobenzene and water was modeled in the asymmetric unit. All non-hydrogen atoms were refined anisotropically and the H atoms were treated as riding models with the exception of the H atoms of the water molecule which were not modeled.

Missing H-atoms are noted for atom O9 of the one water molecule. H-atoms could not be reasonably located, assigned, or refined in the model as there is no clear H-bonding interactions. As such, these H-atoms were omitted from the model and left as-is.

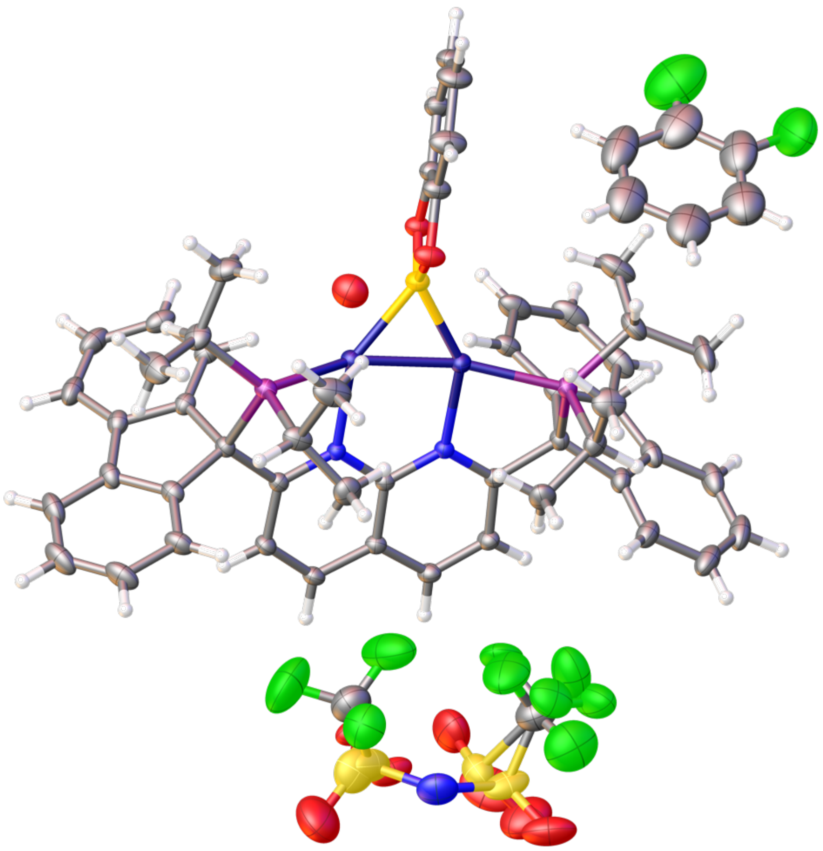

Figure S57. Solid-state molecular structure of **4**. Color scheme: C, gray; H, white; N, light blue; B; dark yellow; O, red; S, light yellow; F, green; P, purple; Cu, dark blue.

Structure Determination of 5. One molecule of dioxane was modeled in the asymmetric unit. All non-hydrogen atoms were refined anisotropically and the H atoms were treated as riding models.

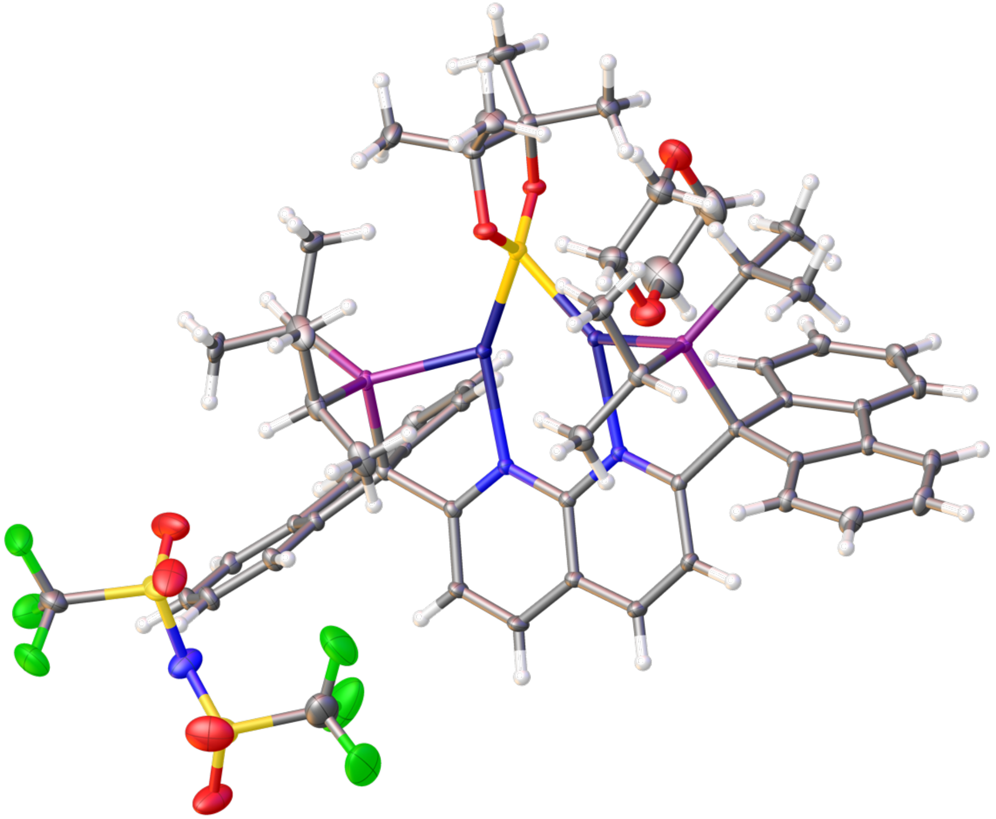

Figure S58. Solid-state molecular structure of **5**. Color scheme: C, gray; H, white; N, light blue; B; dark yellow; O, red; S, light yellow; F, green; P, purple; Cu, dark blue.

Structure Determination of 6. [(PNNP^{Flu})Cu_2(μ-CCPh)]$^+$ occupies a special position such that the asymmetric unit contains half a unit of [(PNNP^{Flu})Cu_2(μ-CCPh)]$^+$. The complete molecule is generated across the mirror planes. The solid-state molecular structure of **6** exhibits disorder of triflimide anion; the relative occupancies of these sites were refined with a free variable. Various restraints and constraints (SADI, RIGU, SIMU, DFIX, DANG, ISOR) were used to model the disordered triflimide molecule and to maintain physically reasonable anisotropic displacement parameters and geometries. Additionally, molecules of the triflimide are positionally disordered at the edge of the asymmetric unit on a special position. All non-hydrogen atoms were refined anisotropically and the H atoms were treated as riding models.

A residual negative density peak was noted near atom Cu1 of cationic fragment of the molecule. This negative density likely stems from the symmetry generation of the second half of the fragment.

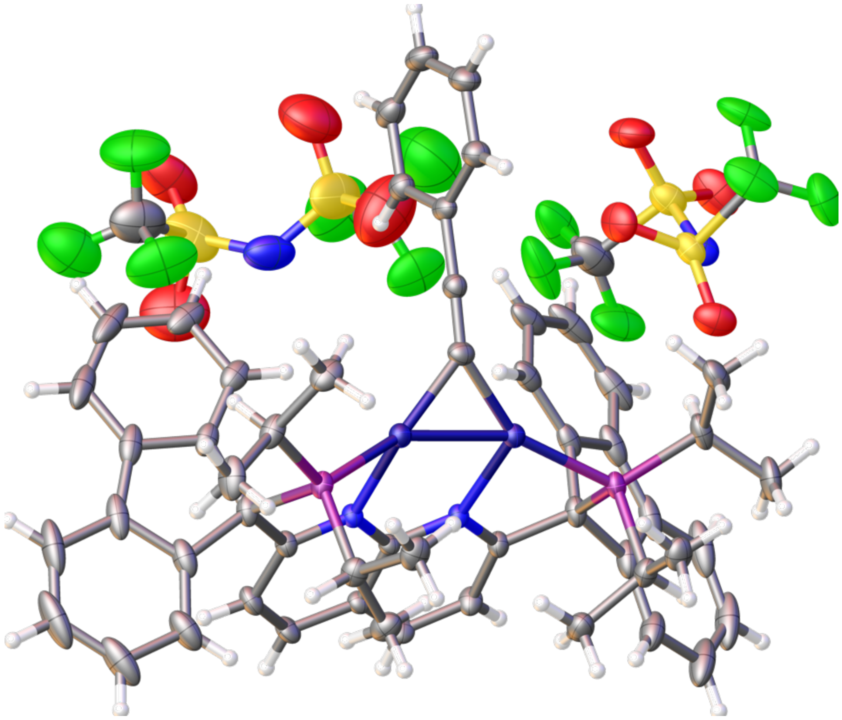

Figure S59. Solid-state molecular structure of **6**. Color scheme: C, gray; H, white; N, light blue; O, red; S, light yellow; F, green; P, purple; Cu, dark blue.

Structure Determination of 7. The solid-state molecular structure of **7** exhibits disorder of triflimide anion and one isopropyl group in the asymmetric unit; the relative occupancies of these sites were refined with free variables. Various restraints and constraints (SIMU, DANG) were used to model the disordered triflimide molecule and to maintain physically reasonable anisotropic displacement parameters and geometries. One molecule of THF and one molecule of pentane were modeled in the asymmetric unit. All non-hydrogen atoms were refined anisotropically and the H atoms were treated as riding models.

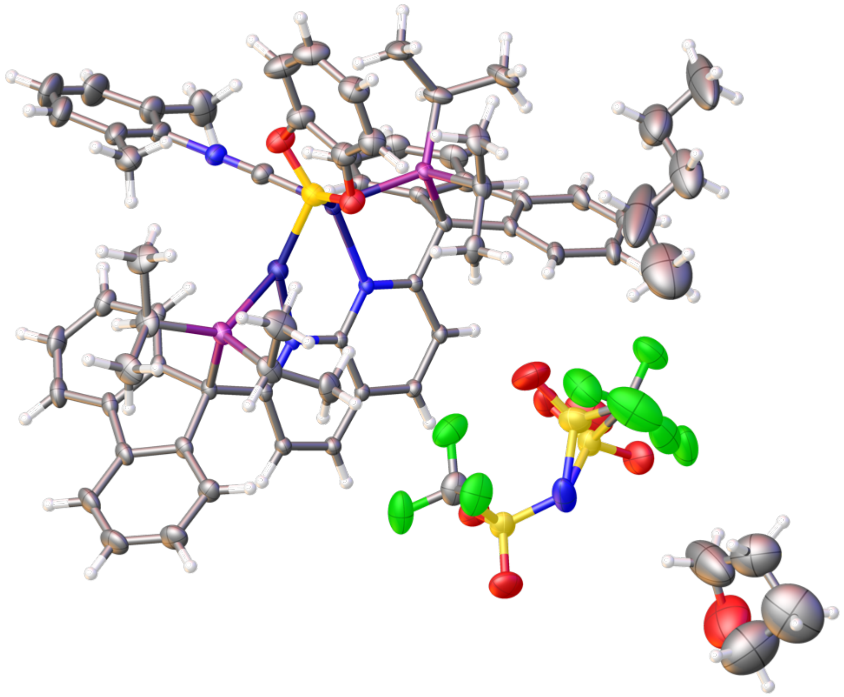

Figure S60. Solid-state molecular structure of **7**. Color scheme: C, gray; H, white; N, light blue; B; dark yellow; O, red; S, light yellow; F, green; P, purple; Cu, dark blue.

Structure Determination of 8. The solid-state molecular structure of **8** exhibits disorder of triflimide anion in the asymmetric unit; the relative occupancies of these sites were refined with a free variable. Various restraints and constraints (SADI, SIMU, DFIX) were used to model the disordered triflimide molecule and to maintain physically reasonable anisotropic displacement parameters and geometries. One molecule of 1,2-difluorobenzene was modeled in the asymmetric unit with positional disorder; the relative occupancies of these sites were refined with a free variable. All non-hydrogen atoms were refined anisotropically and the H atoms were treated as riding models

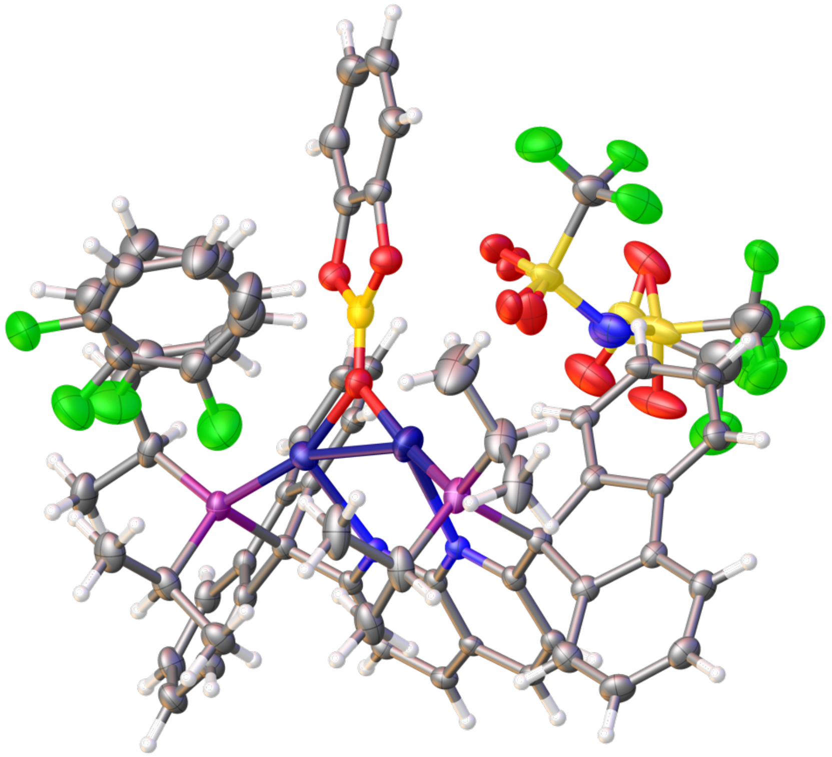

Figure S61. Solid-state molecular structure of **8**. Color scheme: C, gray; H, white; N, light blue; B; dark yellow; O, red; S, light yellow; F, green; P, purple; Cu, dark blue.

Structure Determination of 9. The solid-state molecular structure of **9** exhibits disorder of the triflimide anion, one isopropyl group, and the pinacol fragment in the asymmetric unit; the relative occupancies of these sites were refined with free variables. Various restraints and constraints (SADI, RIGU, SIMU, DFIX, DANG) were used to model the disordered triflimide molecule and to maintain physically reasonable anisotropic displacement parameters and geometries. Two molecules of THF were modeled in the asymmetric unit, with one positionally disordered; the relative occupancies of these sites were refined with a free variable. All non-hydrogen atoms were refined anisotropically and the H atoms were treated as riding models

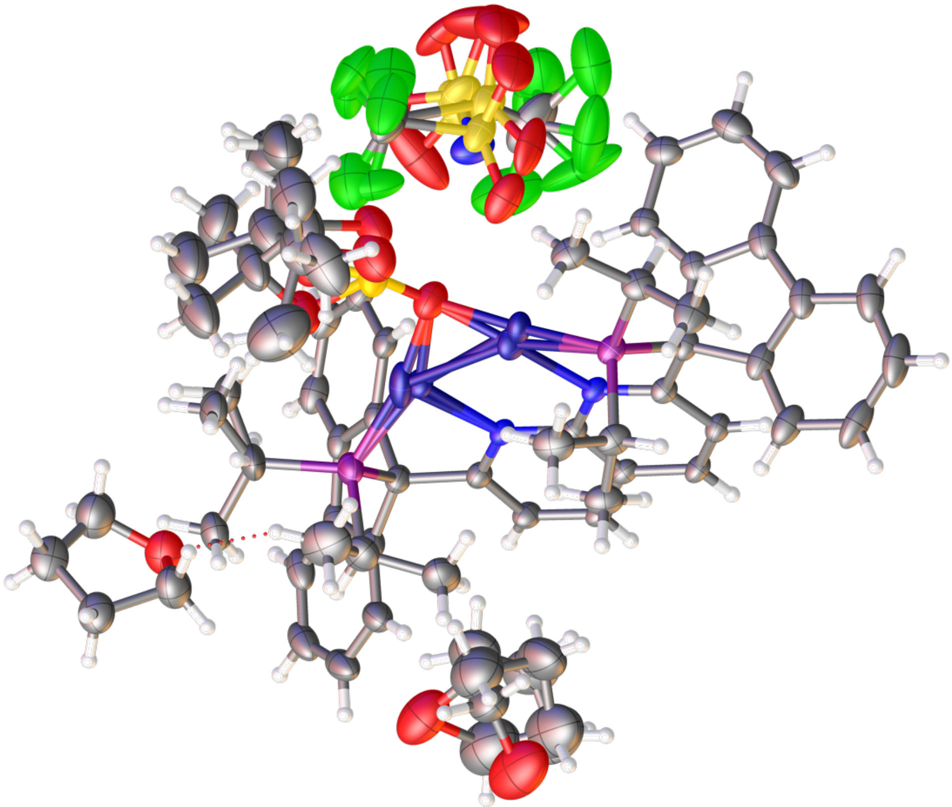

Figure S62. Solid-state molecular structure of **9**. Color scheme: C, gray; H, white; N, light blue; B; dark yellow; O, red; S, light yellow; F, green; P, purple; Cu, dark blue.

Structure Determination of 10. The solid-state molecular structure of **10** exhibits disorder of both triflimide anions and one isopropyl group in the asymmetric unit; the relative occupancies of these sites were refined with free variables. Various restraints and constraints (SADI, SIMU, DFIX, DANG) were used to model the disordered triflimide molecules and to maintain physically reasonable anisotropic displacement parameters and geometries. One molecule of THF was modeled in the asymmetric unit. A solvent mask is applied that accounts for seven additional molecules of THF. All non-hydrogen atoms were refined anisotropically and the H atoms were treated as riding models.

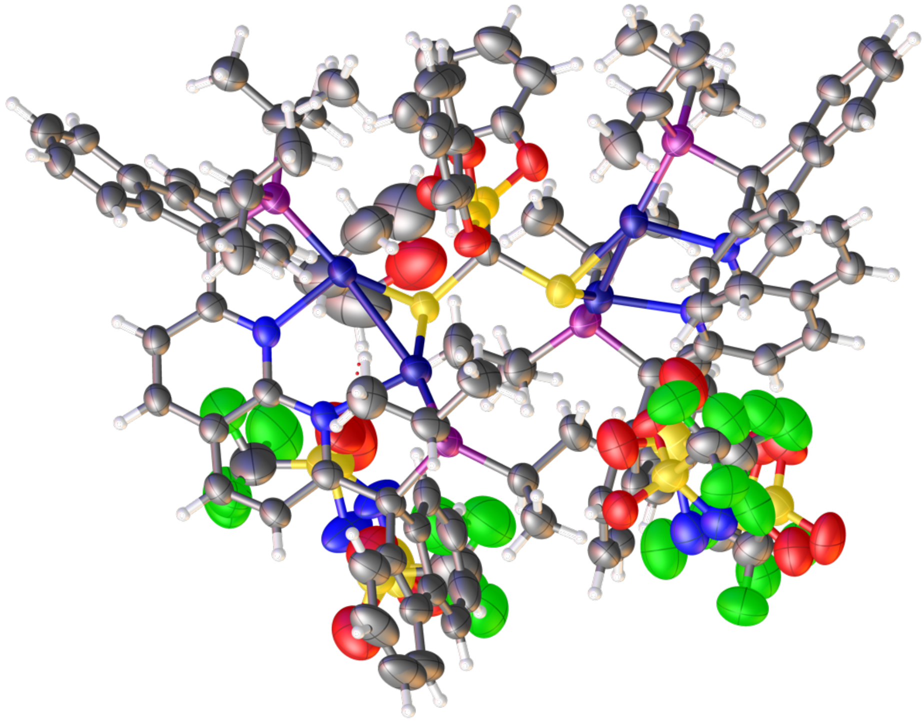

Figure S63. Solid-state molecular structure of **10**. Color scheme: C, gray; H, white; N, light blue; B; dark yellow; O, red; S, light yellow; F, green; P, purple; Cu, dark blue.

Structure Determination of 11. The solid-state molecular structure of **11** exhibits disorder of both triflimide anions in the asymmetric unit, one isopropyl group, and one of the pinacol fragments; the relative occupancies of these sites were refined with free variables. Various restraints and constraints (SADI, SIMU, DFIX, DANG, ISOR) were used to model the disordered triflimide molecules and to maintain physically reasonable anisotropic displacement parameters and geometries. All non-hydrogen atoms were refined anisotropically and the H atoms were treated as riding models.

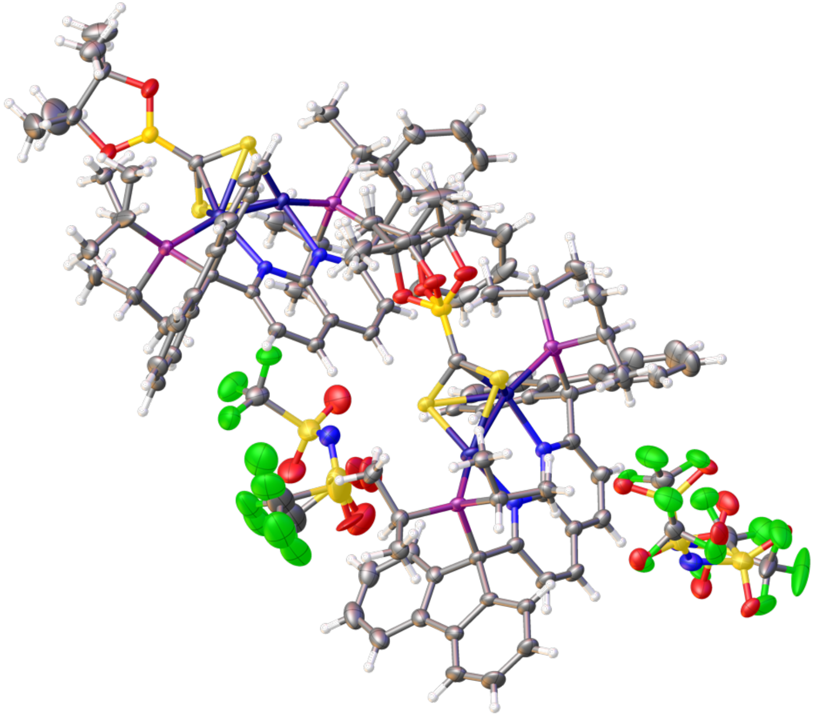

Figure S64. Solid-state molecular structure of **11**. Color scheme: C, gray; H, white; N, light blue; B; dark yellow; O, red; S, light yellow; F, green; P, purple; Cu, dark blue.

Table S1. Crystallographic and refinement data for compounds **2-5**.

Compound	2	3	4	5
**CCDC Identifier**	2323746	2323740	2323739	2323741
**Empirical formula**	$C_{116}H_{122}Cu_4F_{12}N_6O_{10}P_4S_4$	$C_{28}H_{32.5}CuF_3N_{1.5}O_3PS$	$C_{60}H_{56}BCu_2F_8N_3O_7P_2S_2$	$C_{58}H_{68}BCu_2F_6N_3O_8P_2S_2$
**Formula weight**	2494.47	621.62	1347.02	1313.10
**Temperature/K**	100.15	100.15	100.15	100.15
**Crystal system**	monoclinic	orthorhombic	monoclinic	monoclinic
**Space group**	C2/c	Pnnm	$P2_1/n$	$P2_1/n$
**a/Å**	24.139(2)	22.842(2)	15.0102(15)	15.1766(17)
**b/Å**	38.835(4)	11.5297(12)	18.1618(18)	26.358(3)
**c/Å**	25.983(4)	21.400(2)	22.003(2)	16.4567(18)
**α/°**	90	90	90	90
**β/°**	117.598(4)	90	97.280(3)	114.775(3)
**γ/°**	90	90	90	90
**Volume/Å^3**	21586(4)	5636.1(10)	5950.1(10)	5977.2(12)
**Z**	8	8	4	4
**Q_{calc}g/cm^3**	1.535	1.465	1.504	1.459
**u/mm^{-1}**	1.061	1.017	0.976	0.966
**F(000)**	10304.0	2476.0	2760.0	2720.0
**Crystal size/mm^3**	0.298 × 0.25 × 0.152	0.301 × 0.27 × 0.184	0.346 × 0.296 × 0.193	0.318 × 0.294 × 0.264
**Radiation**	synchrotron (λ = 0.7288)	synchrotron (λ = 0.7288)	synchrotron (λ = 0.7288)	synchrotron (λ = 0.7288)
**2Θ range for data collection/°**	2.15 to 53.012	2.674 to 62.852	2.992 to 62.882	3.522 to 62.864
**Index ranges**	$-29 \le h \le 29$, $-47 \le k \le 47$, $-31 \le l \le 31$	$-32 \le h \le 32$, $-16 \le k \le 16$, $-30 \le l \le 30$	$-21 \le h \le 21$, $-25 \le k \le 25$, $-31 \le l \le 31$	$-21 \le h \le 21$, $-37 \le k \le 37$, $-23 \le l \le 23$
**Reflections collected**	150042	83937	91592	97379
**Independent reflections**	20657 [R_{int} = 0.0686, R_{sigma} = 0.0456]	8703 [R_{int} = 0.0866, R_{sigma} = 0.0453]	18105 [R_{int} = 0.0927, R_{sigma} = 0.0615]	18297 [R_{int} = 0.0946, R_{sigma} = 0.0656]
**Data/restraints/ parameters**	20657/885/1531	8703/604/389	18105/844/851	18297/0/751
**Goodness-of-fit on F^2**	1.030	1.547	1.048	1.059
**Final R indexes [[>=2σ (I)]**	R_1 = 0.0827, wR_2 = 0.2468	R_1 = 0.0918, wR_2 = 0.3171	R_1 = 0.0715, wR_2 = 0.2191	R_1 = 0.0452, wR_2 = 0.1202
**Final R indexes [all data]**	R_1 = 0.1001, wR_2 = 0.2649	R_1 = 0.1010, wR_2 = 0.3296	R_1 = 0.0791, wR_2 = 0.2266	R_1 = 0.0530, wR_2 = 0.1259
**Largest diff. peak/hole / e Å^{-3}**	2.68/-1.21	1.45/-2.04	1.72/-1.86	0.65/-0.78

Table S2. Crystallographic and refinement data for compounds **7-8**.

Compound	6	7	8
**CCDC Identifier**	2323745	2323738	2323747
**Empirical formula**	$C_{28}H_{26.5}CuF_3N_{1.5}O_2PS$	$C_{72}H_{81}BCu_2F_6N_4O_7P_2S_2$	$C_{60}H_{56}BCu_2F_8N_3O_7P_2S_2$
**Formula weight**	599.58	1492.35	1347.02
**Temperature/K**	100.15	100.15	100.15
**Crystal system**	orthorhombic	triclinic	triclinic
**Space group**	Pnnm	P-1	P-1
**a/Å**	23.298(3)	13.9502(15)	14.7024(7)
**b/Å**	12.3553(14)	16.5677(17)	14.8315(7)
**c/Å**	18.881(2)	16.7482(17)	16.2802(8)
**α/°**	90	101.731(4)	116.702(2)
**β/°**	90	108.606(4)	93.75.1(2)
**γ/°**	90	98.807(4)	106.236(2)
**Volume/Å^3**	5435.2(10)	3491.4(6)	2968.5(3)
**Z**	8	2	2
**Q_{calc}g/cm^3**	1.465	1.420	1.507
**u/mm^{-1}**	1.049	0.836	0.921
**F(000)**	2464.0	1552.0	1380.0
**Crystal size/mm^3**	0.27 × 0.222 × 0.157	0.291 × 0.286 × 0.127	0.195 × 0.189 × 0.172
**Radiation**	synchrotron (λ = 0.7288)	synchrotron (λ = 0.7288)	MoKα (λ = 0.71073)
**2Θ range for data collection/°**	3.586 to 62.77	4.298 to 63.048	3.952 to 50.812
**Index ranges**	$-33 \le h \le 33$, $-17 \le k \le 17$, $-26 \le l \le 26$	$-19 \le h \le 19$, $-23 \le k \le 23$, $-23 \le l \le 23$	$-17 \le h \le 17$, $-17 \le k \le 17$, $-19 \le l \le 19$
**Reflections collected**	123148	60527	171409
**Independent reflections**	8525 [R_{int} = 0.1391, R_{sigma} = 0.0700]	21174 [R_{int} = 0.0516, R_{sigma} = 0.0595]	10903 [R_{int} = 0.0936, R_{sigma} = 0.0326]
**Data/restraints/ parameters**	8525/526/561	21174/139/923	10903/441/939
**Goodness-of-fit on F^2**	1.117	1.046	1.031
**Final R indexes [[>=2σ (I)]**	R_1 = 0.0614, wR_2 = 0.1635	R_1 = 0.0685, wR_2 = 0.1993	R_1 = 0.0415, wR_2 = 0.0974
**Final R indexes [all data]**	R_1 = 0.0738, wR_2 = 0.1729	R_1 = 0.0750, wR_2 = 0.2061	R_1 = 0.0585, wR_2 = 0.1046
**Largest diff. peak/hole / e Å^{-3}**	1.02/-2.13	2.02/-1.94	0.85/-0.67

Table S3. Crystallographic and refinement data for compounds **7-8**.

Compound	9	10	11
CCDC Identifier	2323743	2323742	2323744
Empirical formula	$C_{60}H_{72}BCu_2F_6N_3O_{8.5}P_2S_2$	$C_{141}H_{167}B_2Cu_4F_{12}N_6O_{20}P_4S_6$	$C_{130}H_{168}B_2Cu_4F_{12}N_6O_{12}P_4S_8$
Formula weight	1349.15	3085.82	2890.83
Temperature/K	100.15	100.15	100.15
Crystal system	monoclinic	triclinic	triclinic
Space group	$P2_1/n$	P-1	P-1
a/Å	15.2229(17)	18.9846(19)	14.0824(14)
b/Å	17.6988(19)	19.967(2)	17.9742(18)
c/Å	22.700(3)	21.054(2)	28.017(3)
α/°	90	66.367(4)	100.041(4)
β/°	97.203(4)	63.462(4)	104.212(4)
γ/°	90	79.477(4)	105.538(4)
Volume/Å^3	6067.6(12)	6541.1(12)	6400.7(11)
Z	4	2	2
Q_{calc}g/cm^3	1.477	1.567	1.500
u/mm^{-1}	0.954	0.931	0.974
F(000)	2800.0	3210.0	3016.0
Crystal size/mm^3	0.254 × 0.251 × 0.174	0.3 × 0.183 × 0.16	0.244 × 0.235 × 0.171
Radiation	synchrotron (λ = 0.7288)	synchrotron (λ = 0.7288)	synchrotron (λ = 0.7288)
2Θ range for data collection/°	3.002 to 52.95	2.282 to 52.36	1.592 to 58.318
Index ranges	-18 ≤ h ≤ 18, -21 ≤ k ≤ 21, -27 ≤ l ≤ 27	-22 ≤ h ≤ 22, -24 ≤ k ≤ 24, -25 ≤ l ≤ 25	-18 ≤ h ≤ 18, -23 ≤ k ≤ 23, -37 ≤ l ≤ 37
Reflections collected	105898	77706	93625
Independent reflections	11597 [R_{int} = 0.0860, R_{sigma} = 0.0514]	24168 [R_{int} = 0.0566, R_{sigma} = 0.0690]	31780 [R_{int} = 0.0559, R_{sigma} = 0.0608]
Data/restraints/ parameters	11597/415/1059	24168/509/1654	31780/711/1695
Goodness-of-fit on F^2	1.028	1.054	1.084
Final R indexes [[>=2σ (I)]	R_1 = 0.0577, wR_2 = 0.1587	R_1 = 0.0823, wR_2 = 0.2558	R_1 = 0.0518, wR_2 = 0.1525
Final R indexes [all data]	R_1 = 0.0649, wR_2 = 0.1633	R_1 = 0.1055, wR_2 = 0.2808	R_1 = 0.0652, wR_2 = 0.1616
Largest diff. peak/hole / e Å^{-3}	1.46/-0.79	0.98/-1.01	1.18/-0.87

% Buried Volume Calculations

General Considerations: SambVca 2.1 was used to conduct % buried volume calculations.[11] Coordinates of complex **4**, **5**, and $[(DPFN)Cu_2(\mu\text{-}Bcat)]^+$ were supplied from DFT-optimized geometries.[7] Coordinates of $\{[(SIPr)Cu]_2(\mu\text{-}Bcat)\}^+$ were supplied from crystallographic sources.[12]

%V Free	%V Buried	% V Tot/V Ex
28.2	71.8	99.9

Quadrant	V f	V b	V t	%V f	%V b
SW	12.4	32.4	44.9	27.7	72.3
NW	12.4	32.5	44.9	27.7	72.3
NE	12.9	32.0	44.9	28.7	71.3
SE	12.9	32.0	44.9	28.7	71.3

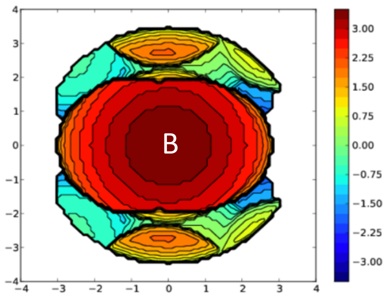

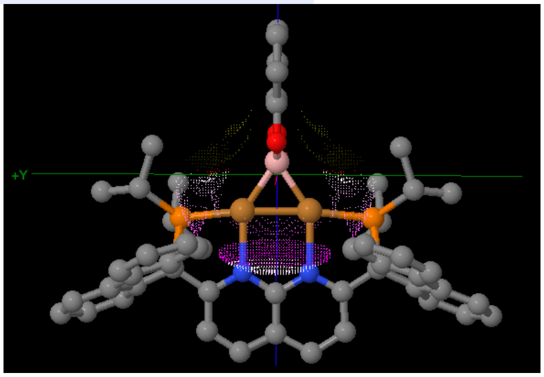

Figure S65. % Buried Volume on B in complex **3**.

%V Free	%V Buried	% V Tot/V Ex
19.8	80.2	99.9

Quadrant	V f	V b	V t	%V f	%V b
SW	5.6	39.3	44.9	12.4	87.6
NW	6.8	38.0	44.9	15.2	84.8
NE	9.7	35.1	44.9	21.7	78.3
SE	13.3	31.5	44.9	29.8	70.2

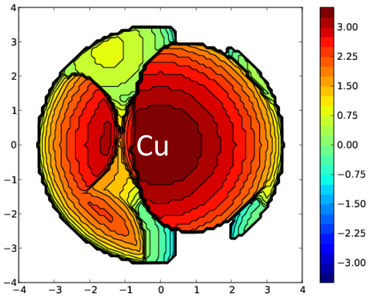

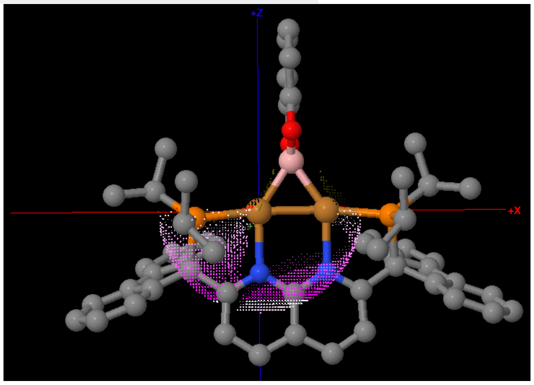

Figure S66. % Buried Volume on Cu in complex **3**.

%V Free	%V Buried	% V Tot/V Ex
27.8	**72.2**	99.9

Quadrant	V f	V b	V t	%V f	%V b
SW	14.9	29.9	44.9	**33.3**	66.7
NW	6.2	38.7	44.9	**13.8**	86.2
NE	17.3	27.6	44.9	**38.5**	61.5
SE	11.4	33.4	44.9	**25.4**	74.6

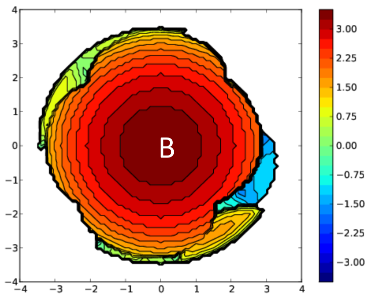

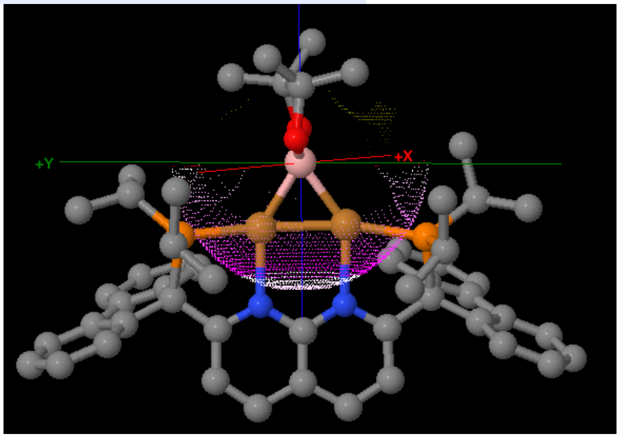

Figure S67. % Buried Volume on B in complex **4**.

%V Free	%V Buried	% V Tot/V Ex
19.6	**80.4**	99.9

Quadrant	V f	V b	V t	%V f	%V b
SW	6.5	38.4	44.9	**14.4**	85.6
NW	6.4	38.4	44.9	**14.3**	85.7
NE	11.0	33.9	44.9	**24.4**	75.6
SE	11.4	33.5	44.9	**25.3**	74.7

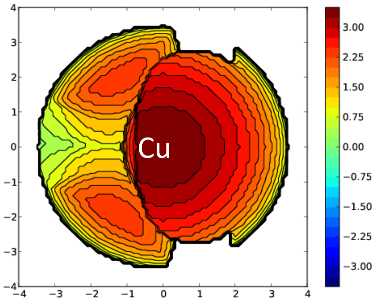

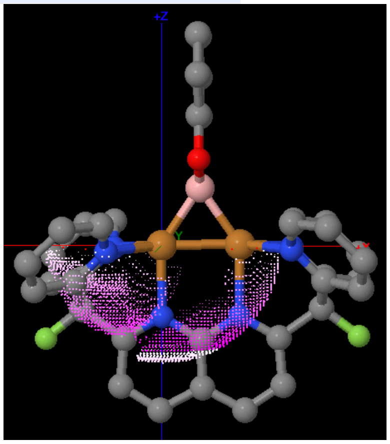

Figure S68. % Buried Volume on Cu in [(DPFN)Cu_2(μ-Bcat)]$^+$.

%V Free	%V Buried	% V Tot/V Ex
18.1	**81.9**	99.9

Quadrant	V f	V b	V t	%V f	%V b
SW	12.6	32.3	44.9	**28.0**	72.0
NW	12.4	32.5	44.9	**27.6**	72.4
NE	5.9	39.0	44.9	**13.1**	86.9
SE	1.7	43.1	44.9	**3.9**	96.1

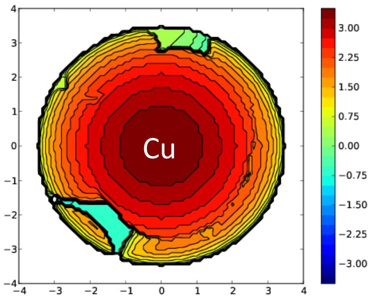

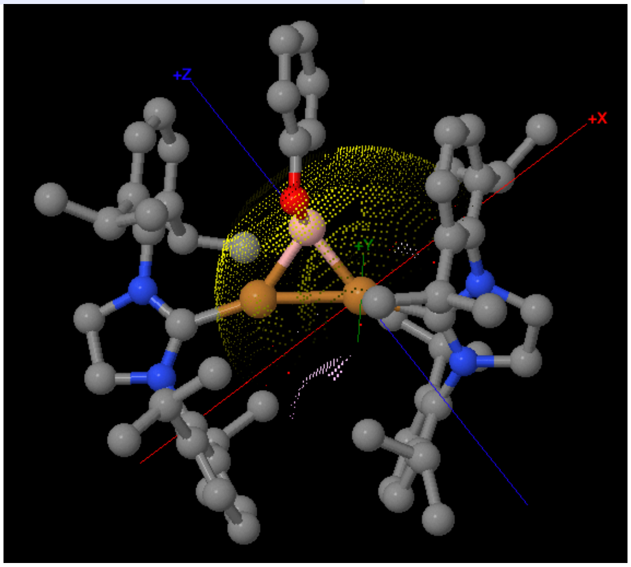

Figure S69. % Buried Volume on Cu in $\{[(SIPr)Cu]_2(\mu\text{-}Bcat)\}^+$

Computational Details

DFT calculations were performed at the Molecular Graphics and Computation Facility of UC Berkeley, using the Gaussian 09 suite of programs.[13] The geometry of the [(PNNP^{Flu})Cu_2(μ-Bpin)]$^+$, [(PNNP^{Flu})Cu_2(μ-Bcat)]$^+$ fragments was optimized in the gas phase without restrictions using the PBE0 functional,[14] as implemented in the G09 software along with Grimme's dispersion correction with Becke-Johnson damping function (D3(BJ)).[15] Non-metal atoms were described with the 6-31g(d,p) basis set[16] and the Cu atoms were modeled with the Stuttgart/Dresden SDD basis set and its associated effective core potential.[17] Vibrational analysis was used to characterize the stationary point in the potential energy surface. Natural Bond Order (NBO) and Natural Localized Molecular Orbital (NLMO) analyses were performed on the optimized structures using NBO 6.[18] Structure and orbital visualization was performed with Chemcraft software.[19]

Selected Natural Localized Molecular Orbitals

Complex **4**

Hybridization/Polarization Analysis of NLMOs in NAO Basis:
NLMO / Occupancy / Percent from Parent NBO / Atomic Hybrid Contributions

```
96. (2.00000)   81.8295% BD ( 1)Cu  1- B 55
                 16.694% Cu  1 s( 96.88%)p 0.01(  0.55%)d 0.03(  2.57%)
                 16.284% Cu  2 s( 97.00%)p 0.01(  0.56%)d 0.03(  2.44%)
                 65.231%  B 55 s( 51.19%)p 0.95( 48.79%)d 0.00(  0.02%)
```

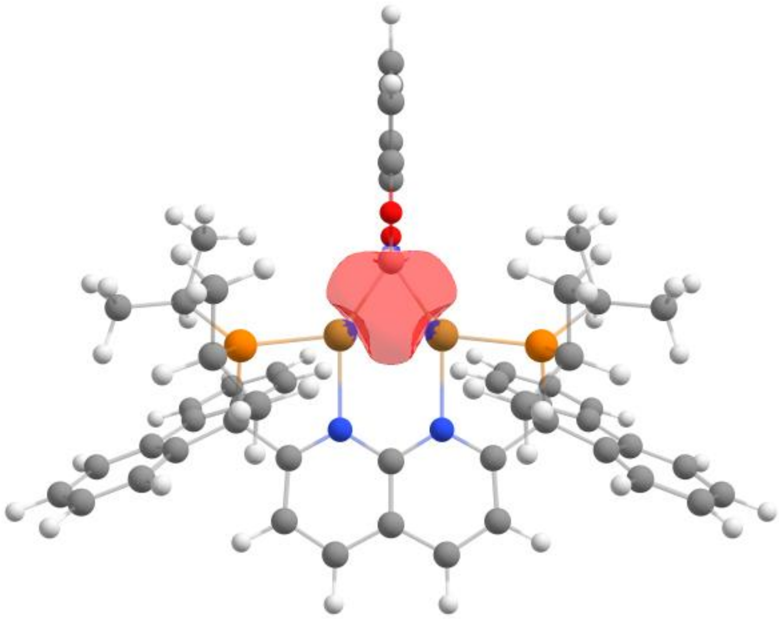

Figure S70. NLMO 96 of Complex **4**.

Complex **5**

Hybridization/Polarization Analysis of NLMOs in NAO Basis:

NLMO / Occupancy / Percent from Parent NBO / Atomic Hybrid Contributions

96. (2.00000)   81.6018% BD (1)Cu 2- B112
    16.273% Cu 1 s(96.87%)p 0.01(0.67%)d 0.03(2.46%)
    17.616% Cu 2 s(96.92%)p 0.01(0.55%)d 0.03(2.53%)
    64.063% B112 s(47.43%)p 1.11(52.54%)d 0.00(0.02%)

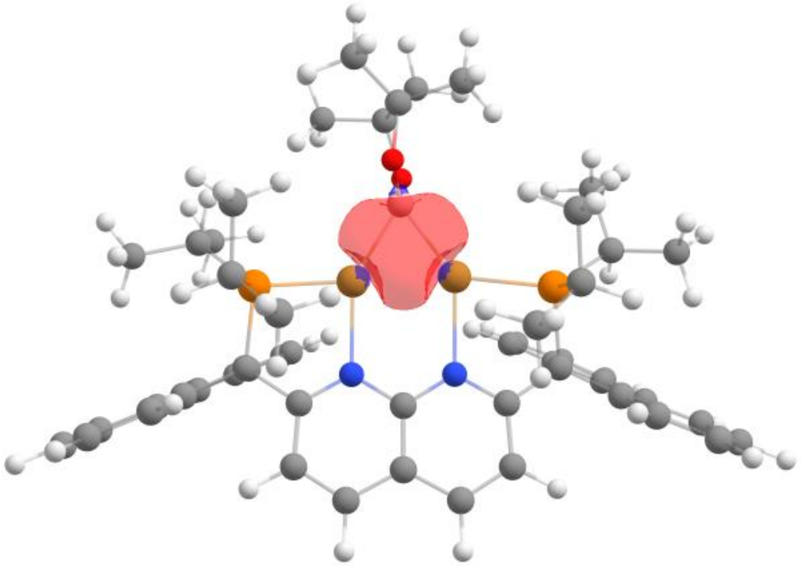

Figure S71. NLMO 96 of Complex **5**.

References

1. Newkome, G. R.; Garbis, S. J.; Majestic, V. K.; Fronczek, F. R.; Chiari, G. *J. Org. Chem.* **1981**, *46*, 833.
2. Brooks, N. R.; Schaltin, S.; Van Hecke, K.; Van Meervelt, L.; Binnemans, K.; Fransaer, J. *Chem. – Eur. J.* **2011**, *17*, 5054.
3. Wang, Y.; Zhang, W. X.; Wang, Z.; Xi, Z. *Angew. Chem. Int. Ed.* **2011**, *50*, 8122–8126.
4. Fulmer, G. R.; Miller, A. J.; Sherden, N. H.; Gottlieb, H. E.; Nudelman, A.; Stoltz, B. M.; Bercaw, J. E.; Goldberg, K. I. *Organometallics* **2010**, *29*, 2176–2179.
5. Dokken, H. J.; Frenette, B. L.; Ferguson, M. J.; Rivard, E. *Eur. J. Inorg. Chem.* **2023**. DOI:10.1002/ejic.202300202.
6. Ostoja-Starzewski, K. H. A.; Xin, B. S. US6657027, 2003, B2
7. Ríos, P.; See, M. S.; Handford, R. C.; Teat, S. J.; Tilley, T. D. *Chem. Sci.* **2022**, *13*, 6619–6625.
8. Sheldrick, G. M. SHELXT– Integrated Space-Group and Crystal-Structure Determination. Acta Crystallogr. 2015, A71, 3–8
9. Sheldrick, G. M. Crystal Structure Refinement with SHELXL. Acta Cryst 2015, C71, 3–8.
10. Dolomanov, O. V.; Bourhis, L. J.; Gildea, R. J.; Howard, J. A. K.; Puschmann, H. OLEX2: A Complete Structure Solution, Refinement and Analysis Program. J Appl Cryst 2009, 42, 339–341.
11. SambVca 2.1 was used to calculate %Vbur: Falivene, L.; Cao, Z.; Petta, A.; Serra, L.; Poater A.; Oliva, R.; Scarano, V.; Cavallo, L. *Nat. Chem*. **2019**, *11*, 872–879.
12. Wyss, C. M.; Bitting, J.; Bacsa, J.; Gray, T. G.; Sadighi, J. P. *Organometallics* **2016**, *35*, 71–74.
13. Gaussian 09, Revision A.02, Frisch, M. J.; Trucks, G. W.; Schlegel, H. B.; Scuseria, G. E.; Robb, M. A.; Cheeseman, J. R.; Scalmani, G.; Barone, V.; Petersson, G. A.; Nakatsuji, H.; Li, X.; Caricato, M.; Marenich, A.; Bloino, J.; Janesko, B. G.; Gomperts, R.; Mennuci, B.; Hratchian, H. P.; Ortiz, J. V.; Izmaylov, A. F.; Sonnenberg, J. L.; Williams-Young, D.; Ding, F.; Lipparini, F.; Egidi, F.; Goings, J.; Peng, B.; Petrone, A.; Henderson, T.; Ranasinghe, D.; Zakrzewski, V. G.; Gao, J.; Rega, N.; Zheng, G.; Liang, W.; Hada, M.; Ehara, M.; Toyota, K.; Fukuda, R.; Hasegawa, J.; Ishida, M.; Nakajima, T.; Honda, Y.; Kitao, O.; Nakai, H.; Vreven, T.; Throssel, K.; Montgomery Jr, J. A.; Peralta, J. E.; Ogliaro, F.; Bearpark, M.; Heyd, J. J.; Brothers, E.; Kudin, K. N.; Staroverov, V. N.; Keith, T.; Kobayashi, R.; Normand, J.; Raghavachari, K.; Rendell, A.; Burant, J. C.; Iyengar, S. S.; Tomasi, J.; Cossi, M.; Millam, J. M.; Klene, M.; Adamo, C.; Cammi, R.; Ochterski, J. W.; Martin, R. L.; Morokuma, K.; Farkas, O.; Roseman, J. B.; Fox, D. J. Gaussian, Inc., Wallingford CT, 2016.
14. Adamo, C.; Barone, V. J. *Chem. Phys.* **1999**, *110*, 6158–6170.
15. Grimme, S.; Ehrlich, S.; Goerigk, L. *J. Comp. Chem*. **2011**, *32*, 1456–1465.
16. Hehre, W. J.; Ditchfield, R.; Pople, J. A. *J. Chem. Phys*. **1972**, *56*, 2257-2261; Hariharan, P. C.; Pople, J. A. *Theor. Chim. Acta.* **1973**, *28*, 213–222.
17. Dolg, M.; Wedig, U.; Stoll, H.; Preuss, H. *J. Chem. Phys*. **1987**, *86*, 866–872.
18. Glendening, E. D.; Landis, C. R.; Weinhold, F. NBO 6.0: Natural bond orbital analysis program. *J. Comput. Chem*. **2013**, *34*, 1429–1437.
19. Chemcraft - graphical software for visualization of quantum chemistry computations. https://www.chemcraftprog.com
